# Supplementary material for: FOXP1 phosphorylation antagonizes its O-GlcNAcylation in regulating ATR activation in response to replication stress
Source: EMBO J. 2024 Dec 2;44(2):457–83. doi: 10.1038/s44318-024-00323-x (PMC11729909; doi:10.1038/s44318-024-00323-x)
Supplement: Supplementary file 1 — Table EV1 [file 44318_2024_323_MOESM1_ESM.docx]

**Table EV1. Oligonucleotide sequences**

| **Name** | **sequence** |
| --- | --- |
| **PCR primers** |  |
| pcDNA3.0-FLAG-FOXP1 (human) PF | GATGACGATGACAAGGAATTCATGATGCAAGAATCTGGGAC |
| pcDNA3.0-FLAG-FOXP1 (human) PR | TATAGAATAGGGCCCTCTAGATCACTCCATGTCCTCGTTT |
| plenti-Blast-FOXP1(human) PF | GATGACGATGACAAGCTCGAGATGATGCAAGAATCTGGGAC |
| plenti-Blast-FOXP1(human) PR | AGGCTTACCTTCGAACCGCGGTCACTCCATGTCCTCGTTT |
| pGEX-4T-1-FOXP1(human) PF | CCGCGTGGATCCCCGGAATTCATGATGCAAGAATCTGGGAC |
| pGEX-4T-1-FOXP1(human) PR | GTCACGATGCGGCCGCTCGAGTCACTCCATGTCCTCGTTT |
| FOXP1 (human) R465G point mutation PF | AAGAACGCAGAAGTTGGACCACCATTTACATATGCATCTTTAAT |
| FOXP1 (human) R465G point mutation PR | ATATGTAAATGGTGGTCCAACTTCTGCGTTCTTATAAAATTCT |
| FOXP1 (human) R514C point mutation PF | TGGAAGAATGCAGTGTGTCATAATCTTAGTCTTCACAAGTGTT |
| FOXP1 (human) R514C point mutation PR | AAGACTAAGATTATGACACACTGCATTCTTCCACGTGGCC |
| FOXP1 (human) R465T point mutation PF | AAGAACGCAGAAGTTACACCACCATTTACATATGCATCTTTAAT |
| FOXP1 (human) R465T point mutation PR | ATATGTAAATGGTGGTGTAACTTCTGCGTTCTTATAAAATTCT |
| FOXP1 (human) R514H point mutation PF | TGGAAGAATGCAGTGCATCATAATCTTAGTCTTCACAAGTGTT |
| FOXP1 (human) R514H point mutation PR | AAGACTAAGATTATGATGCACTGCATTCTTCCACGTGGC |
| FOXP1 (human) S396A point mutation PF | CTCTCCAAGTCCGCAGCGGAGGCTTCTCCACAGAGCTTACCTC |
| FOXP1 (human) S396A point mutation PR | CTGTGGAGAAGCCTCCGCTGCGGACTTGGAGAGAGTGACACTT |
| FOXP1 (human) S396D point mutation PF | CTCTCCAAGTCCGCAGATGAGGCTTCTCCACAGAGCTTACCTC |
| FOXP1 (human) S396D point mutation PR | CTGTGGAGAAGCCTCATCTGCGGACTTGGAGAGAGTGACACTT |
| FOXP1 (human) T236A point mutation PF | CTCTGGAAAGAAGTGGCAAGTGCTCATACTGCAGAAGAAACC |
| FOXP1 (human) T236A point mutation PR | TGCAGTATGAGCACTTGCCACTTCTTTCCAGAGCTGCTGCAG |
| FOXP1 (human) S440A point mutation PF | ATCCGCAGGCGGTACGCAGACAAATACAACGTGCCCATTTCGT |
| FOXP1 (human) S440A point mutation PR | CACGTTGTATTTGTCTGCGTACCGCCTGCGGATGGGTCCCACC |
| FOXP1 (human) Δ2-230 mutation PF | GACAAGGAATTCATGCTCTGGAAAGAAGTGACAAG |
| FOXP1 (human) Δ2-230 mutation PR | CATGAATTCCTTGTCATCGT |
| FOXP1 (human) Δ231-369 mutation PF | ACAGAACTGCAGCAGCATGTGAAGTCTACAGAACC |
| FOXP1 (human) Δ231-369 mutation PR | CTGCTGCAGTTCTGTTGG |
| FOXP1 (human) Δ370-390 mutation PF | ATGATGACCCACCTGCTCTCCAAGTCCGCATCG |
| FOXP1 (human) Δ370-390 mutation PR | CAGGTGGGTCATCATGGC |
| FOXP1 (human) Δ391-410 mutation PF | GTATCAAGTGTCACTACCGCCCCCCTGACTCCC |
| FOXP1 (human) Δ391-410 mutation PR | AGTGACACTTGATACCAGATTC |
| FOXP1 (human) Δ411-430 mutation PF | ACTCCAACGACCCCAACGGTGGGACCCATCCGC |
| FOXP1 (human) Δ411-430 mutation PR | TGGGGTCGTTGGAGTATGAG |
| FOXP1 (human) Δ431-450 mutation PF | ACCACCAGCATGCACGATATTGCGCAGAACCAAG |
| FOXP1 (human) Δ431-450 mutation PR | GTGCATGCTGGTGGTTGT |
| FOXP1 (human) Δ451-464 mutation PF | CCCATTTCGTCAGCAAGACCACCATTTACATATGC |
| FOXP1 (human) Δ451-464 mutation PR | TGCTGACGAAATGGGCAC |
| FOXP1 (human) Δ465-555 mutation PF | AAGAACGCAGAAGTTATTAAAAACATGCAGAGCAGC |
| FOXP1 (human) Δ465-555 mutation PR | AACTTCTGCGTTCTTATAAAAT |
| FOXP1 (human) Δ556-610 mutation PF | GGTAACCCTTCCCTTGAGCATACCAACAGCAAC |
| FOXP1 (human) Δ556-610 mutation PR | AAGGGAAGGGTTACCACT |
| FOXP1 (human) Δ611-677 mutation PF | CTGAACGGGGCAATGTGATCTAGAGGGCCCTATT |
| FOXP1 (human) Δ611-677 mutation PR | CATTGCCCCGTTCAGCTC |
| FOXP1 (human) Δ556-570 mutation PF | GGTAACCCTTCCCTTGCAGCTTTACAGGCTTC |
| FOXP1 (human) Δ556-570 mutation PR | AAGGGAAGGGTTACCACT |
| FOXP1 (human) Δ571-590 mutation PF | TGCACACCTCTCAATGGAAATCCCACTCTGGG |
| FOXP1 (human) Δ571-590 mutation PR | ATTGAGAGGTGTGCAGTAG |
| FOXP1 (human) Δ591-610 mutation PF | ACTACCGCTTCCATGGAGCATACCAACAGCAAC |
| FOXP1 (human) Δ591-610 mutation PR | CATGGAAGCGGTAGTGTAT |
| **siRNA and shRNA** |  |
| siNC (Nonspecific) | UUCUCCGAACGUGUCACGU |
| siFOXP1 (human, CDS) | CUGGUUCACACGAAUGUUU |
| siFOXP1 (human, UTR) | GCAUUGGAUGGACUUGUUU |
| shFOXP1 (human, UTR) forward | CCGGGCATTGGATGGACTTGTTTCTCGAGAAACAAGTCCATCCAATGCTTTTTG |
| shFOXP1 (human, UTR) reverse | AATTCAAAAAGCATTGGATGGACTTGTTTCTCGAGAAACAAGTCCATCCAATGC |
