# Supplementary material for: FOXP1 phosphorylation antagonizes its O-GlcNAcylation in regulating ATR activation in response to replication stress
Source: EMBO J. 2024 Dec 2;44(2):457–83. doi: 10.1038/s44318-024-00323-x (PMC11729909; doi:10.1038/s44318-024-00323-x)
Supplement: Supplementary file 2 — Table EV2 [file 44318_2024_323_MOESM2_ESM.docx]

**Table EV2: STR profiles of cell lines**

|  | **NCI-H1975** | **HEK293** |
| --- | --- | --- |
| **STR loci** |  |  |
| AmeL | X, X | X, X |
| TH01 | 7,7 | 7, 9.3 |
| D5S818 | 11,12 | 8.8 |
| D13S317 | 10,10 | 12,14 |
| D7S820 | 8,11 | 11,12 |
| D16S539 | 9,12 | 9,13 |
| CSFIPO | 12,12 | 12,12 |
| vWA | 18,18 | 16,19 |
| TPOX | 8,11 | 11,11 |
