# Supplementary figures and images for: FOXP1 phosphorylation antagonizes its O-GlcNAcylation in regulating ATR activation in response to replication stress (part 1 of 3)
Source: EMBO J. 2024 Dec 2;44(2):457–83. doi: 10.1038/s44318-024-00323-x (PMC11729909; doi:10.1038/s44318-024-00323-x)

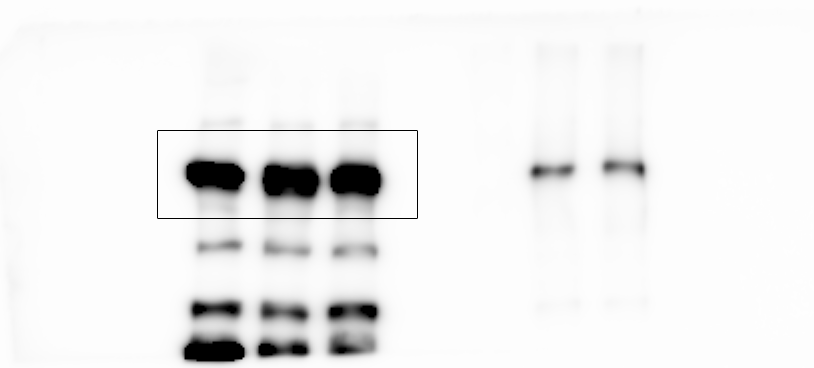

Supplement: Supplementary file 4 — Source data Fig. 1 [file 44318_2024_323_MOESM4_ESM.zip › SD figure 1/1B/western ATR INPUT.tif]

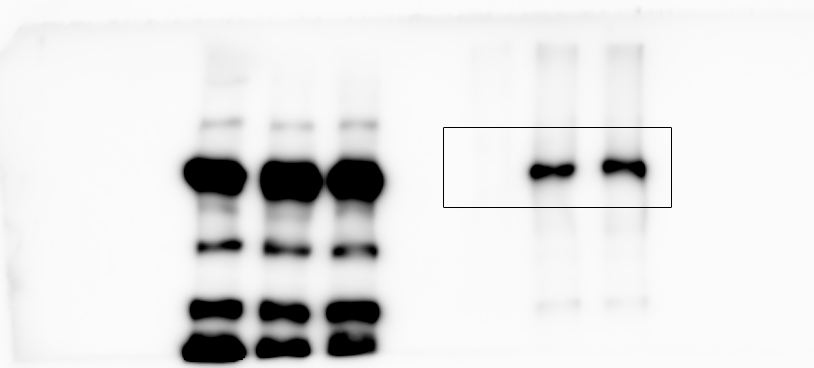

Supplement: Supplementary file 4 — Source data Fig. 1 [file 44318_2024_323_MOESM4_ESM.zip › SD figure 1/1B/western ATR IP.tif]

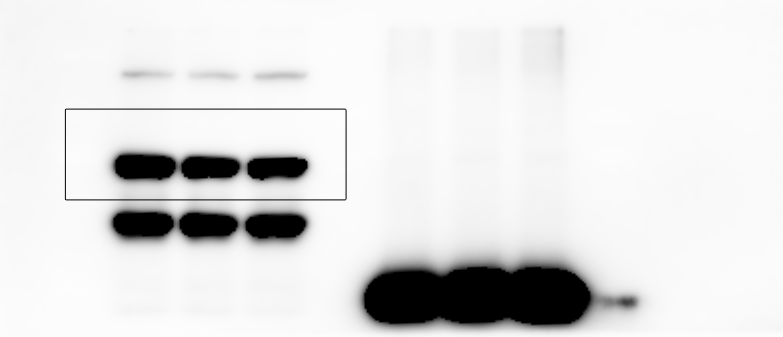

Supplement: Supplementary file 4 — Source data Fig. 1 [file 44318_2024_323_MOESM4_ESM.zip › SD figure 1/1B/western FOXP1 INPUT.tif]

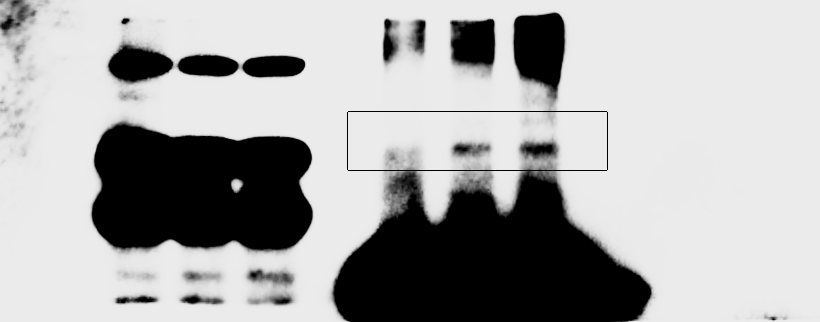

Supplement: Supplementary file 4 — Source data Fig. 1 [file 44318_2024_323_MOESM4_ESM.zip › SD figure 1/1B/western FOXP1 IP.tif]

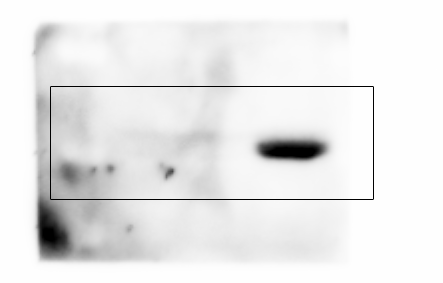

Supplement: Supplementary file 4 — Source data Fig. 1 [file 44318_2024_323_MOESM4_ESM.zip › SD figure 1/1B/western pCHK1S345.tif]

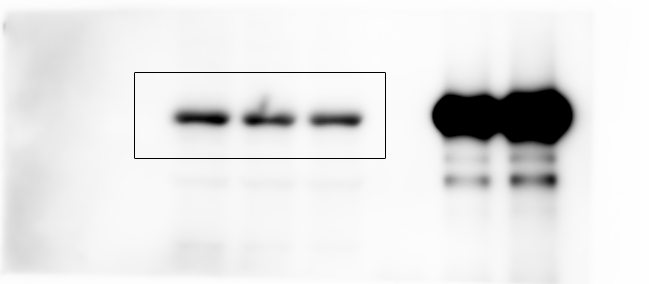

Supplement: Supplementary file 4 — Source data Fig. 1 [file 44318_2024_323_MOESM4_ESM.zip › SD figure 1/1C/western ATRIP INPUT.tif]

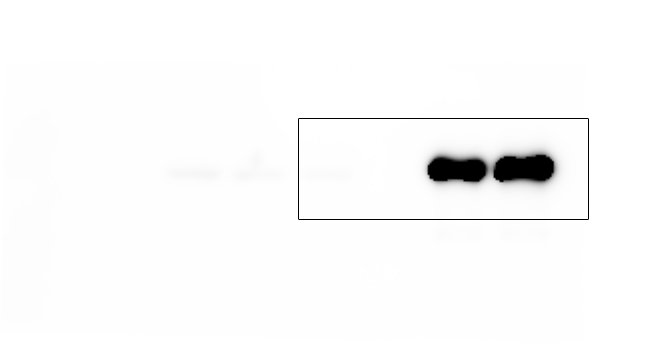

Supplement: Supplementary file 4 — Source data Fig. 1 [file 44318_2024_323_MOESM4_ESM.zip › SD figure 1/1C/western ATRIP IP.tif]

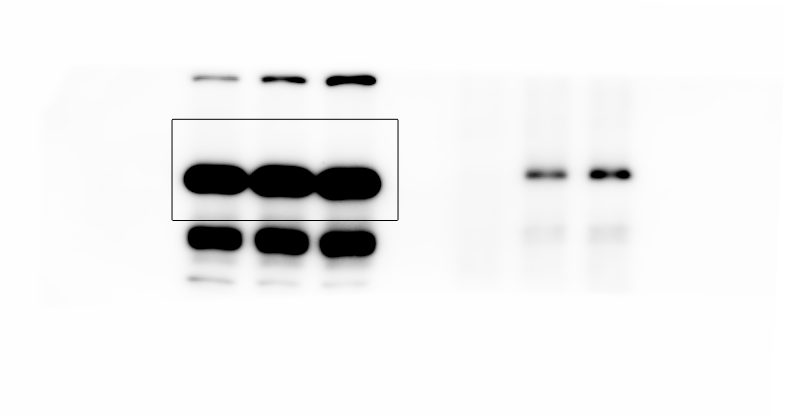

Supplement: Supplementary file 4 — Source data Fig. 1 [file 44318_2024_323_MOESM4_ESM.zip › SD figure 1/1C/western FOXP1 INPUT.tif]

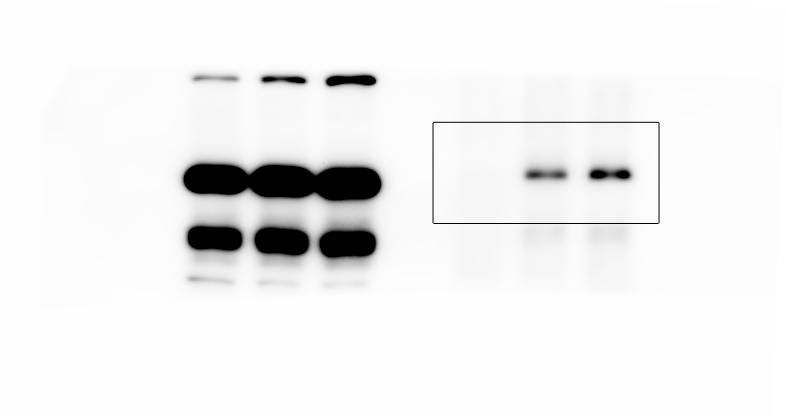

Supplement: Supplementary file 4 — Source data Fig. 1 [file 44318_2024_323_MOESM4_ESM.zip › SD figure 1/1C/western FOXP1 IP.tif]

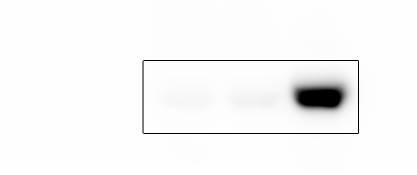

Supplement: Supplementary file 4 — Source data Fig. 1 [file 44318_2024_323_MOESM4_ESM.zip › SD figure 1/1C/western pCHK1S345 INPUT.tif]

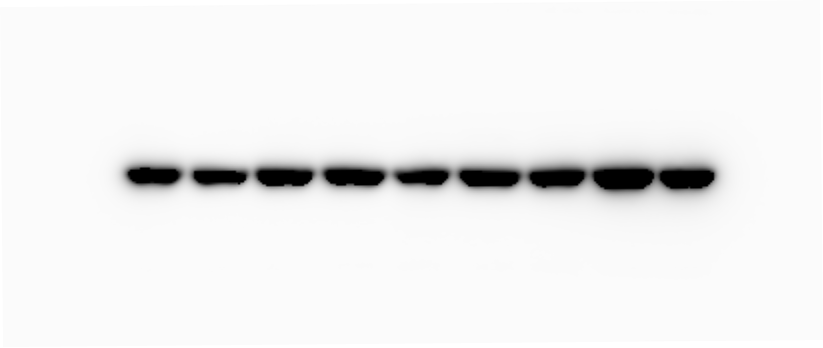

Supplement: Supplementary file 4 — Source data Fig. 1 [file 44318_2024_323_MOESM4_ESM.zip › SD figure 1/1D/western-Actin.tif]

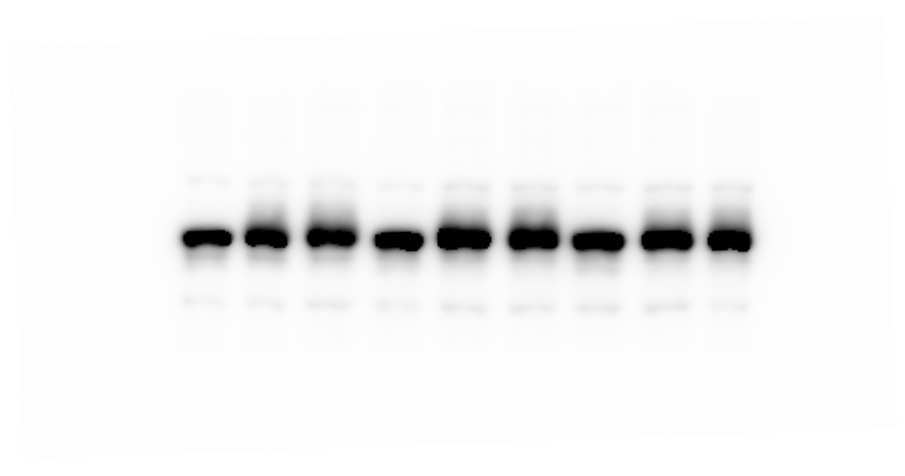

Supplement: Supplementary file 4 — Source data Fig. 1 [file 44318_2024_323_MOESM4_ESM.zip › SD figure 1/1D/western-CHK1.tif]

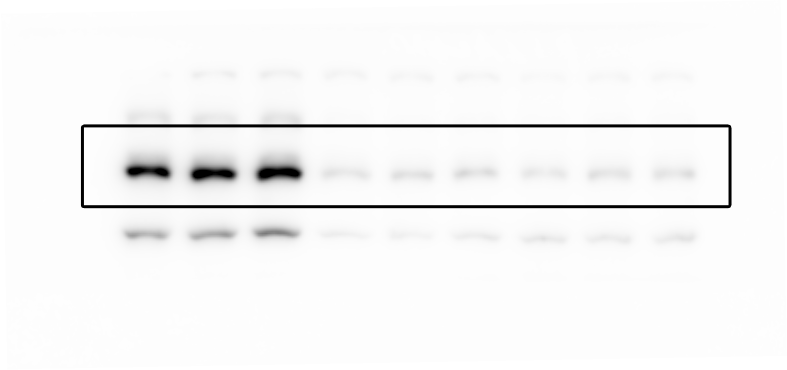

Supplement: Supplementary file 4 — Source data Fig. 1 [file 44318_2024_323_MOESM4_ESM.zip › SD figure 1/1D/western-FOXP1.tif]

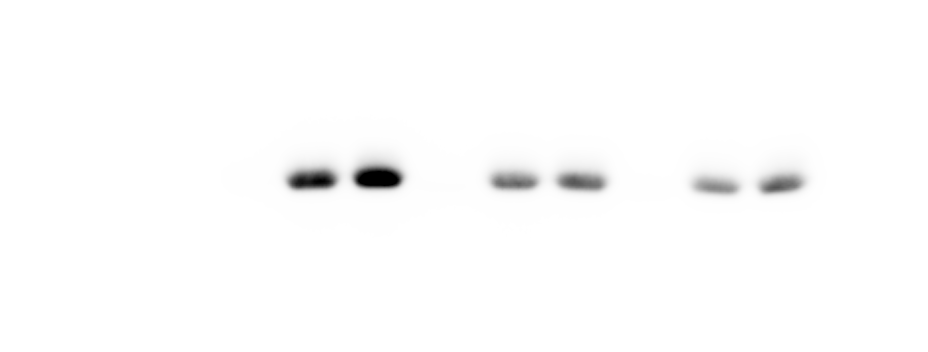

Supplement: Supplementary file 4 — Source data Fig. 1 [file 44318_2024_323_MOESM4_ESM.zip › SD figure 1/1D/western-pCHK1S345.tif]

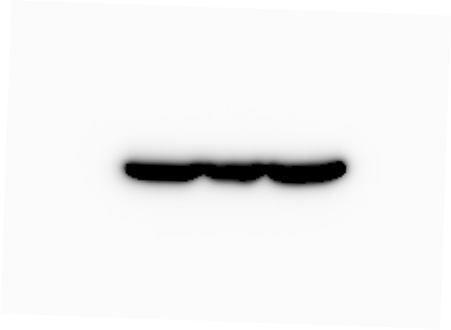

Supplement: Supplementary file 4 — Source data Fig. 1 [file 44318_2024_323_MOESM4_ESM.zip › SD figure 1/1E/western Actin.tif]

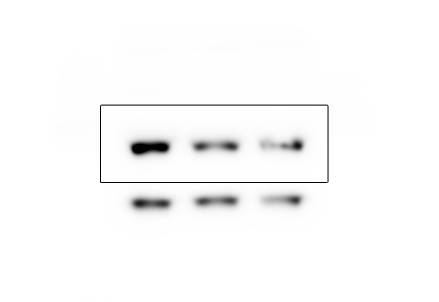

Supplement: Supplementary file 4 — Source data Fig. 1 [file 44318_2024_323_MOESM4_ESM.zip › SD figure 1/1E/western FOXP1.tif]

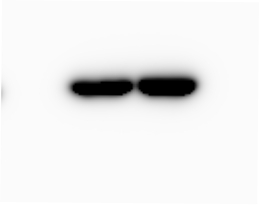

Supplement: Supplementary file 4 — Source data Fig. 1 [file 44318_2024_323_MOESM4_ESM.zip › SD figure 1/1G/western Actin.tif]

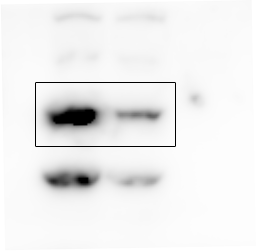

Supplement: Supplementary file 4 — Source data Fig. 1 [file 44318_2024_323_MOESM4_ESM.zip › SD figure 1/1G/western FOXP1.tif]

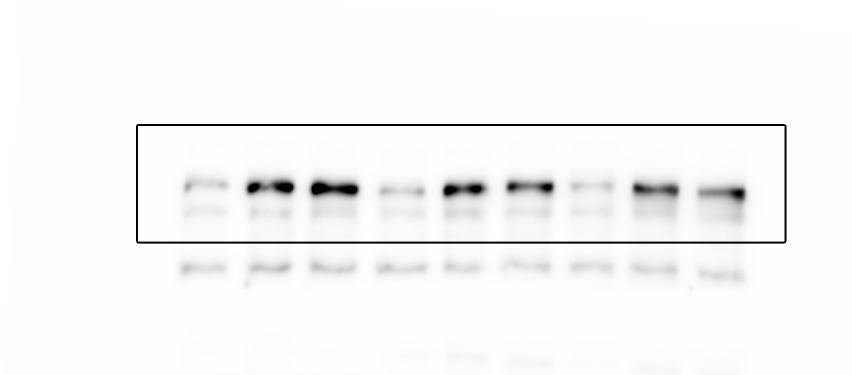

Supplement: Supplementary file 4 — Source data Fig. 1 [file 44318_2024_323_MOESM4_ESM.zip › SD figure 1/1H/western ATR CF.tif]

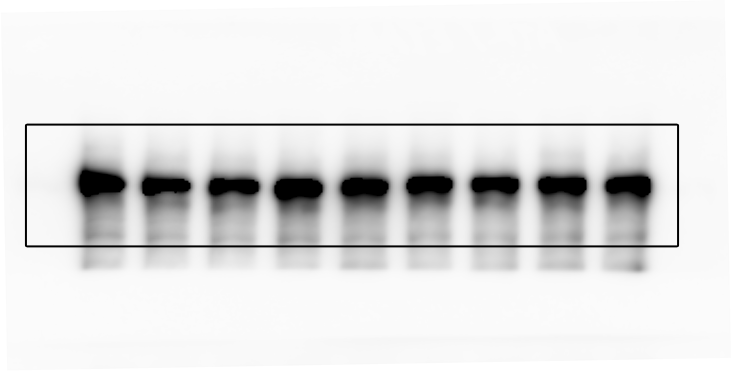

Supplement: Supplementary file 4 — Source data Fig. 1 [file 44318_2024_323_MOESM4_ESM.zip › SD figure 1/1H/western ATR WCL.tif]

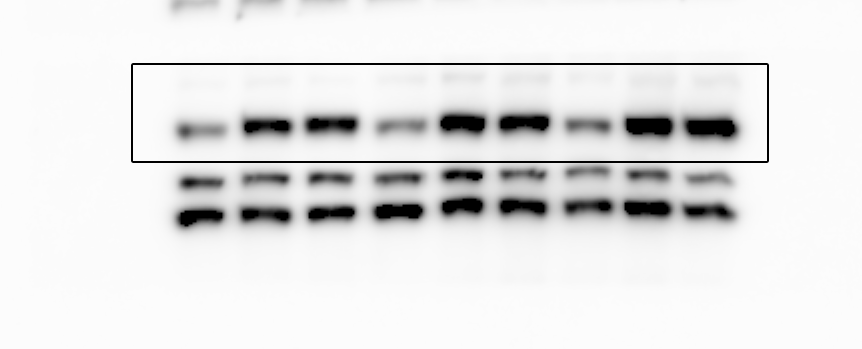

Supplement: Supplementary file 4 — Source data Fig. 1 [file 44318_2024_323_MOESM4_ESM.zip › SD figure 1/1H/western ETAA1 CF.tif]

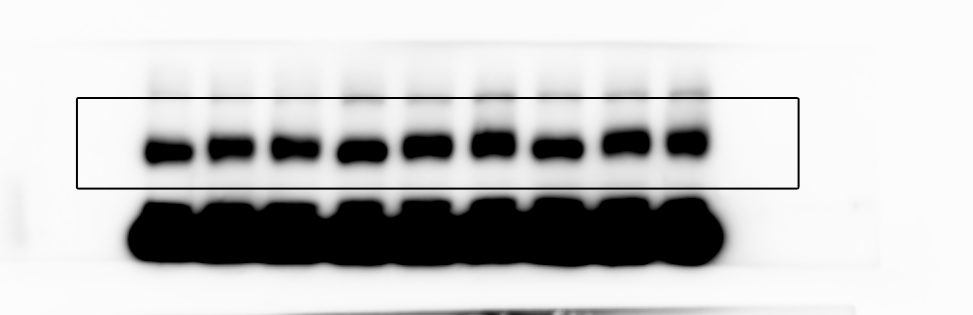

Supplement: Supplementary file 4 — Source data Fig. 1 [file 44318_2024_323_MOESM4_ESM.zip › SD figure 1/1H/western ETAA1 WCL.tif]

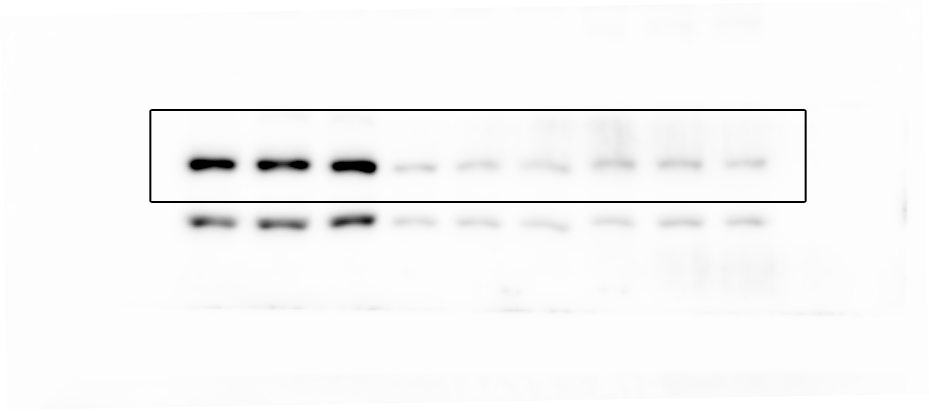

Supplement: Supplementary file 4 — Source data Fig. 1 [file 44318_2024_323_MOESM4_ESM.zip › SD figure 1/1H/western FOXP1 WCL.tif]

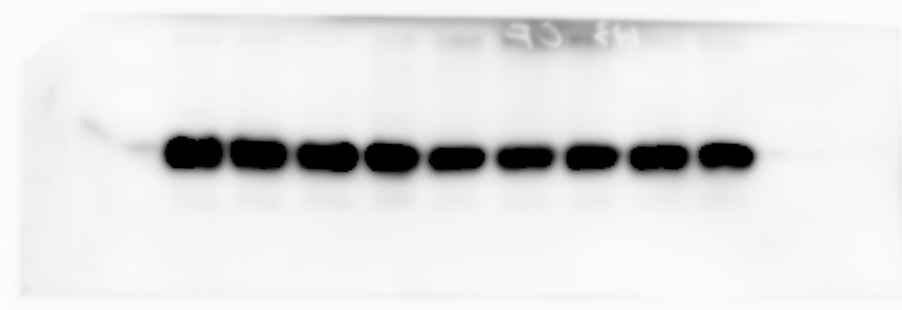

Supplement: Supplementary file 4 — Source data Fig. 1 [file 44318_2024_323_MOESM4_ESM.zip › SD figure 1/1H/western H3 CF.tif]

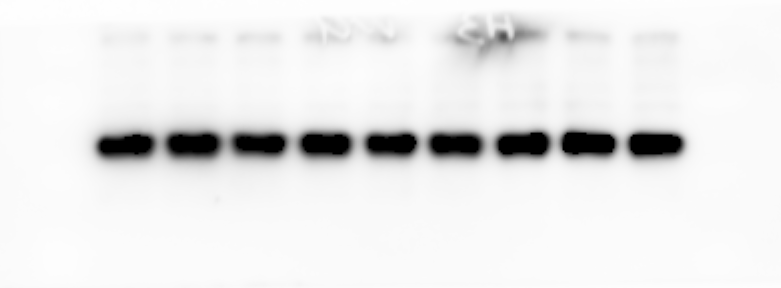

Supplement: Supplementary file 4 — Source data Fig. 1 [file 44318_2024_323_MOESM4_ESM.zip › SD figure 1/1H/western H3 WCL.tif]

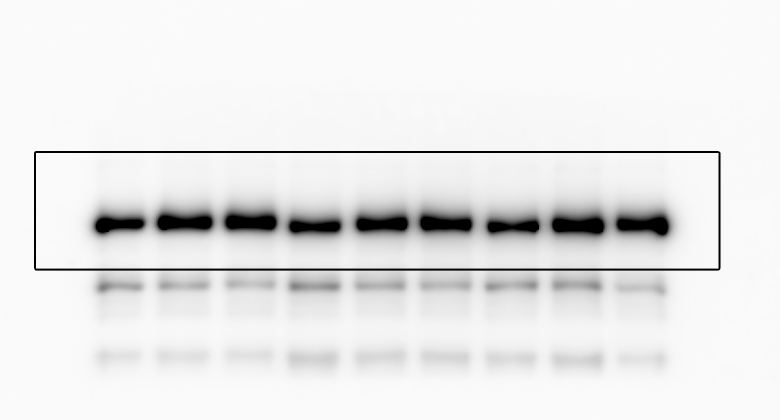

Supplement: Supplementary file 4 — Source data Fig. 1 [file 44318_2024_323_MOESM4_ESM.zip › SD figure 1/1H/western TopBP1 CF.tif]

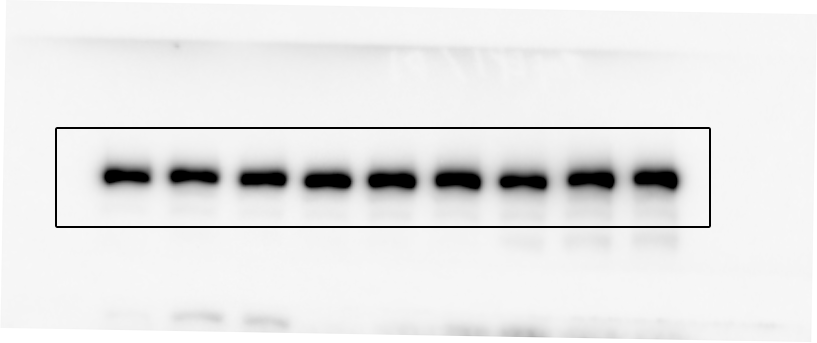

Supplement: Supplementary file 4 — Source data Fig. 1 [file 44318_2024_323_MOESM4_ESM.zip › SD figure 1/1H/western TopBP1 WCL.tif]

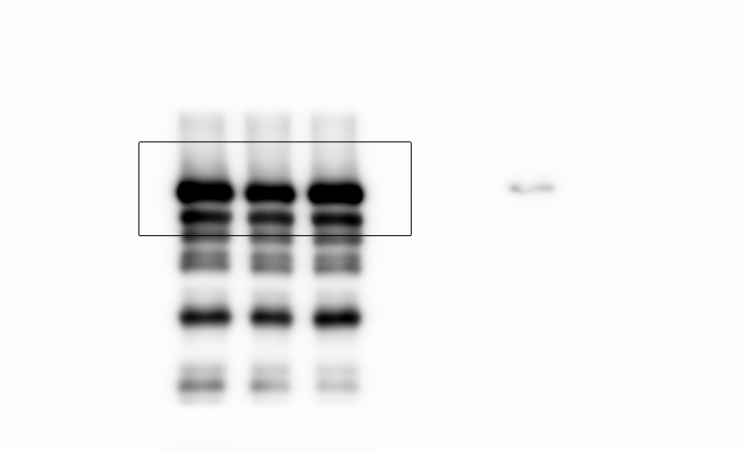

Supplement: Supplementary file 5 — Source data Fig. 2 [file 44318_2024_323_MOESM5_ESM.zip › SD figure 2/2A/western FOXP1 INPUT.tif]

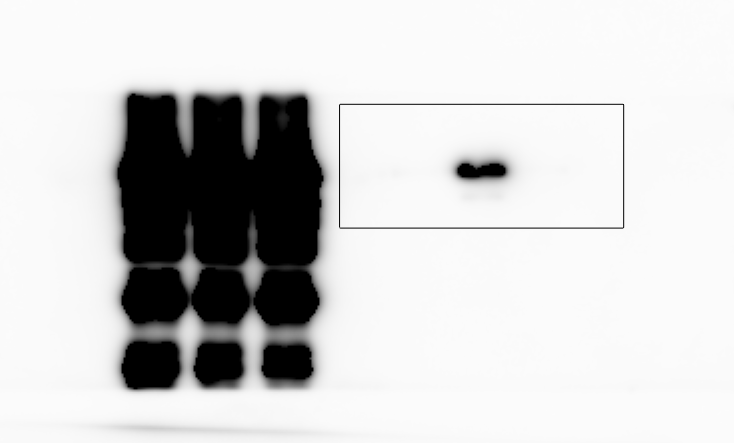

Supplement: Supplementary file 5 — Source data Fig. 2 [file 44318_2024_323_MOESM5_ESM.zip › SD figure 2/2A/western FOXP1 pulldown.tif]

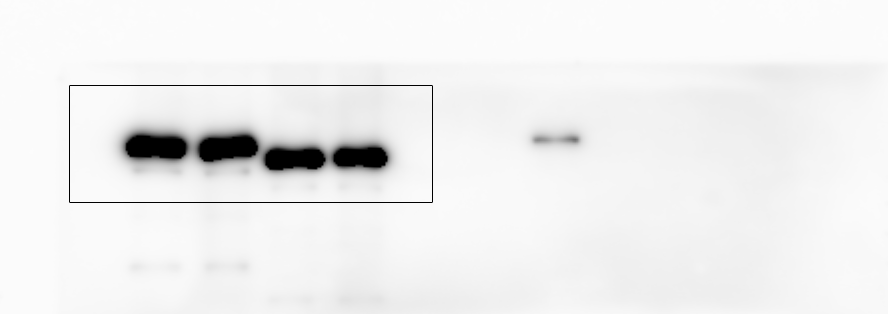

Supplement: Supplementary file 5 — Source data Fig. 2 [file 44318_2024_323_MOESM5_ESM.zip › SD figure 2/2B/western FOXP1 INPUT.tif]

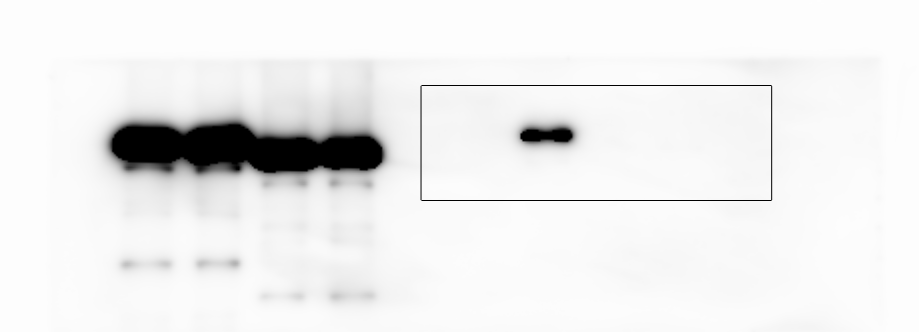

Supplement: Supplementary file 5 — Source data Fig. 2 [file 44318_2024_323_MOESM5_ESM.zip › SD figure 2/2B/western FOXP1 pulldown.tif]

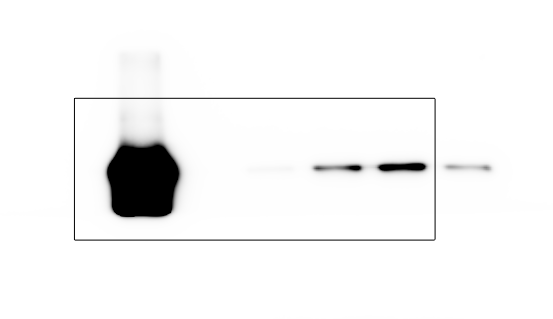

Supplement: Supplementary file 5 — Source data Fig. 2 [file 44318_2024_323_MOESM5_ESM.zip › SD figure 2/2C/western FOXP1.tif]

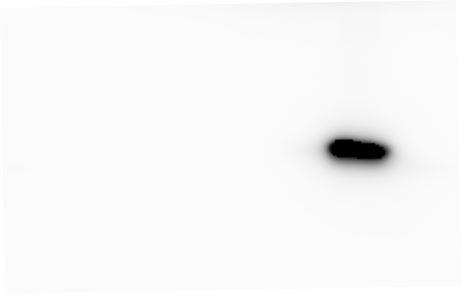

Supplement: Supplementary file 5 — Source data Fig. 2 [file 44318_2024_323_MOESM5_ESM.zip › SD figure 2/2C/western RPA32.tif]

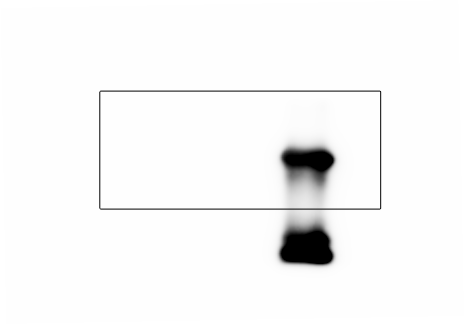

Supplement: Supplementary file 5 — Source data Fig. 2 [file 44318_2024_323_MOESM5_ESM.zip › SD figure 2/2C/western RPA70.tif]

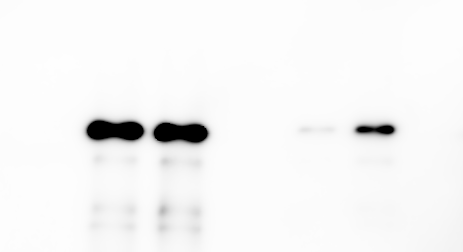

Supplement: Supplementary file 5 — Source data Fig. 2 [file 44318_2024_323_MOESM5_ESM.zip › SD figure 2/2D/western GST.tif]

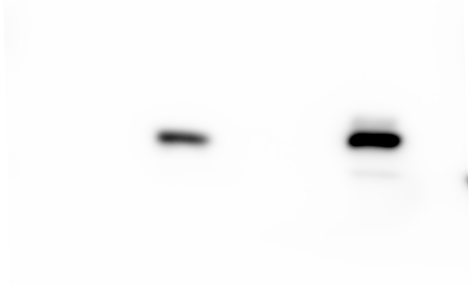

Supplement: Supplementary file 5 — Source data Fig. 2 [file 44318_2024_323_MOESM5_ESM.zip › SD figure 2/2D/western His.tif]

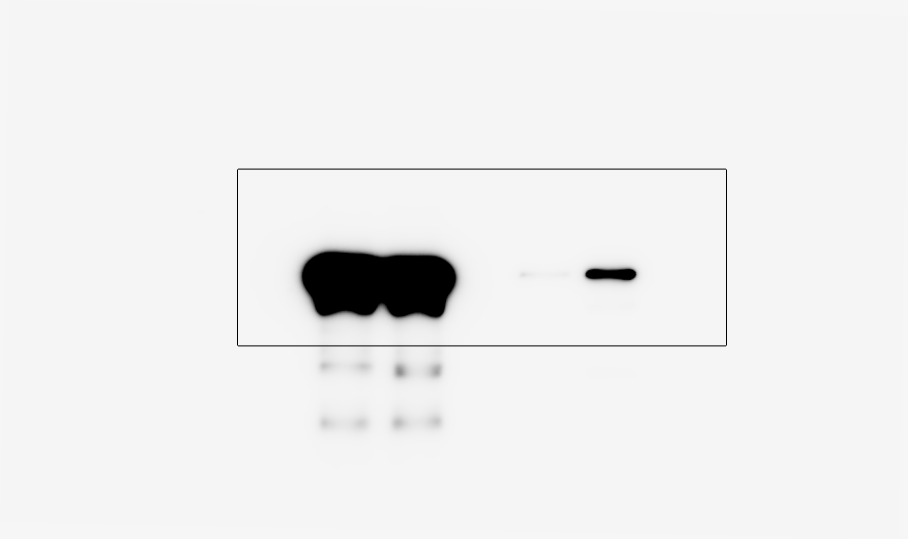

Supplement: Supplementary file 5 — Source data Fig. 2 [file 44318_2024_323_MOESM5_ESM.zip › SD figure 2/2E/western GST.tif]

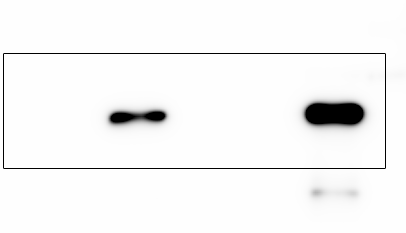

Supplement: Supplementary file 5 — Source data Fig. 2 [file 44318_2024_323_MOESM5_ESM.zip › SD figure 2/2E/western His.tif]

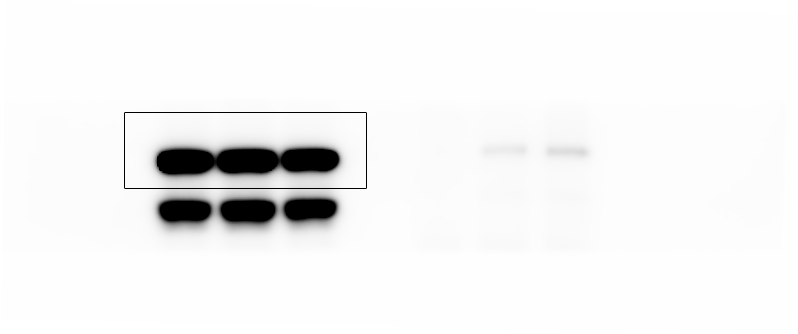

Supplement: Supplementary file 5 — Source data Fig. 2 [file 44318_2024_323_MOESM5_ESM.zip › SD figure 2/2F/western FOXP1 INPUT.tif]

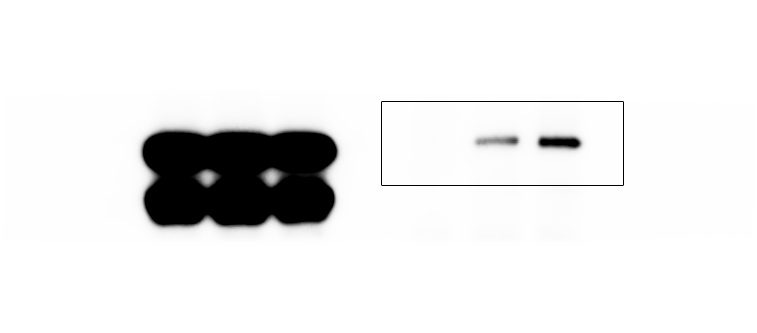

Supplement: Supplementary file 5 — Source data Fig. 2 [file 44318_2024_323_MOESM5_ESM.zip › SD figure 2/2F/western FOXP1 IP.tif]

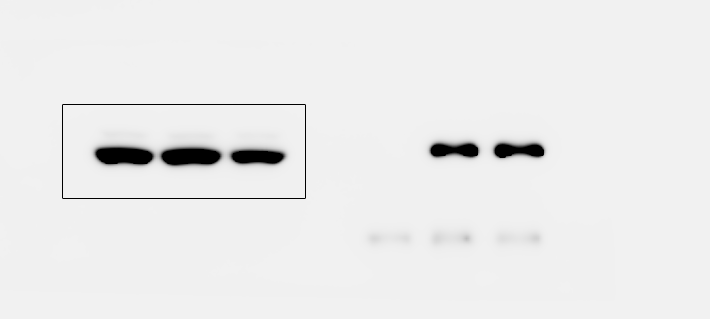

Supplement: Supplementary file 5 — Source data Fig. 2 [file 44318_2024_323_MOESM5_ESM.zip › SD figure 2/2F/western RPA70 INPUT.tif]

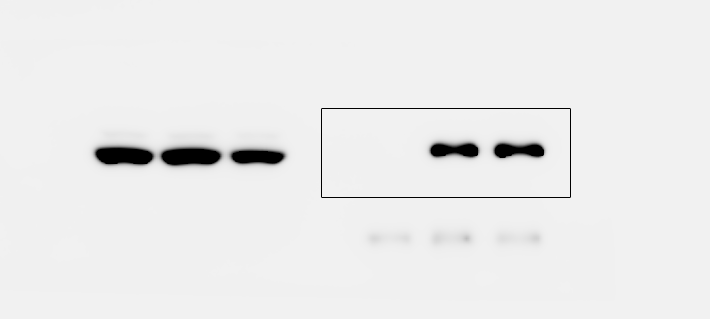

Supplement: Supplementary file 5 — Source data Fig. 2 [file 44318_2024_323_MOESM5_ESM.zip › SD figure 2/2F/western RPA70 IP.tif]

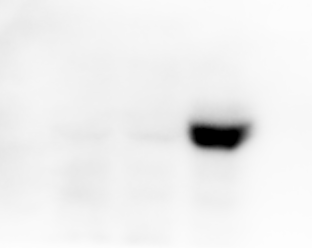

Supplement: Supplementary file 5 — Source data Fig. 2 [file 44318_2024_323_MOESM5_ESM.zip › SD figure 2/2F/western pCHK1S345.tif]

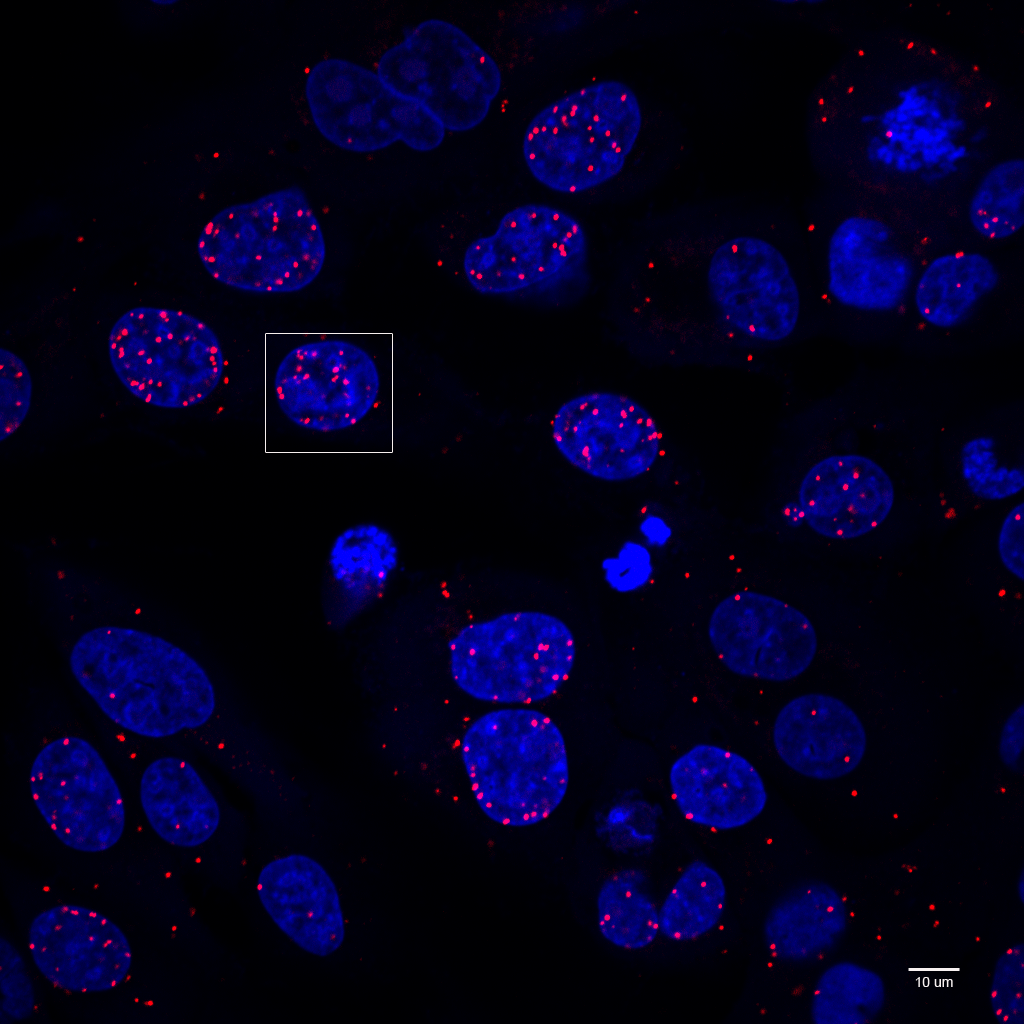

Supplement: Supplementary file 5 — Source data Fig. 2 [file 44318_2024_323_MOESM5_ESM.zip › SD figure 2/2G/image Biotin-FOXP1 HU 1h.tif]

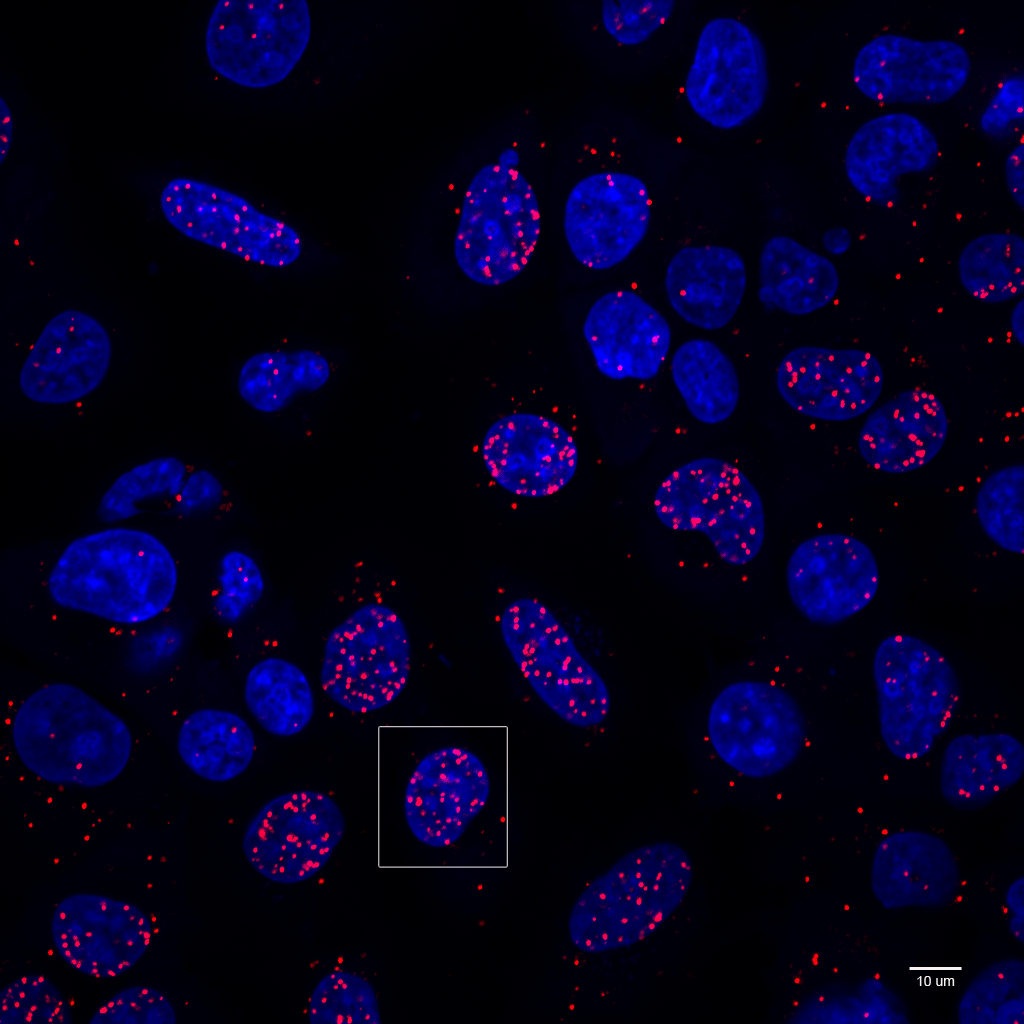

Supplement: Supplementary file 5 — Source data Fig. 2 [file 44318_2024_323_MOESM5_ESM.zip › SD figure 2/2G/image Biotin-FOXP1 HU 2h.tif]

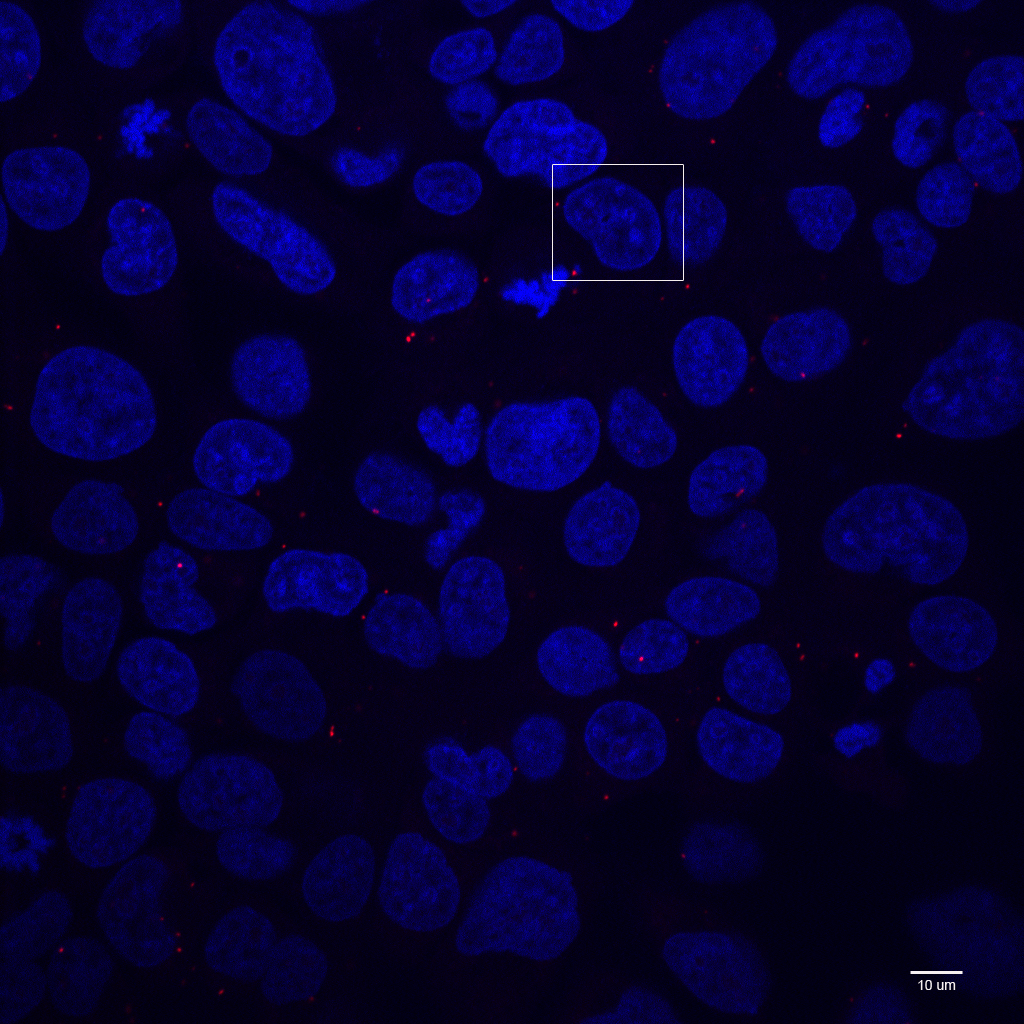

Supplement: Supplementary file 5 — Source data Fig. 2 [file 44318_2024_323_MOESM5_ESM.zip › SD figure 2/2G/image Biotin-FOXP1 no click.tif]

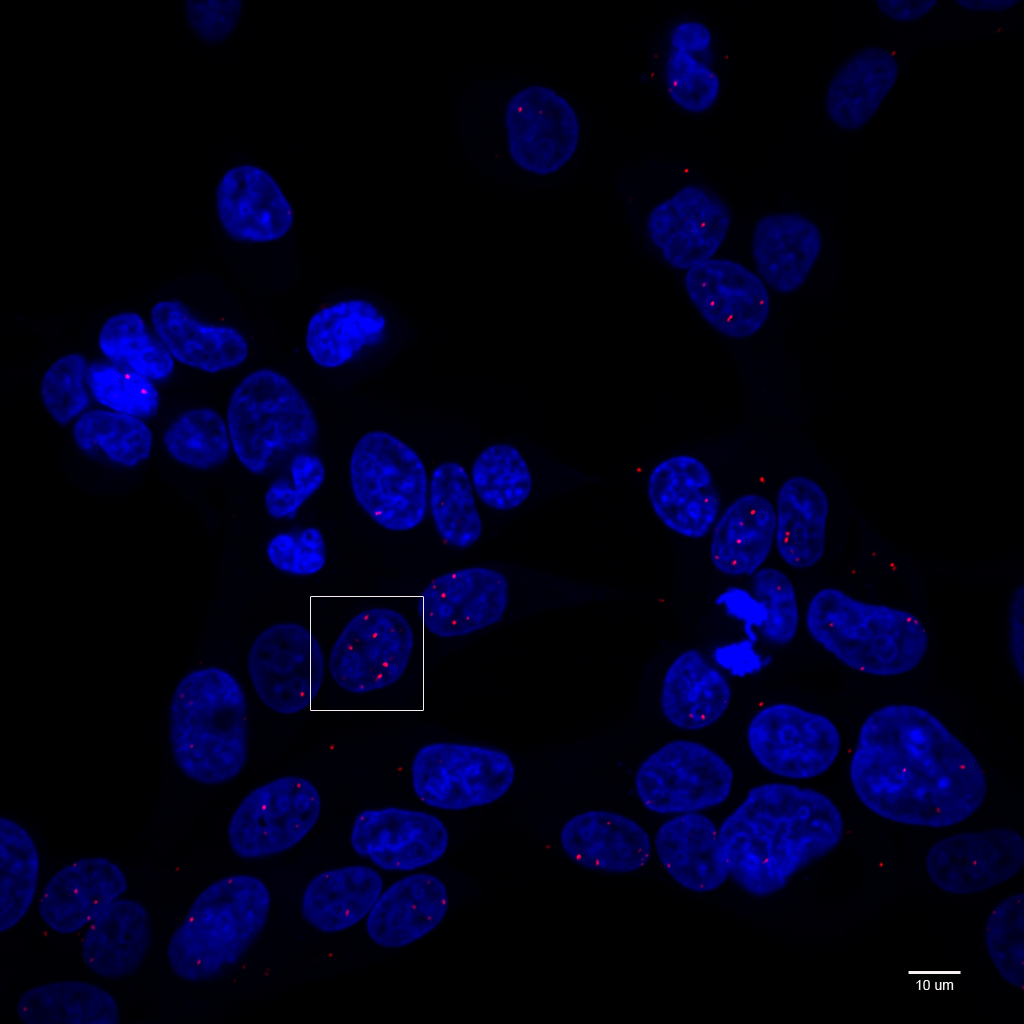

Supplement: Supplementary file 5 — Source data Fig. 2 [file 44318_2024_323_MOESM5_ESM.zip › SD figure 2/2G/image Biotin-FOXP1 no treatment.tif]

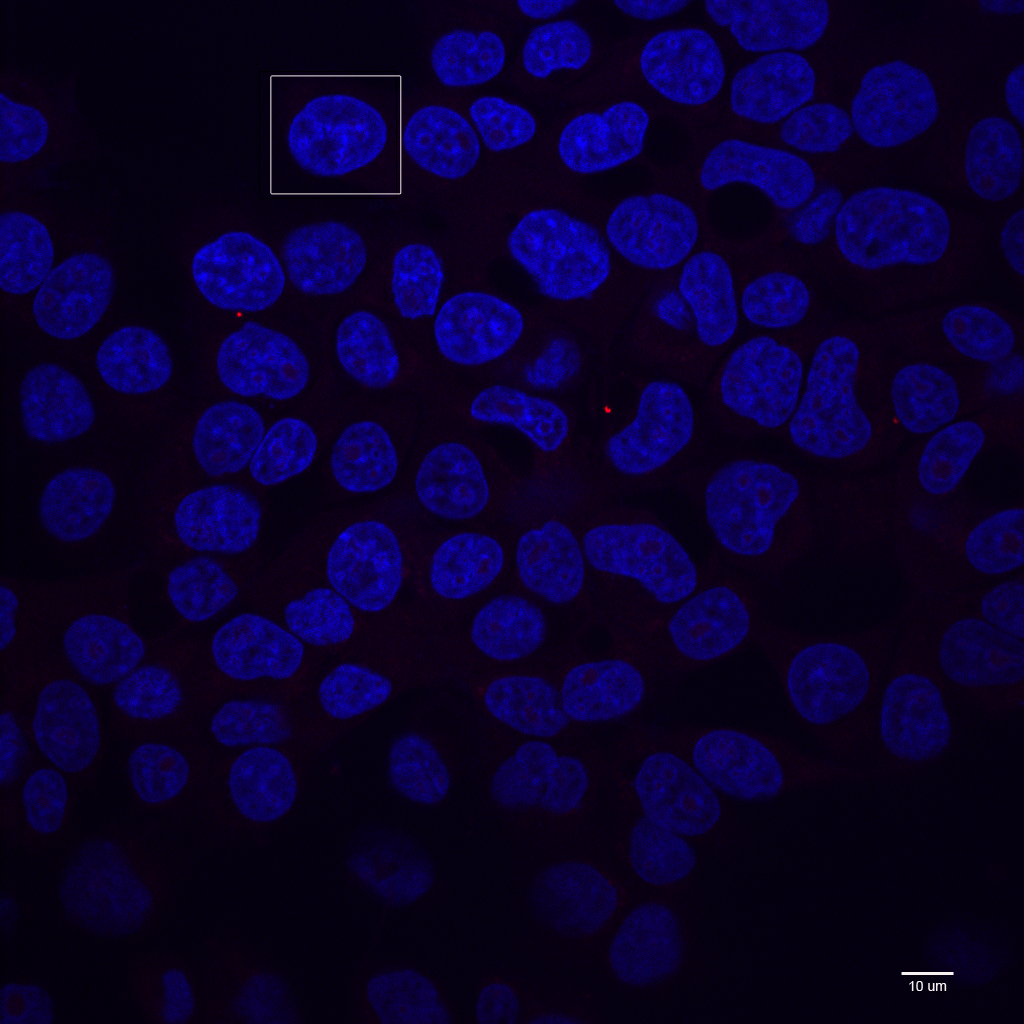

Supplement: Supplementary file 5 — Source data Fig. 2 [file 44318_2024_323_MOESM5_ESM.zip › SD figure 2/2G/image Biotin.tif]

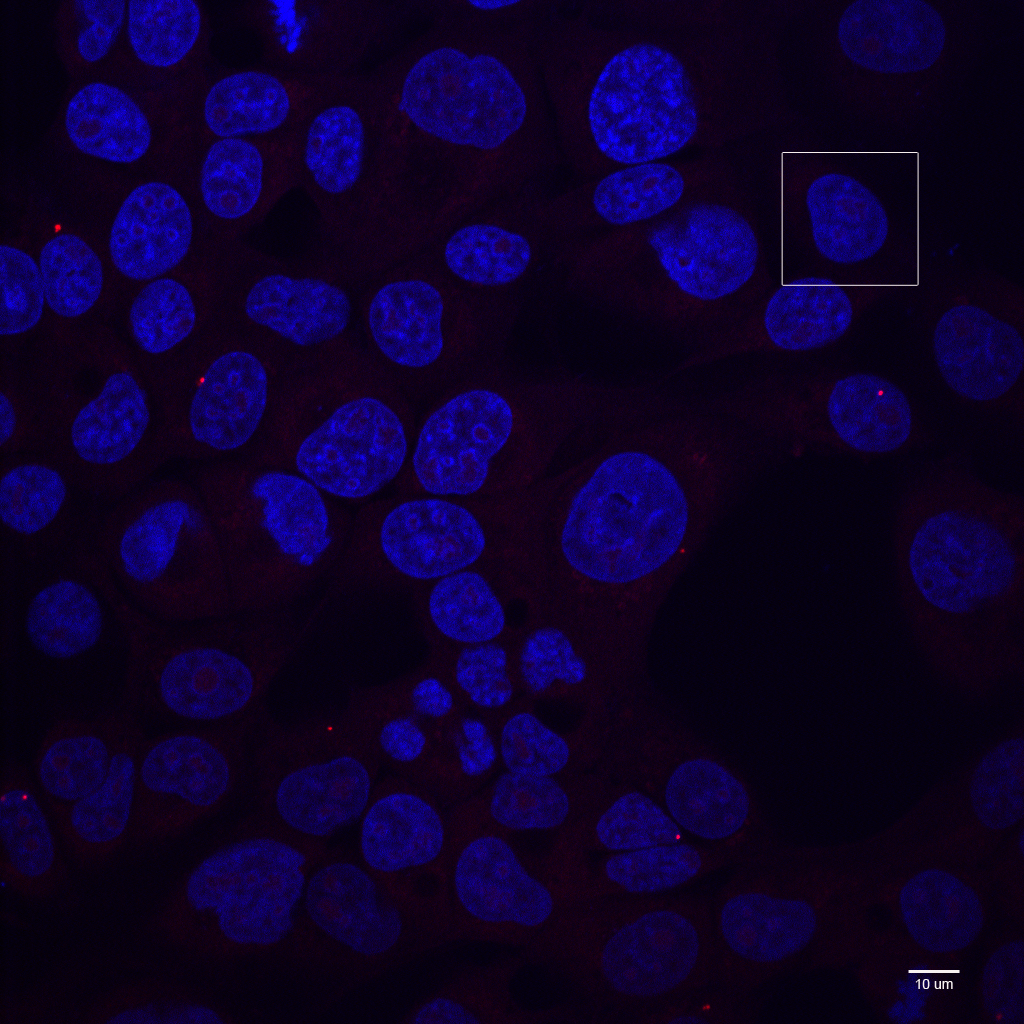

Supplement: Supplementary file 5 — Source data Fig. 2 [file 44318_2024_323_MOESM5_ESM.zip › SD figure 2/2G/image FOXP1.tif]

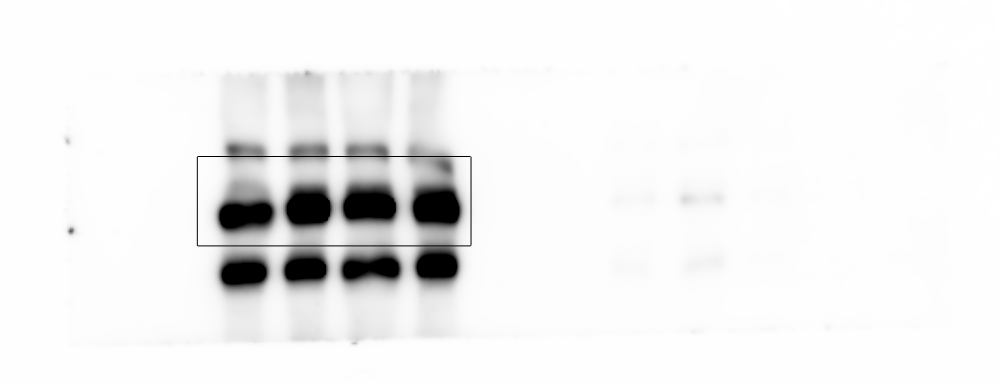

Supplement: Supplementary file 5 — Source data Fig. 2 [file 44318_2024_323_MOESM5_ESM.zip › SD figure 2/2H/western FOXP1 INPUT.tif]

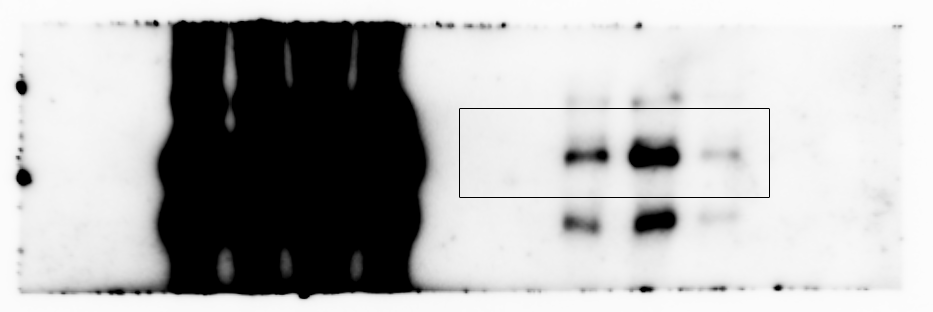

Supplement: Supplementary file 5 — Source data Fig. 2 [file 44318_2024_323_MOESM5_ESM.zip › SD figure 2/2H/western FOXP1 pulldown.tif]

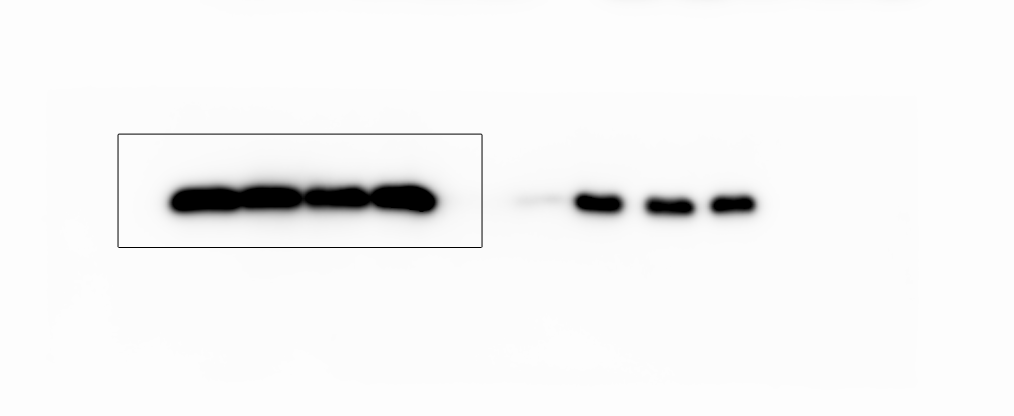

Supplement: Supplementary file 5 — Source data Fig. 2 [file 44318_2024_323_MOESM5_ESM.zip › SD figure 2/2H/western H3 INPUT.tif]

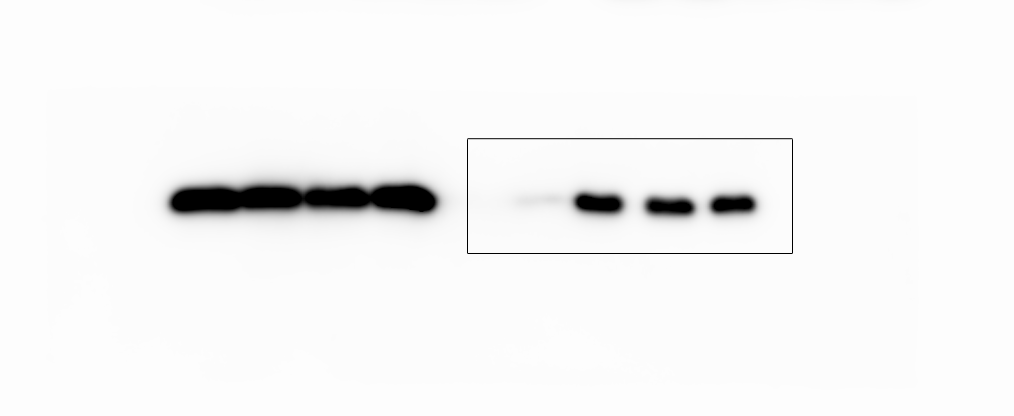

Supplement: Supplementary file 5 — Source data Fig. 2 [file 44318_2024_323_MOESM5_ESM.zip › SD figure 2/2H/western H3 pulldown.tif]

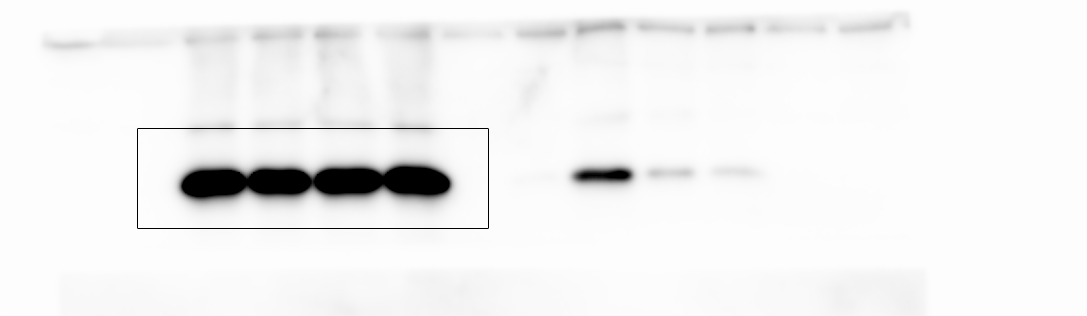

Supplement: Supplementary file 5 — Source data Fig. 2 [file 44318_2024_323_MOESM5_ESM.zip › SD figure 2/2H/western PCNA INPUT.tif]

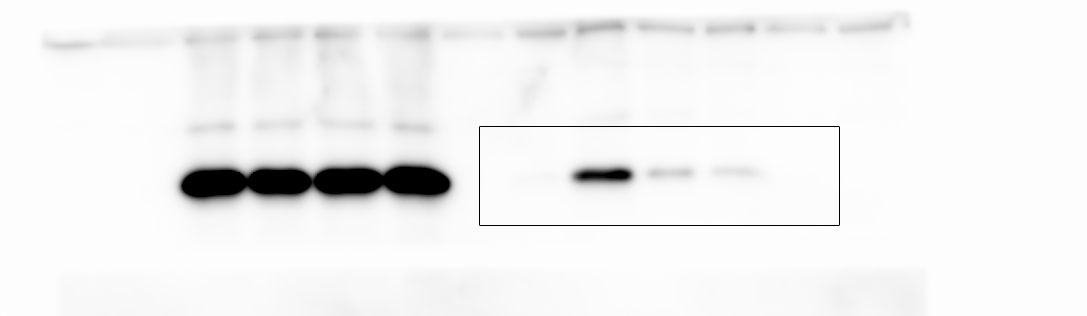

Supplement: Supplementary file 5 — Source data Fig. 2 [file 44318_2024_323_MOESM5_ESM.zip › SD figure 2/2H/western PCNA pulldown.tif]

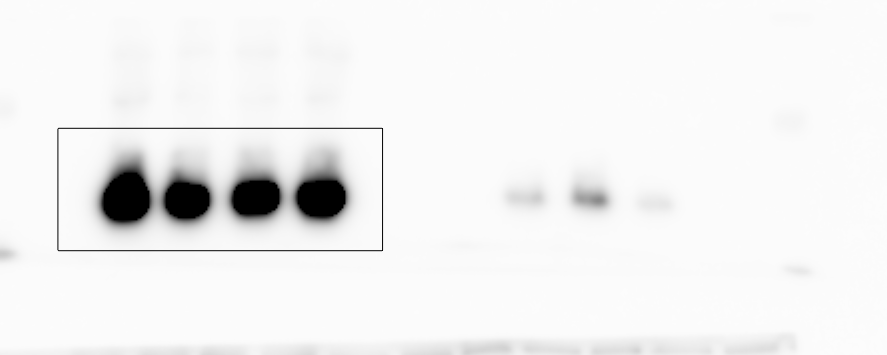

Supplement: Supplementary file 5 — Source data Fig. 2 [file 44318_2024_323_MOESM5_ESM.zip › SD figure 2/2H/western RPA32 INPUT.tif]

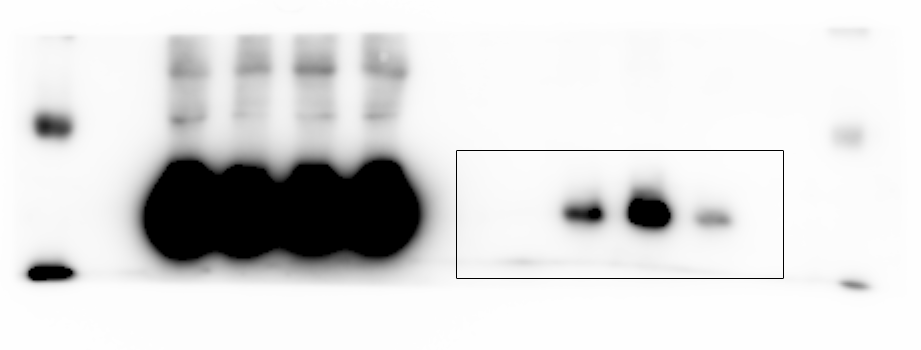

Supplement: Supplementary file 5 — Source data Fig. 2 [file 44318_2024_323_MOESM5_ESM.zip › SD figure 2/2H/western RPA32 pulldown.tif]

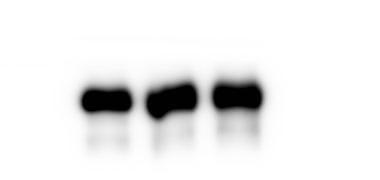

Supplement: Supplementary file 6 — Source data Fig. 3 [file 44318_2024_323_MOESM6_ESM.zip › SD figure 3/3A/western FLAG.tif]

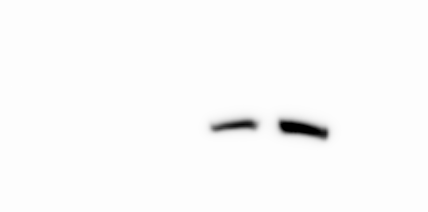

Supplement: Supplementary file 6 — Source data Fig. 3 [file 44318_2024_323_MOESM6_ESM.zip › SD figure 3/3A/western GFP.tif]

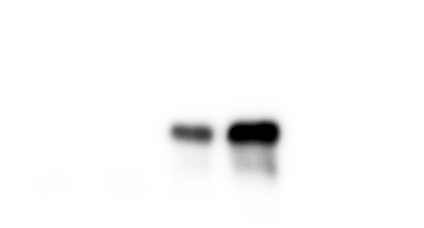

Supplement: Supplementary file 6 — Source data Fig. 3 [file 44318_2024_323_MOESM6_ESM.zip › SD figure 3/3A/western O-GlcNAc.tif]

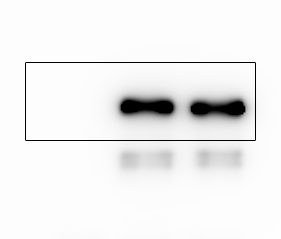

Supplement: Supplementary file 6 — Source data Fig. 3 [file 44318_2024_323_MOESM6_ESM.zip › SD figure 3/3B/western FOXP1.tif]

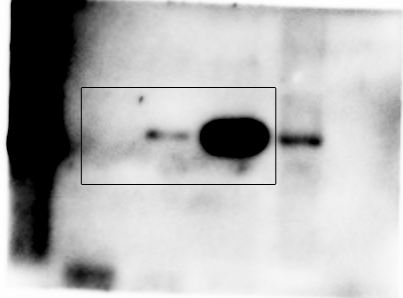

Supplement: Supplementary file 6 — Source data Fig. 3 [file 44318_2024_323_MOESM6_ESM.zip › SD figure 3/3B/western O-GlcNAc LE.tif]

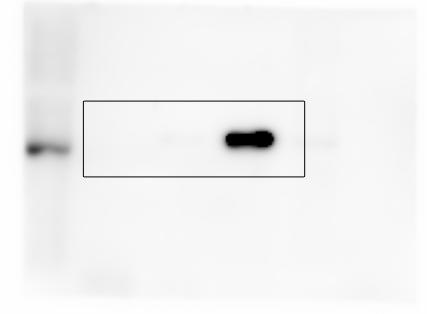

Supplement: Supplementary file 6 — Source data Fig. 3 [file 44318_2024_323_MOESM6_ESM.zip › SD figure 3/3B/western O-GlcNAc SE.tif]

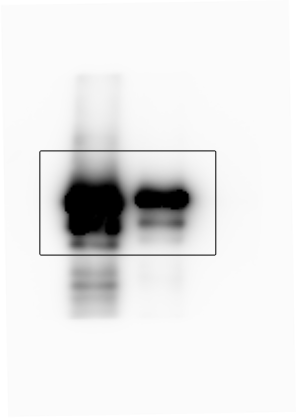

Supplement: Supplementary file 6 — Source data Fig. 3 [file 44318_2024_323_MOESM6_ESM.zip › SD figure 3/3C/western FOXP1.tif]

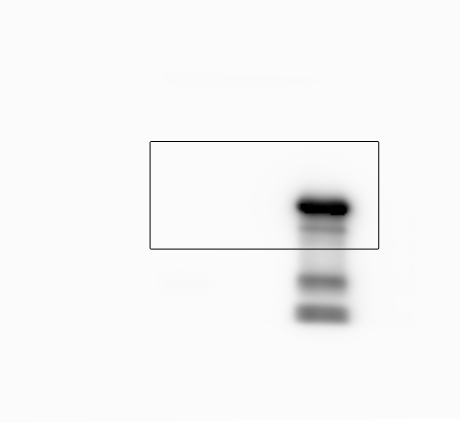

Supplement: Supplementary file 6 — Source data Fig. 3 [file 44318_2024_323_MOESM6_ESM.zip › SD figure 3/3C/western O-GlcNAc.tif]

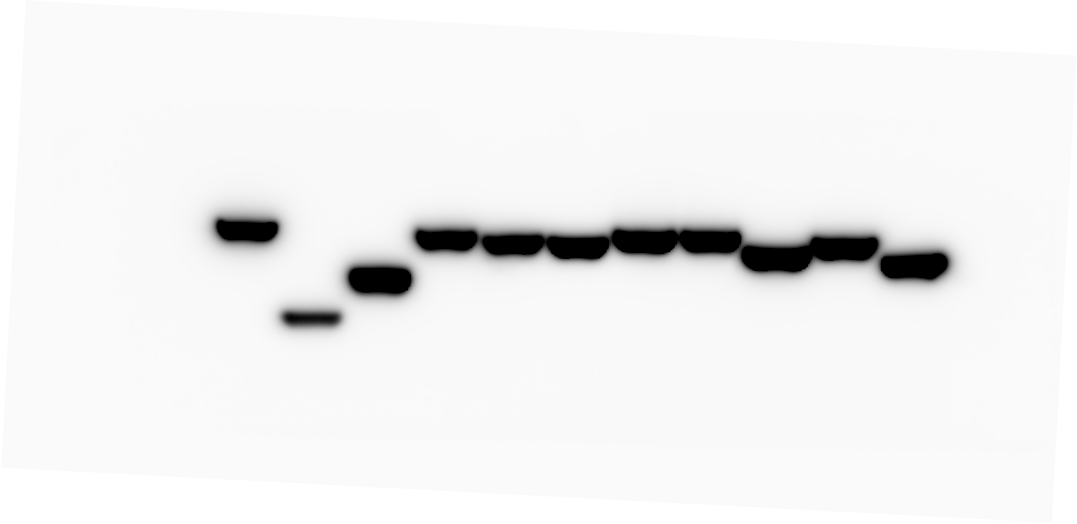

Supplement: Supplementary file 6 — Source data Fig. 3 [file 44318_2024_323_MOESM6_ESM.zip › SD figure 3/3D/western FLAG-INPUT.tif]

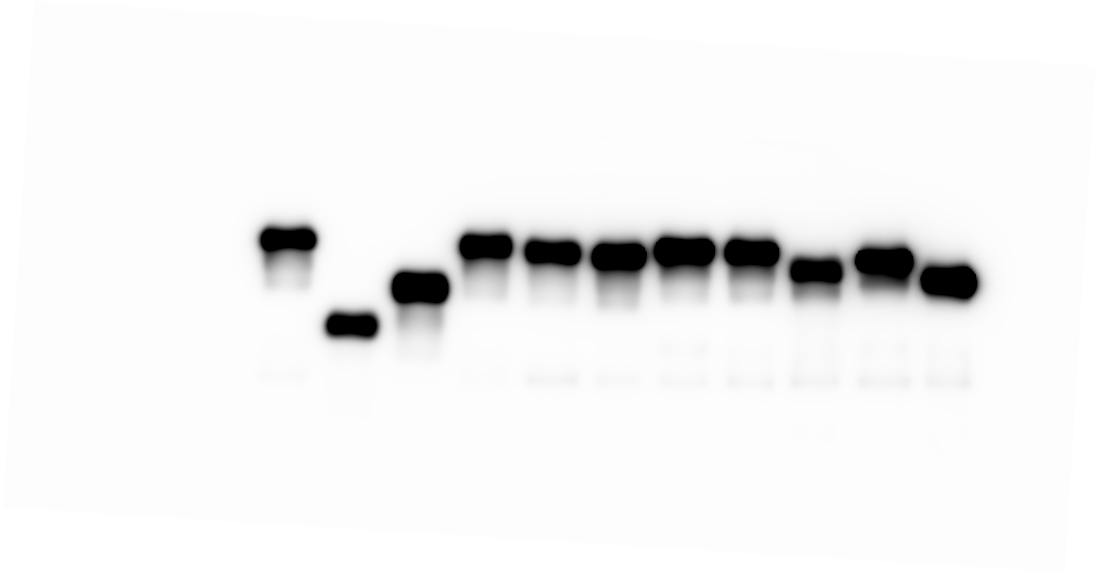

Supplement: Supplementary file 6 — Source data Fig. 3 [file 44318_2024_323_MOESM6_ESM.zip › SD figure 3/3D/western FLAG-IP.tif]

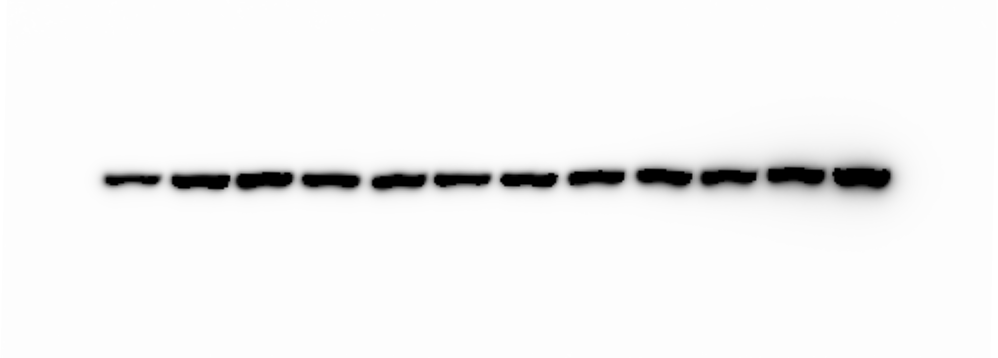

Supplement: Supplementary file 6 — Source data Fig. 3 [file 44318_2024_323_MOESM6_ESM.zip › SD figure 3/3D/western GFP-INPUT.tif]

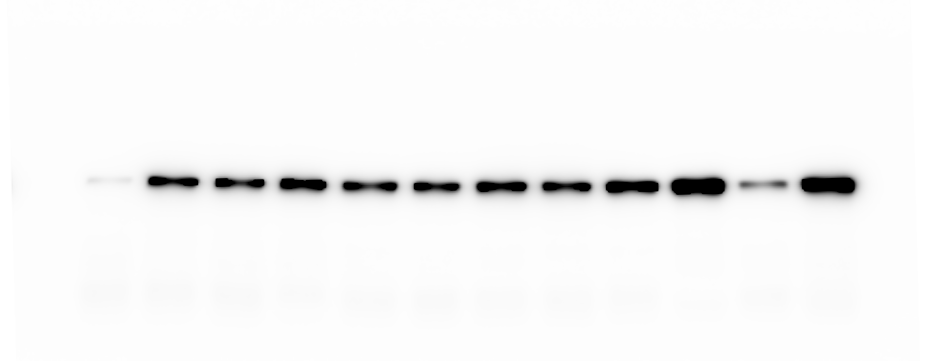

Supplement: Supplementary file 6 — Source data Fig. 3 [file 44318_2024_323_MOESM6_ESM.zip › SD figure 3/3D/western GFP-IP.tif]

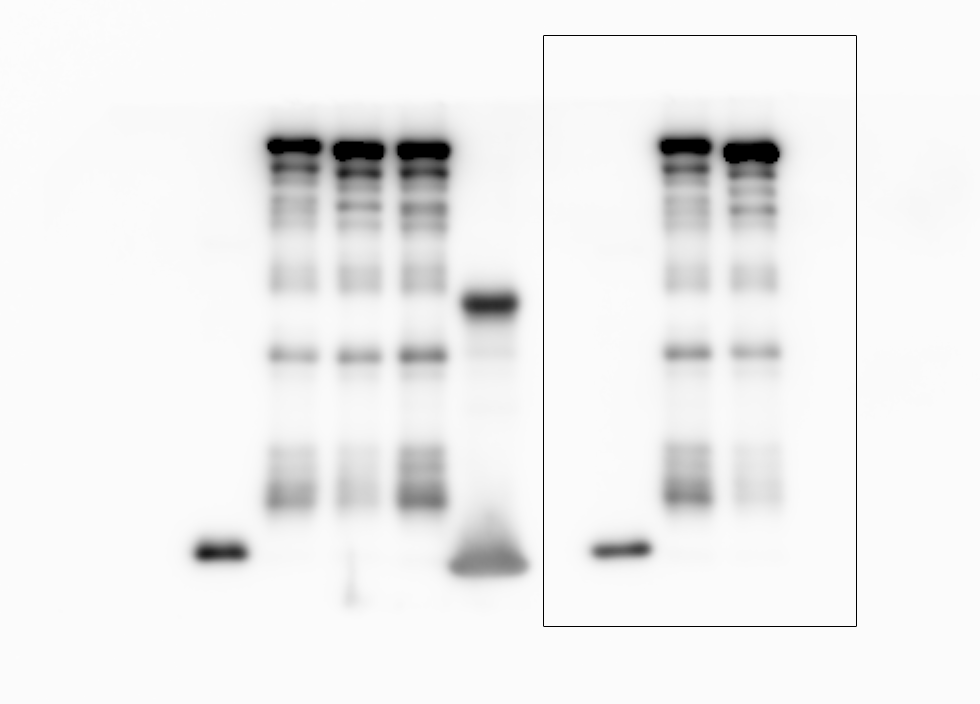

Supplement: Supplementary file 6 — Source data Fig. 3 [file 44318_2024_323_MOESM6_ESM.zip › SD figure 3/3E/western GST.tif]

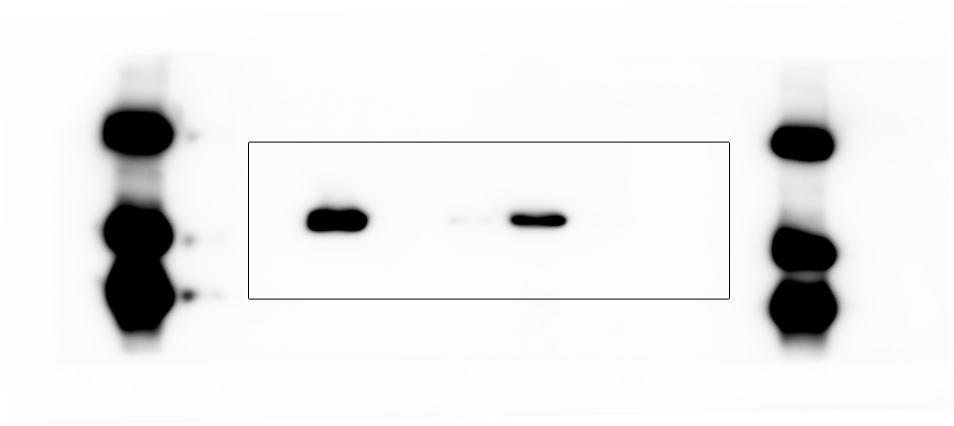

Supplement: Supplementary file 6 — Source data Fig. 3 [file 44318_2024_323_MOESM6_ESM.zip › SD figure 3/3E/western His.tif]

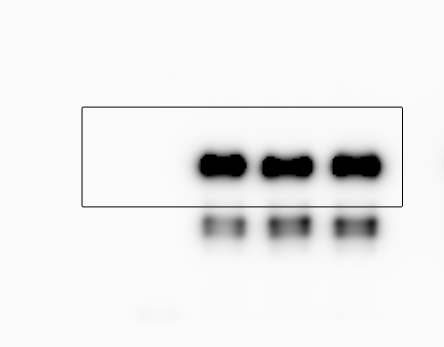

Supplement: Supplementary file 6 — Source data Fig. 3 [file 44318_2024_323_MOESM6_ESM.zip › SD figure 3/3F/western FOXP1.tif]

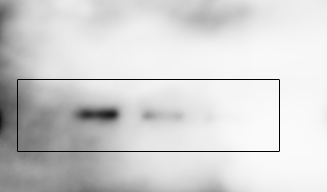

Supplement: Supplementary file 6 — Source data Fig. 3 [file 44318_2024_323_MOESM6_ESM.zip › SD figure 3/3F/western O-GlcNAc.tif]

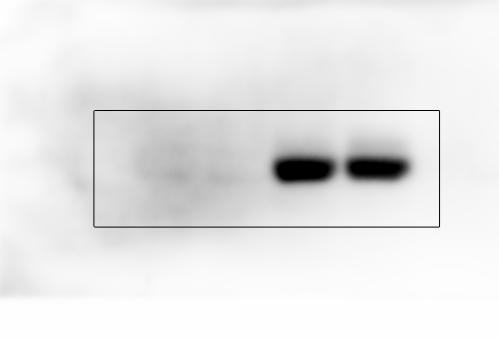

Supplement: Supplementary file 6 — Source data Fig. 3 [file 44318_2024_323_MOESM6_ESM.zip › SD figure 3/3F/western pCHK1S345.tif]

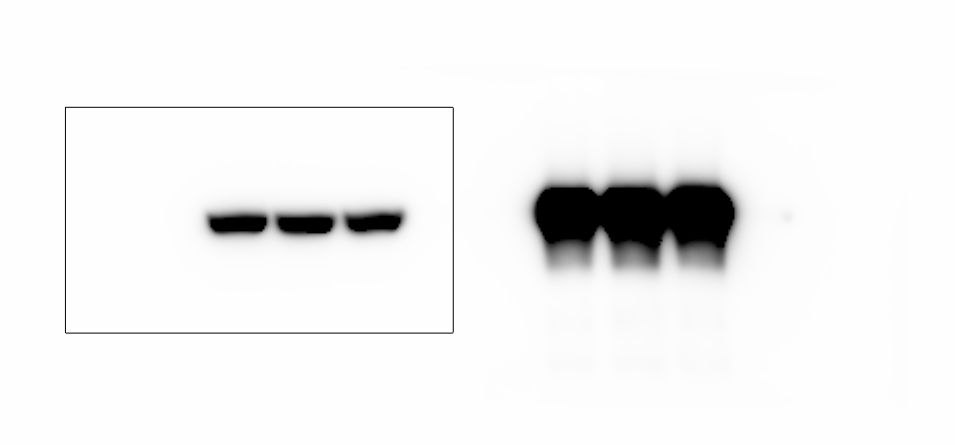

Supplement: Supplementary file 6 — Source data Fig. 3 [file 44318_2024_323_MOESM6_ESM.zip › SD figure 3/3G/western FLAG INPUT.tif]

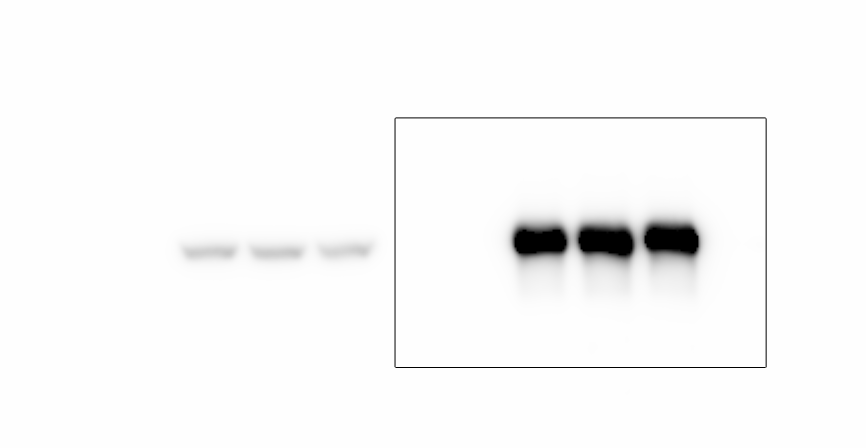

Supplement: Supplementary file 6 — Source data Fig. 3 [file 44318_2024_323_MOESM6_ESM.zip › SD figure 3/3G/western FLAG IP.tif]

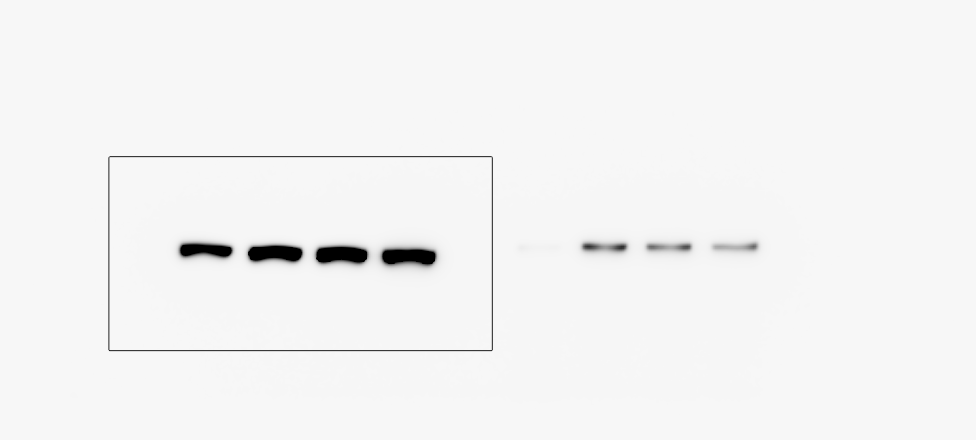

Supplement: Supplementary file 6 — Source data Fig. 3 [file 44318_2024_323_MOESM6_ESM.zip › SD figure 3/3G/western GFP INPUT.tif]

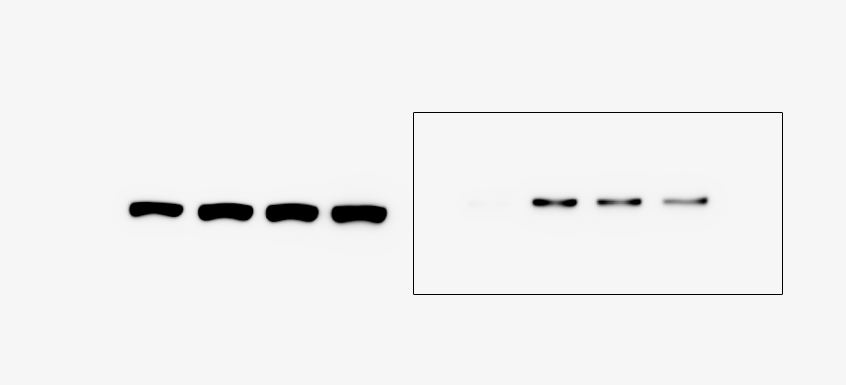

Supplement: Supplementary file 6 — Source data Fig. 3 [file 44318_2024_323_MOESM6_ESM.zip › SD figure 3/3G/western GFP IP.tif]

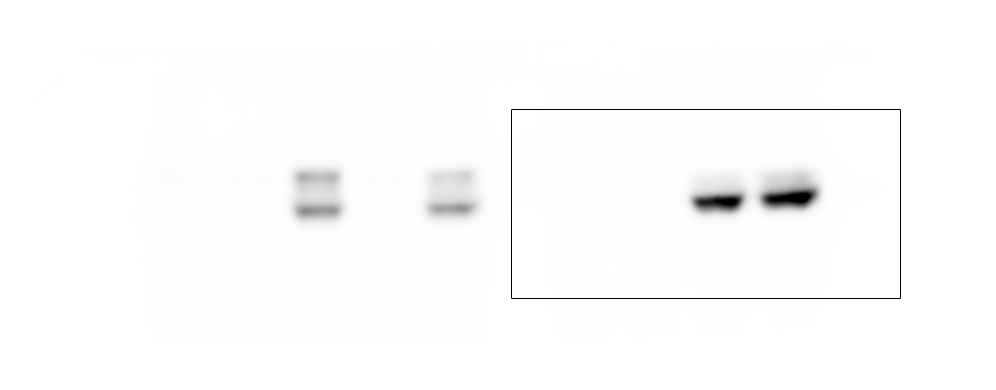

Supplement: Supplementary file 6 — Source data Fig. 3 [file 44318_2024_323_MOESM6_ESM.zip › SD figure 3/3G/western pCHK1S345 INPUT.tif]

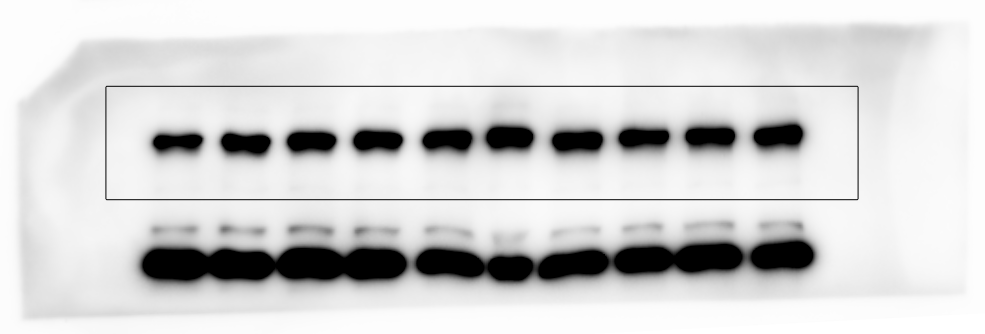

Supplement: Supplementary file 6 — Source data Fig. 3 [file 44318_2024_323_MOESM6_ESM.zip › SD figure 3/3H/western ATR INPUT.tif]

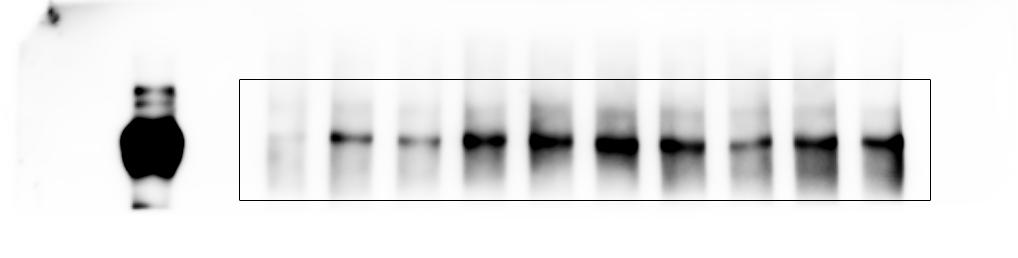

Supplement: Supplementary file 6 — Source data Fig. 3 [file 44318_2024_323_MOESM6_ESM.zip › SD figure 3/3H/western ATR IP.tif]

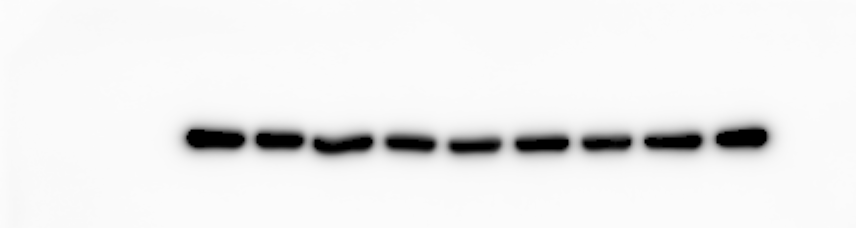

Supplement: Supplementary file 6 — Source data Fig. 3 [file 44318_2024_323_MOESM6_ESM.zip › SD figure 3/3H/western FLAG INPUT.tif]

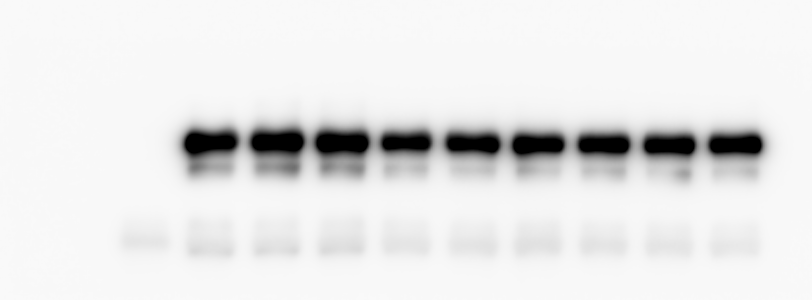

Supplement: Supplementary file 6 — Source data Fig. 3 [file 44318_2024_323_MOESM6_ESM.zip › SD figure 3/3H/western FLAG IP.tif]

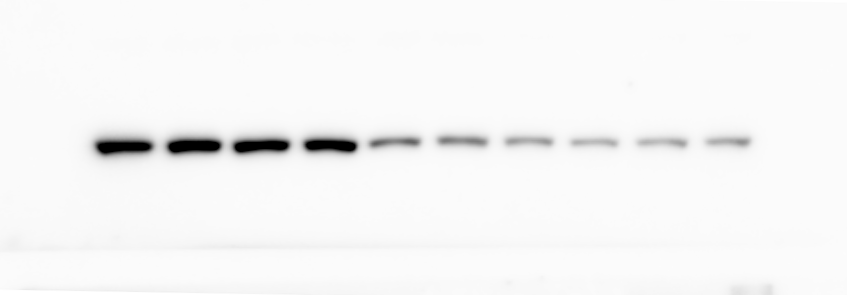

Supplement: Supplementary file 6 — Source data Fig. 3 [file 44318_2024_323_MOESM6_ESM.zip › SD figure 3/3H/western OGT.tif]

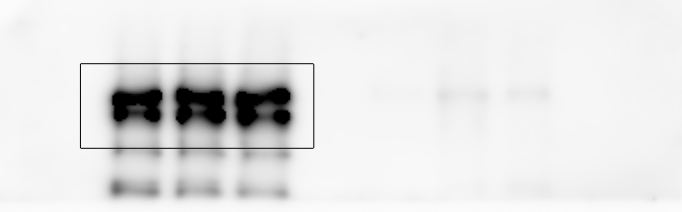

Supplement: Supplementary file 6 — Source data Fig. 3 [file 44318_2024_323_MOESM6_ESM.zip › SD figure 3/3I/western ATR INPUT.tif]

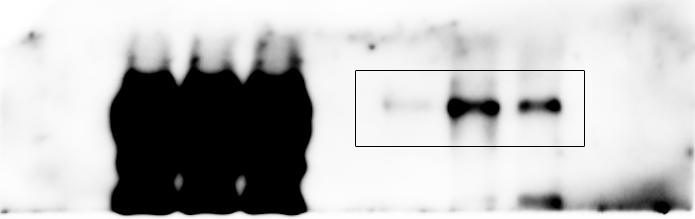

Supplement: Supplementary file 6 — Source data Fig. 3 [file 44318_2024_323_MOESM6_ESM.zip › SD figure 3/3I/western ATR pulldown.tif]

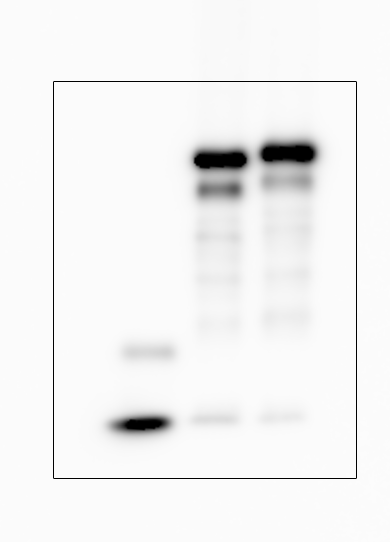

Supplement: Supplementary file 6 — Source data Fig. 3 [file 44318_2024_323_MOESM6_ESM.zip › SD figure 3/3I/western GST.tif]

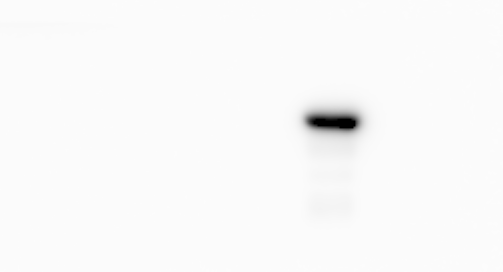

Supplement: Supplementary file 6 — Source data Fig. 3 [file 44318_2024_323_MOESM6_ESM.zip › SD figure 3/3I/western O-GlcNAc.tif]

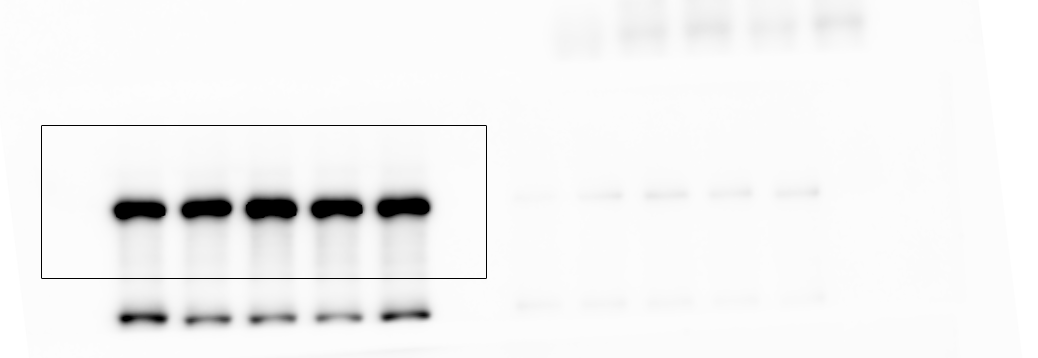

Supplement: Supplementary file 7 — Source data Fig. 4 [file 44318_2024_323_MOESM7_ESM.zip › SD figure 4/4A/western ATR-INPUT.tif]

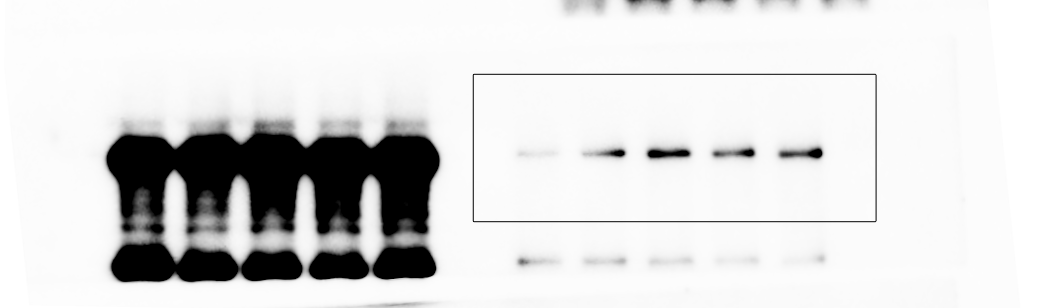

Supplement: Supplementary file 7 — Source data Fig. 4 [file 44318_2024_323_MOESM7_ESM.zip › SD figure 4/4A/western ATR-IP.tif]

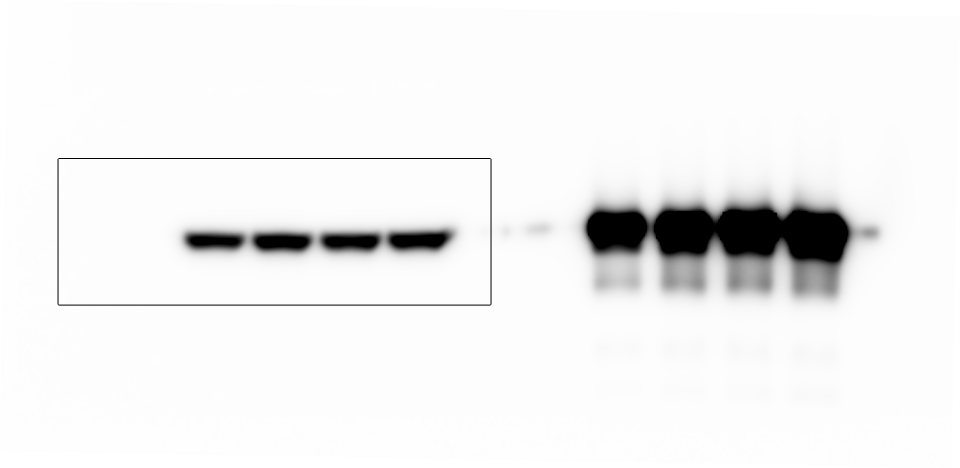

Supplement: Supplementary file 7 — Source data Fig. 4 [file 44318_2024_323_MOESM7_ESM.zip › SD figure 4/4A/western FLAG-INPUT.tif]

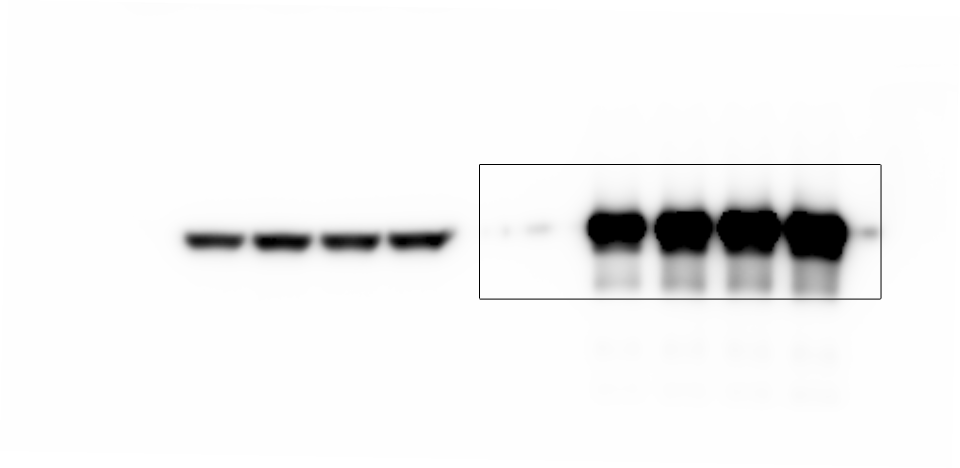

Supplement: Supplementary file 7 — Source data Fig. 4 [file 44318_2024_323_MOESM7_ESM.zip › SD figure 4/4A/western FLAG-IP.tif]

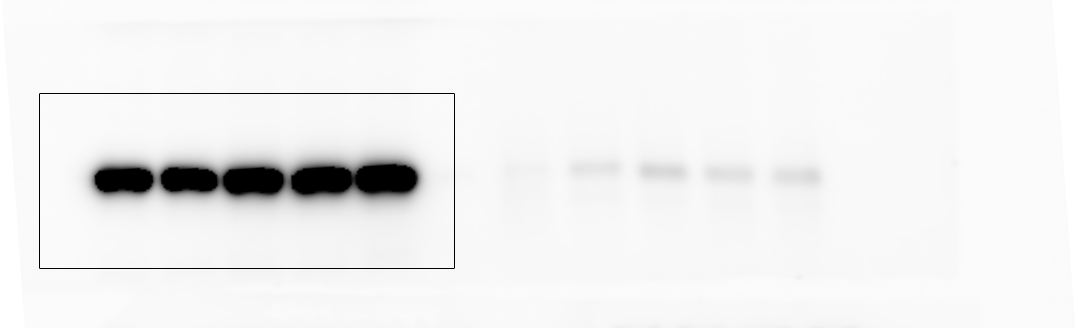

Supplement: Supplementary file 7 — Source data Fig. 4 [file 44318_2024_323_MOESM7_ESM.zip › SD figure 4/4A/western HA-INPUT.tif]

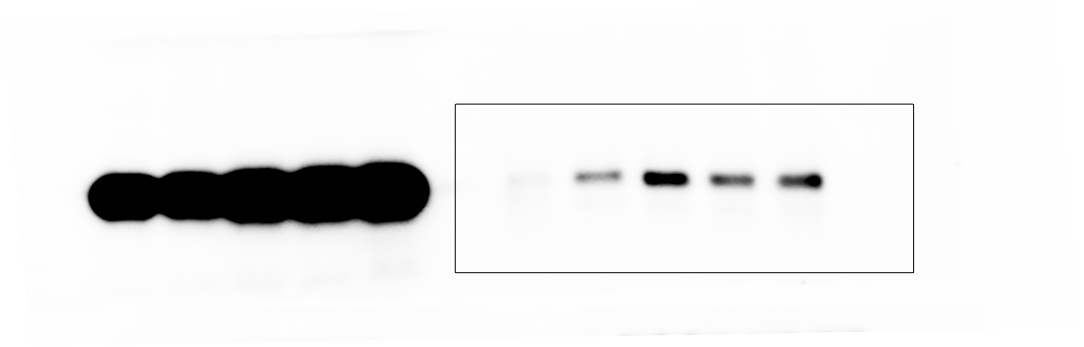

Supplement: Supplementary file 7 — Source data Fig. 4 [file 44318_2024_323_MOESM7_ESM.zip › SD figure 4/4A/western HA-IP.tif]

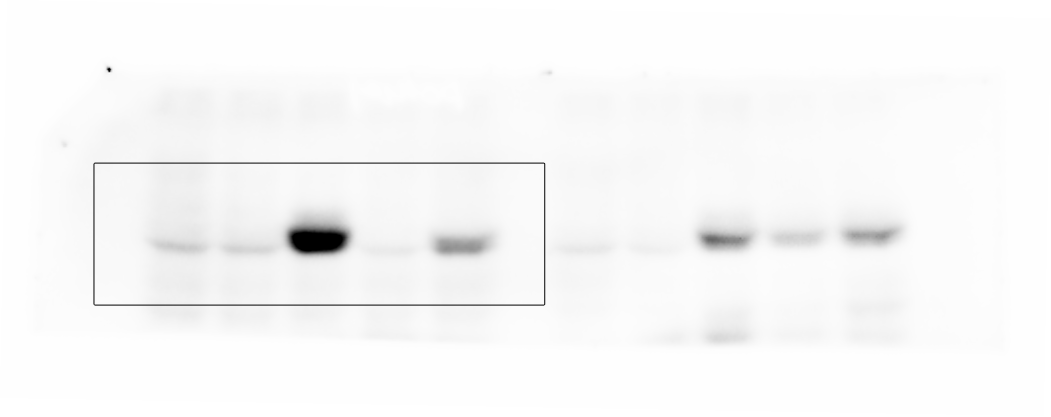

Supplement: Supplementary file 7 — Source data Fig. 4 [file 44318_2024_323_MOESM7_ESM.zip › SD figure 4/4A/western pCHK1S345.tif]

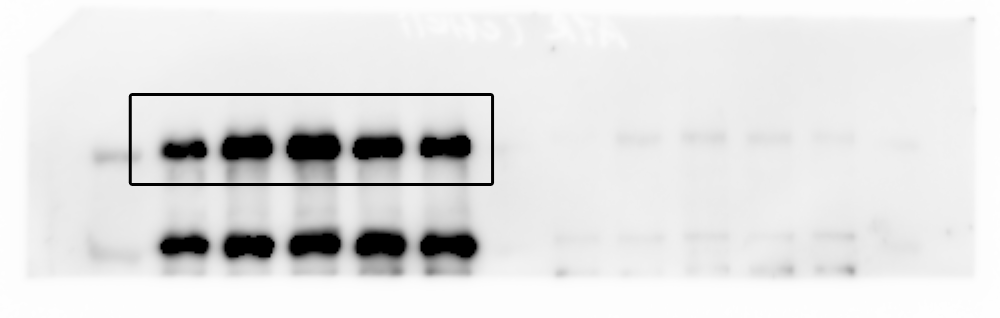

Supplement: Supplementary file 7 — Source data Fig. 4 [file 44318_2024_323_MOESM7_ESM.zip › SD figure 4/4B/western ATR INPUT.tif]

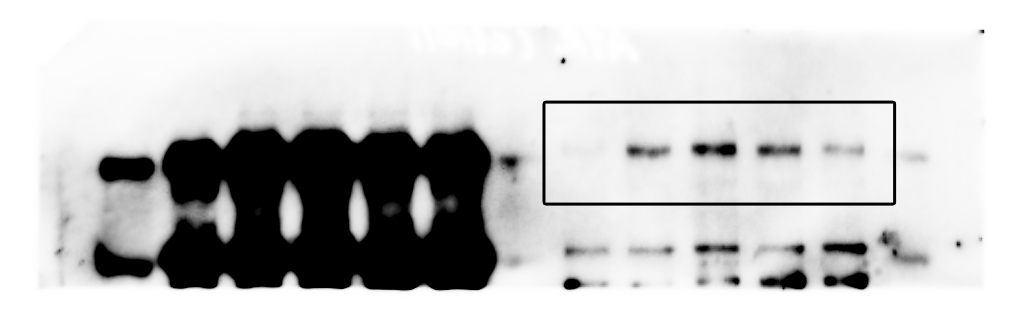

Supplement: Supplementary file 7 — Source data Fig. 4 [file 44318_2024_323_MOESM7_ESM.zip › SD figure 4/4B/western ATR IP.tif]

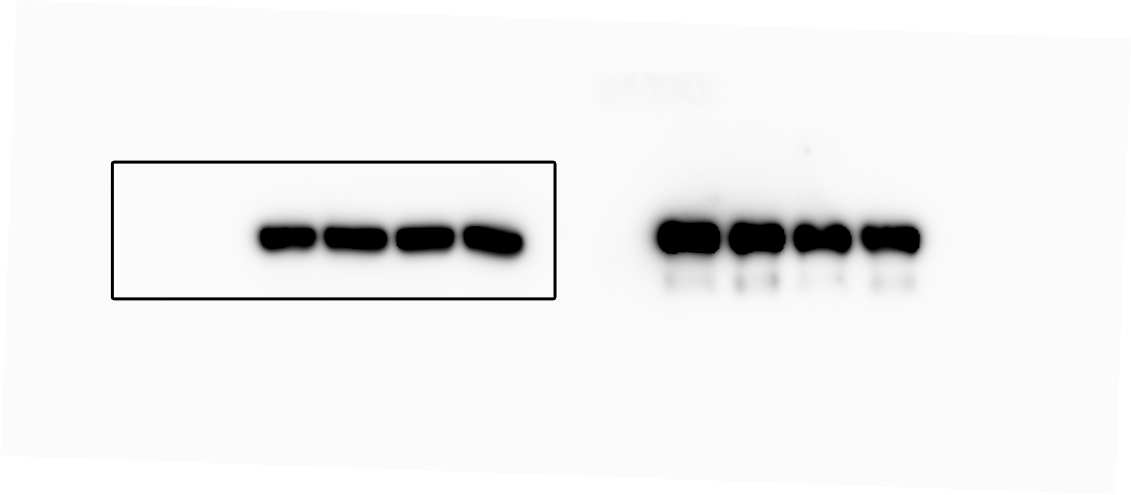

Supplement: Supplementary file 7 — Source data Fig. 4 [file 44318_2024_323_MOESM7_ESM.zip › SD figure 4/4B/western FLAG INPUT.tif]

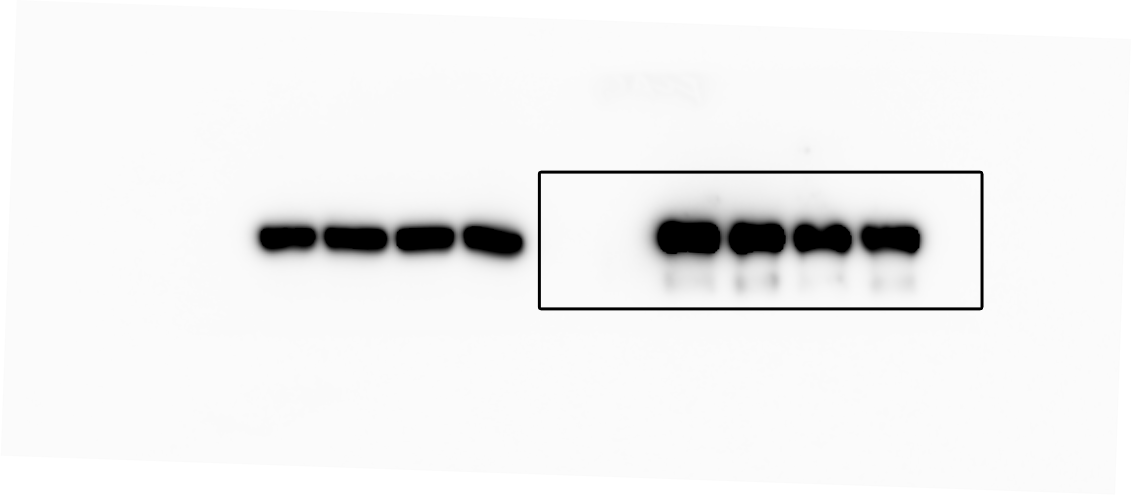

Supplement: Supplementary file 7 — Source data Fig. 4 [file 44318_2024_323_MOESM7_ESM.zip › SD figure 4/4B/western FLAG IP.tif]

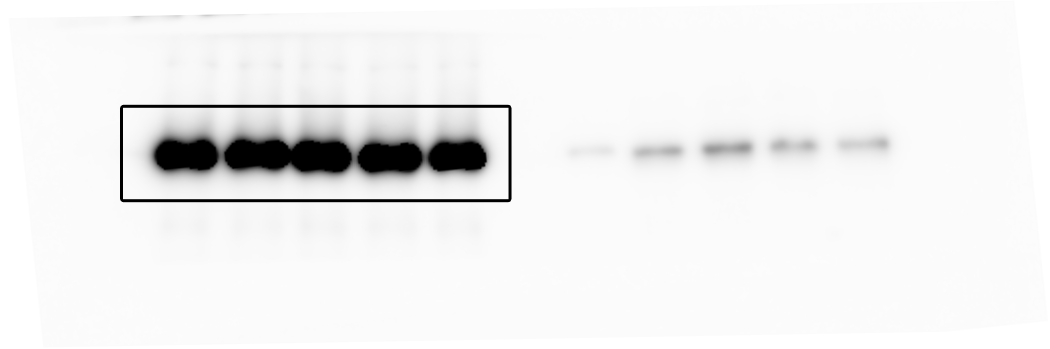

Supplement: Supplementary file 7 — Source data Fig. 4 [file 44318_2024_323_MOESM7_ESM.zip › SD figure 4/4B/western HA INPUT.tif]
