# Supplementary figures and images for: FOXP1 phosphorylation antagonizes its O-GlcNAcylation in regulating ATR activation in response to replication stress (part 2 of 3)
Source: EMBO J. 2024 Dec 2;44(2):457–83. doi: 10.1038/s44318-024-00323-x (PMC11729909; doi:10.1038/s44318-024-00323-x)

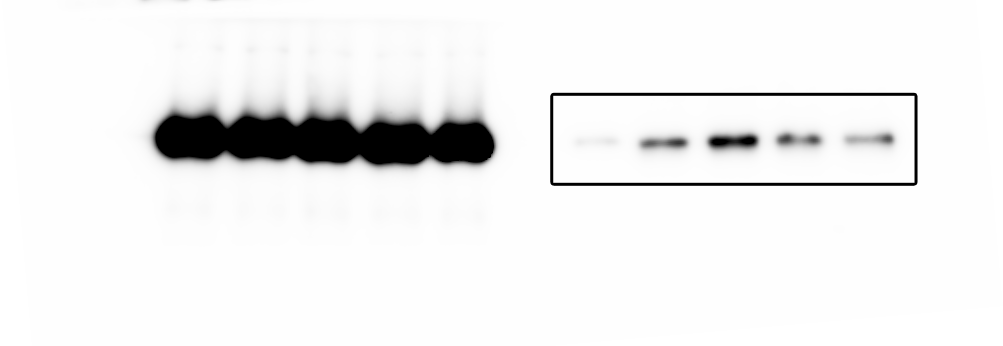

Supplement: Supplementary file 7 — Source data Fig. 4 [file 44318_2024_323_MOESM7_ESM.zip › SD figure 4/4B/western HA IP.tif]

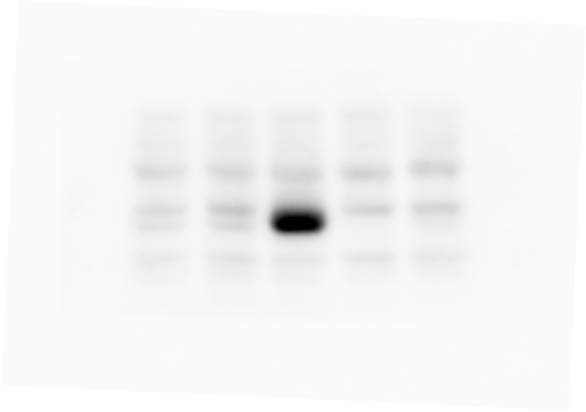

Supplement: Supplementary file 7 — Source data Fig. 4 [file 44318_2024_323_MOESM7_ESM.zip › SD figure 4/4B/western pCHK1 S296.tif]

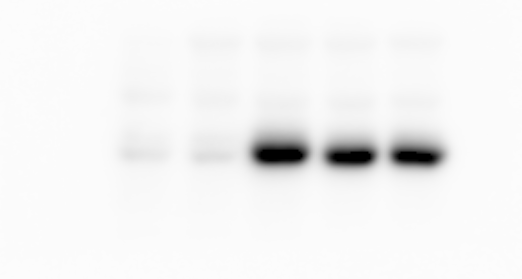

Supplement: Supplementary file 7 — Source data Fig. 4 [file 44318_2024_323_MOESM7_ESM.zip › SD figure 4/4B/western pCHK1S345.tif]

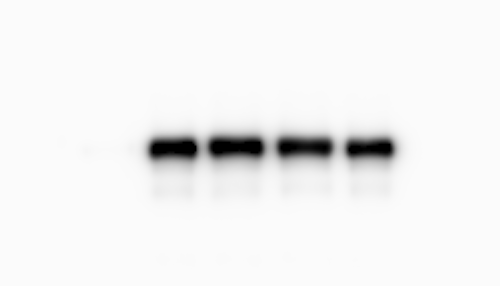

Supplement: Supplementary file 7 — Source data Fig. 4 [file 44318_2024_323_MOESM7_ESM.zip › SD figure 4/4C/western FLAG.tif]

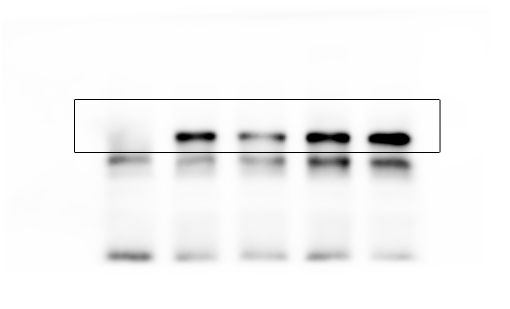

Supplement: Supplementary file 7 — Source data Fig. 4 [file 44318_2024_323_MOESM7_ESM.zip › SD figure 4/4C/western O-GlcNAc.tif]

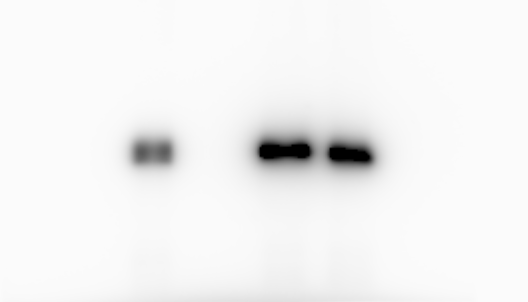

Supplement: Supplementary file 7 — Source data Fig. 4 [file 44318_2024_323_MOESM7_ESM.zip › SD figure 4/4D/western CHK1.tif]

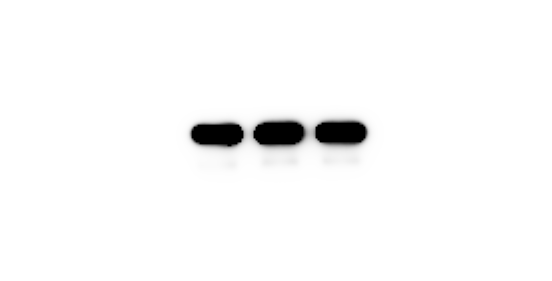

Supplement: Supplementary file 7 — Source data Fig. 4 [file 44318_2024_323_MOESM7_ESM.zip › SD figure 4/4D/western FOXP1.tif]

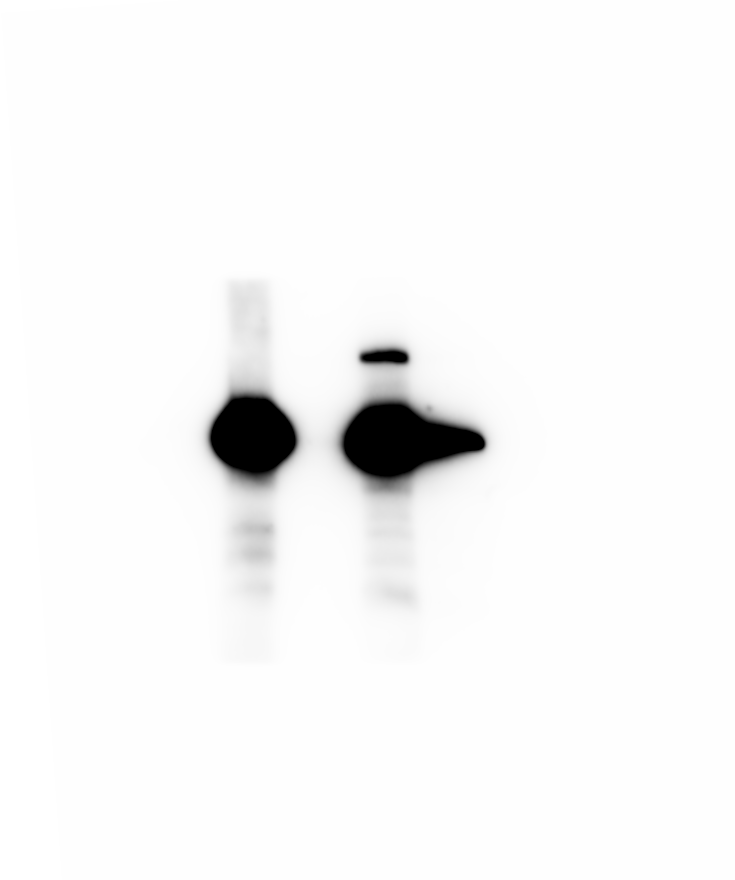

Supplement: Supplementary file 7 — Source data Fig. 4 [file 44318_2024_323_MOESM7_ESM.zip › SD figure 4/4D/western Thiophosphate ester LE.tif]

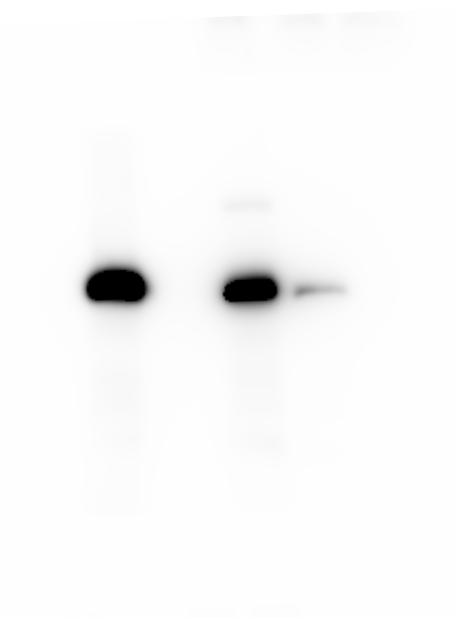

Supplement: Supplementary file 7 — Source data Fig. 4 [file 44318_2024_323_MOESM7_ESM.zip › SD figure 4/4D/western Thiophosphate ester SE.tif]

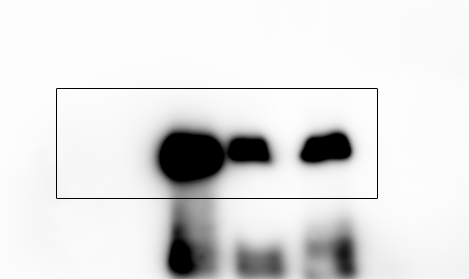

Supplement: Supplementary file 7 — Source data Fig. 4 [file 44318_2024_323_MOESM7_ESM.zip › SD figure 4/4E/western CHK1.tif]

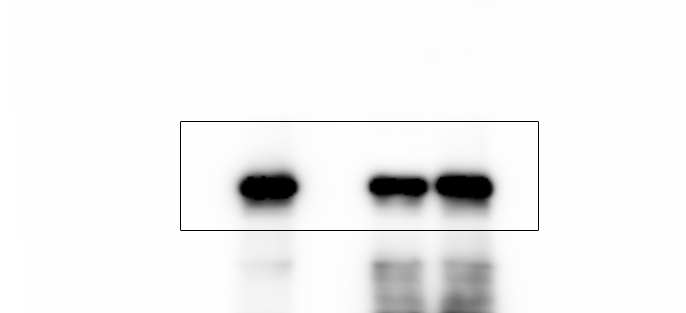

Supplement: Supplementary file 7 — Source data Fig. 4 [file 44318_2024_323_MOESM7_ESM.zip › SD figure 4/4E/western FOXP1.tif]

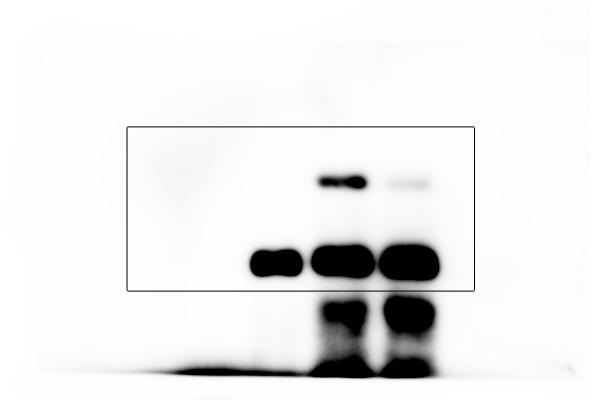

Supplement: Supplementary file 7 — Source data Fig. 4 [file 44318_2024_323_MOESM7_ESM.zip › SD figure 4/4E/western Thiophosphate ester.tif]

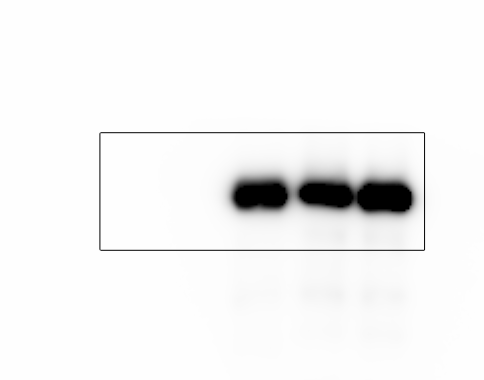

Supplement: Supplementary file 7 — Source data Fig. 4 [file 44318_2024_323_MOESM7_ESM.zip › SD figure 4/4F/western CHK1.tif]

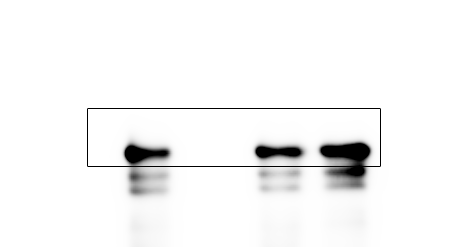

Supplement: Supplementary file 7 — Source data Fig. 4 [file 44318_2024_323_MOESM7_ESM.zip › SD figure 4/4F/western FOXP1.tif]

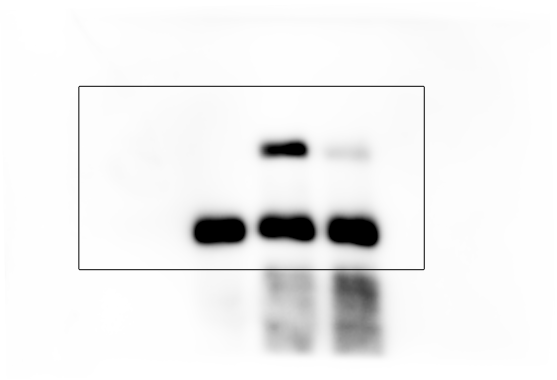

Supplement: Supplementary file 7 — Source data Fig. 4 [file 44318_2024_323_MOESM7_ESM.zip › SD figure 4/4F/western Thiophosphate ester.tif]

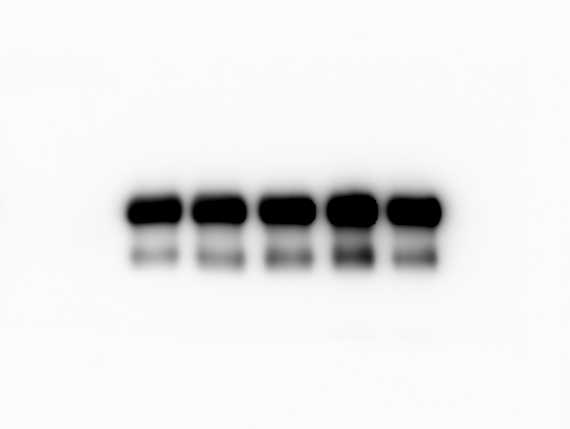

Supplement: Supplementary file 7 — Source data Fig. 4 [file 44318_2024_323_MOESM7_ESM.zip › SD figure 4/4G/western FLAG.tif]

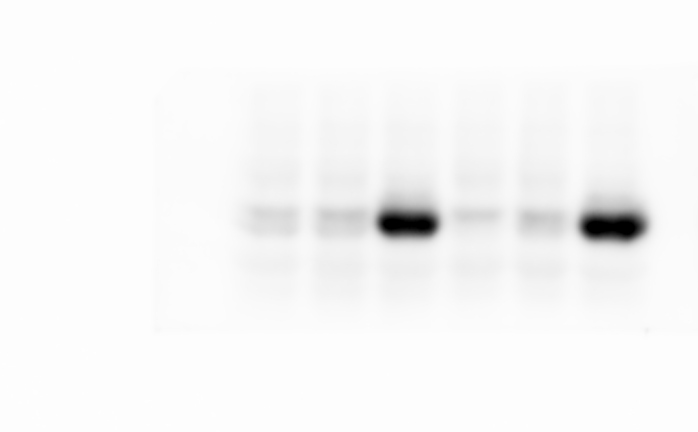

Supplement: Supplementary file 7 — Source data Fig. 4 [file 44318_2024_323_MOESM7_ESM.zip › SD figure 4/4G/western pCHK1S296.tif]

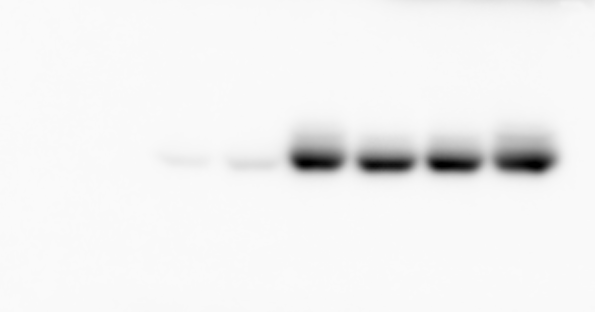

Supplement: Supplementary file 7 — Source data Fig. 4 [file 44318_2024_323_MOESM7_ESM.zip › SD figure 4/4G/western pCHK1S345.tif]

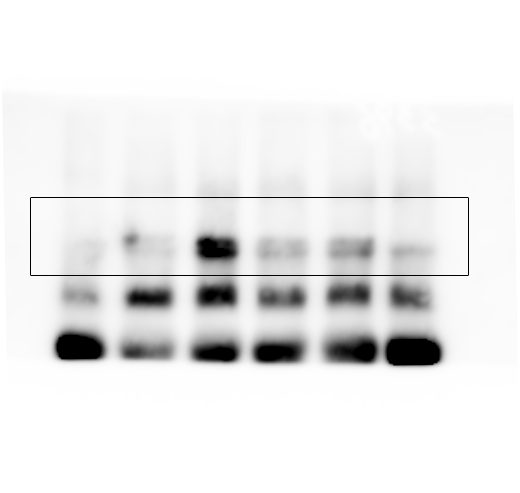

Supplement: Supplementary file 7 — Source data Fig. 4 [file 44318_2024_323_MOESM7_ESM.zip › SD figure 4/4G/western pFOXP1 S396.tif]

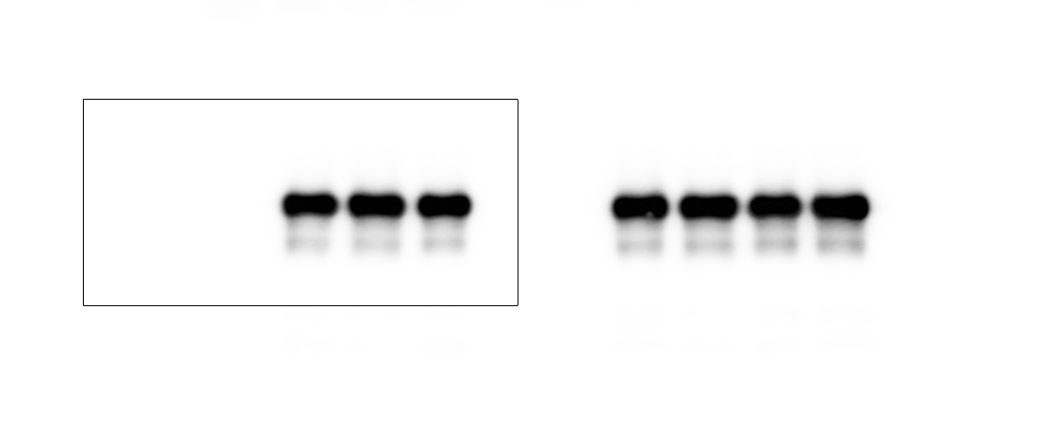

Supplement: Supplementary file 7 — Source data Fig. 4 [file 44318_2024_323_MOESM7_ESM.zip › SD figure 4/4H/western FLAG.tif]

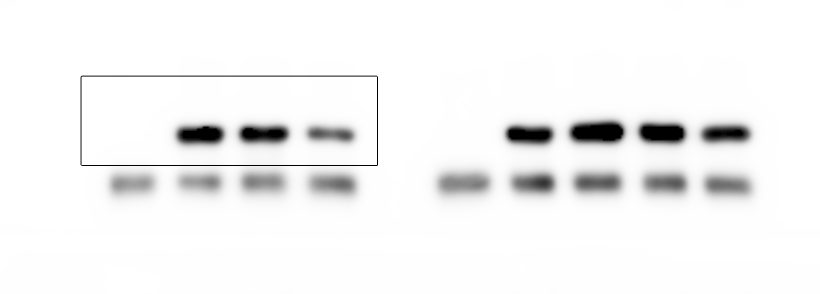

Supplement: Supplementary file 7 — Source data Fig. 4 [file 44318_2024_323_MOESM7_ESM.zip › SD figure 4/4H/western O-GlcNAc.tif]

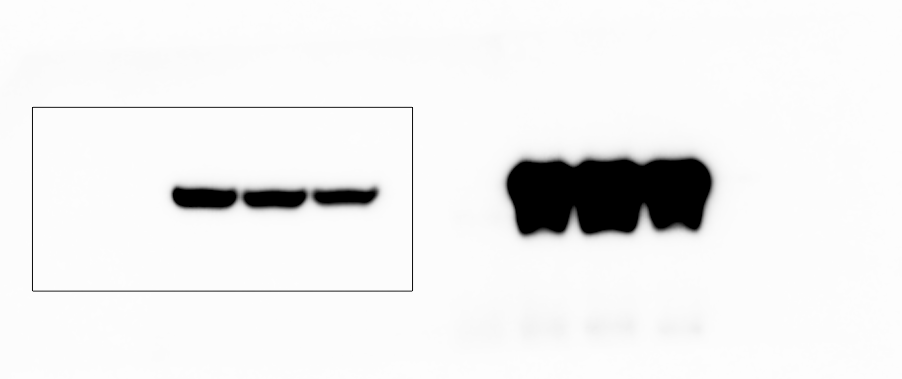

Supplement: Supplementary file 7 — Source data Fig. 4 [file 44318_2024_323_MOESM7_ESM.zip › SD figure 4/4I/western FLAG INPUT.tif]

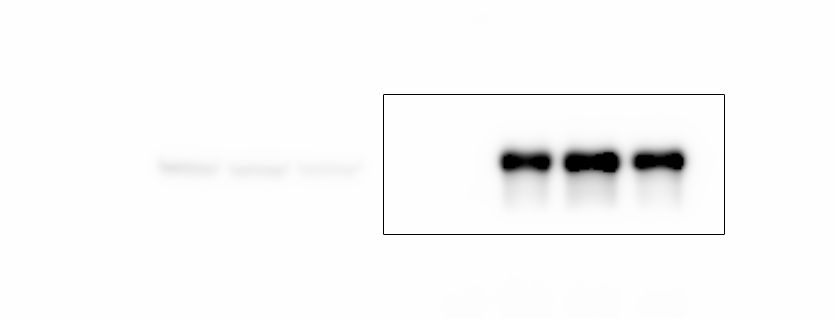

Supplement: Supplementary file 7 — Source data Fig. 4 [file 44318_2024_323_MOESM7_ESM.zip › SD figure 4/4I/western FLAG IP.tif]

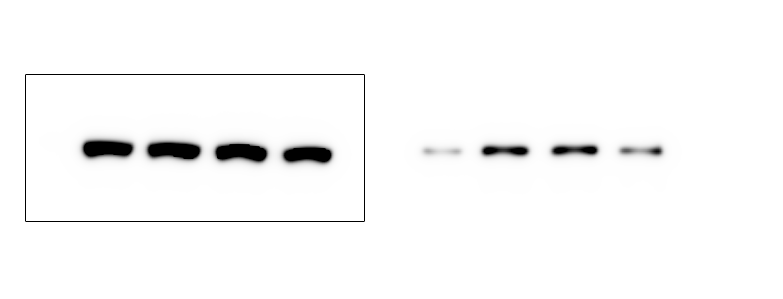

Supplement: Supplementary file 7 — Source data Fig. 4 [file 44318_2024_323_MOESM7_ESM.zip › SD figure 4/4I/western GFP INPUT.tif]

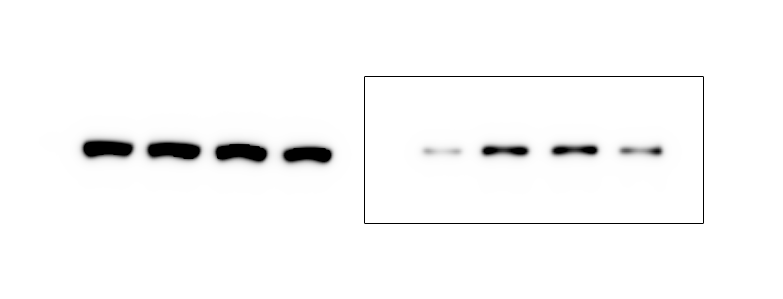

Supplement: Supplementary file 7 — Source data Fig. 4 [file 44318_2024_323_MOESM7_ESM.zip › SD figure 4/4I/western GFP IP.tif]

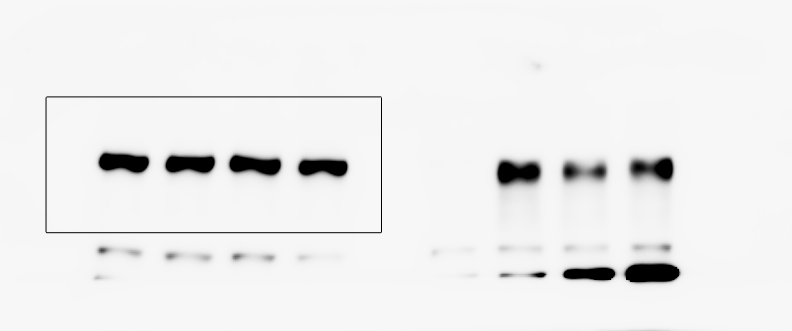

Supplement: Supplementary file 7 — Source data Fig. 4 [file 44318_2024_323_MOESM7_ESM.zip › SD figure 4/4J/western ATR INPUT.tif]

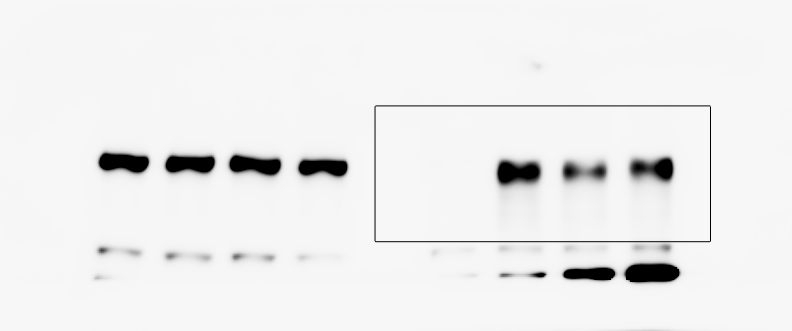

Supplement: Supplementary file 7 — Source data Fig. 4 [file 44318_2024_323_MOESM7_ESM.zip › SD figure 4/4J/western ATR IP.tif]

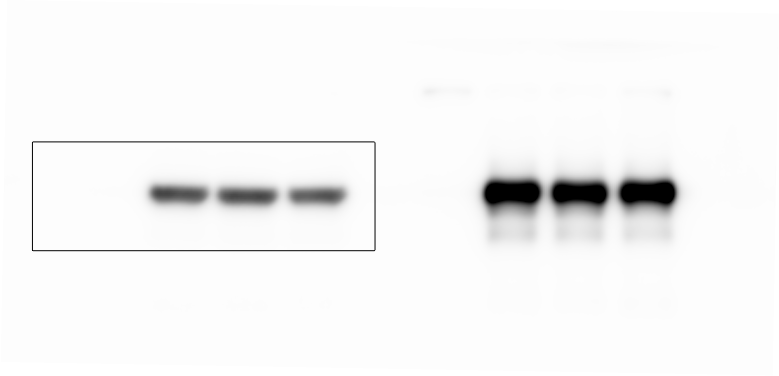

Supplement: Supplementary file 7 — Source data Fig. 4 [file 44318_2024_323_MOESM7_ESM.zip › SD figure 4/4J/western FLAG INPUT.tif]

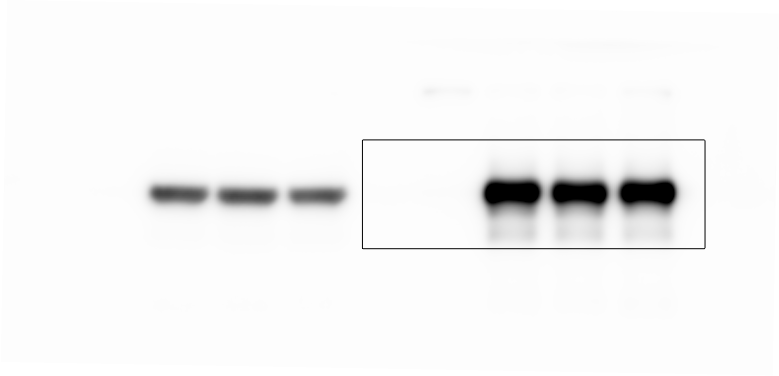

Supplement: Supplementary file 7 — Source data Fig. 4 [file 44318_2024_323_MOESM7_ESM.zip › SD figure 4/4J/western FLAG IP.tif]

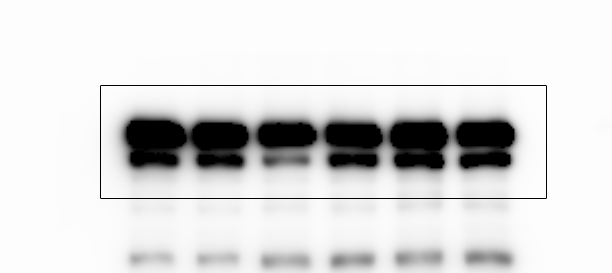

Supplement: Supplementary file 8 — Source data Fig. 5 [file 44318_2024_323_MOESM8_ESM.zip › SD figure 5/5A/western FOXP1 INPUT.tif]

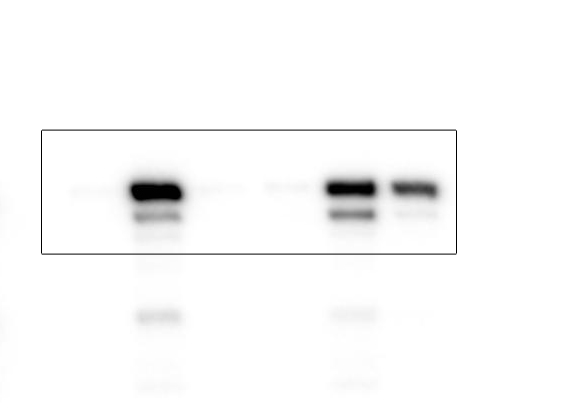

Supplement: Supplementary file 8 — Source data Fig. 5 [file 44318_2024_323_MOESM8_ESM.zip › SD figure 5/5A/western FOXP1 pulldown.tif]

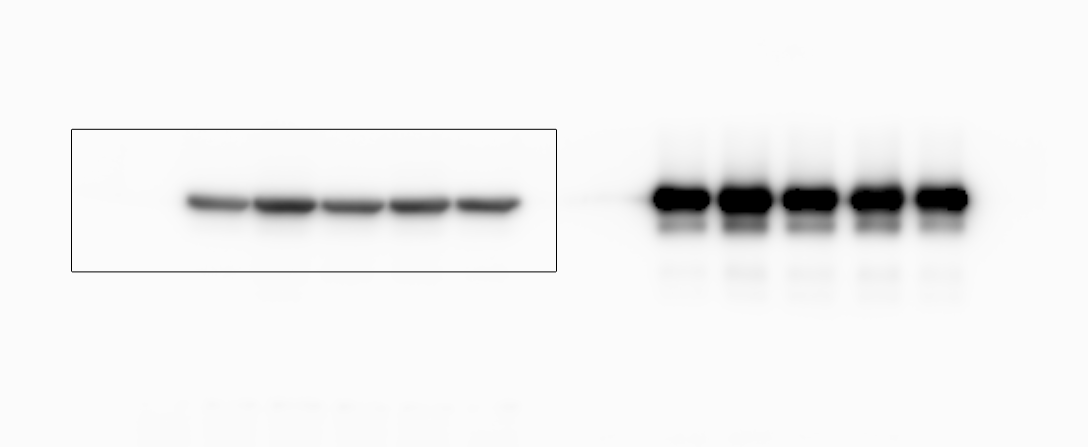

Supplement: Supplementary file 8 — Source data Fig. 5 [file 44318_2024_323_MOESM8_ESM.zip › SD figure 5/5B/western FLAG INPUT.tif]

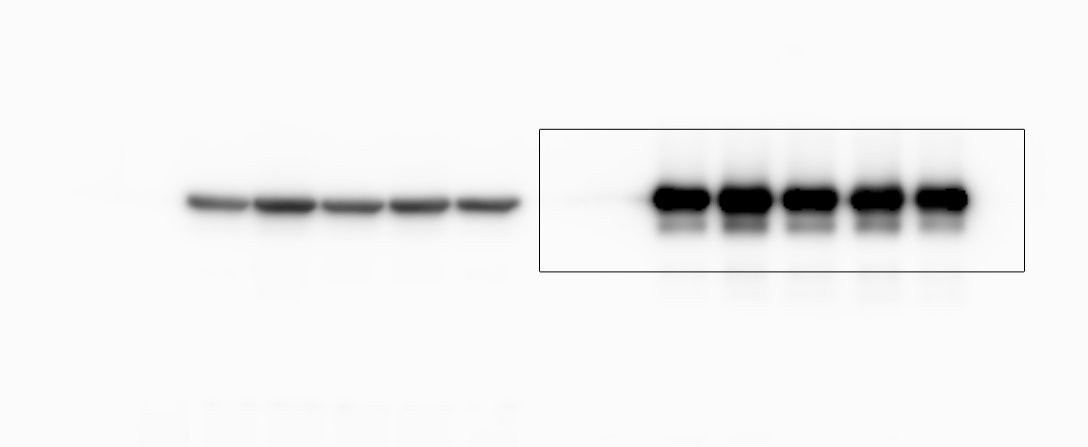

Supplement: Supplementary file 8 — Source data Fig. 5 [file 44318_2024_323_MOESM8_ESM.zip › SD figure 5/5B/western FLAG IP.tif]

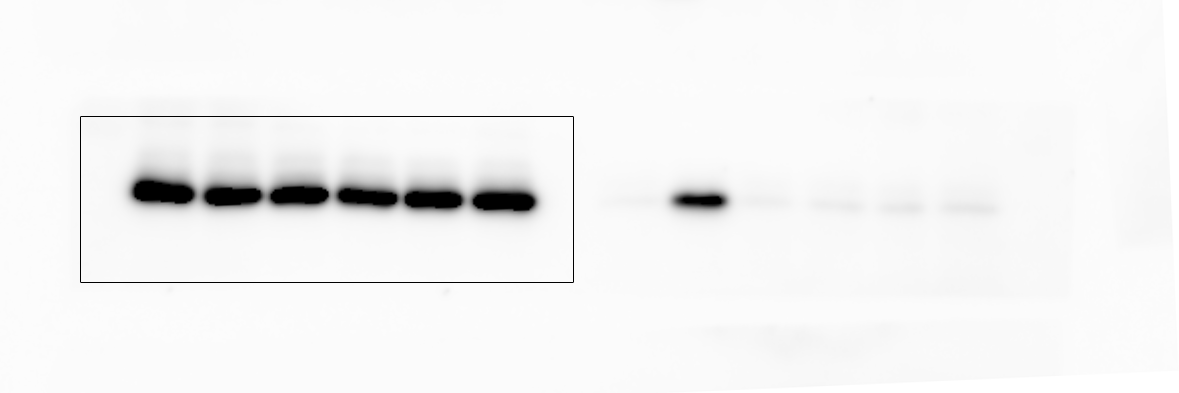

Supplement: Supplementary file 8 — Source data Fig. 5 [file 44318_2024_323_MOESM8_ESM.zip › SD figure 5/5B/western RPA32 INPUT.tif]

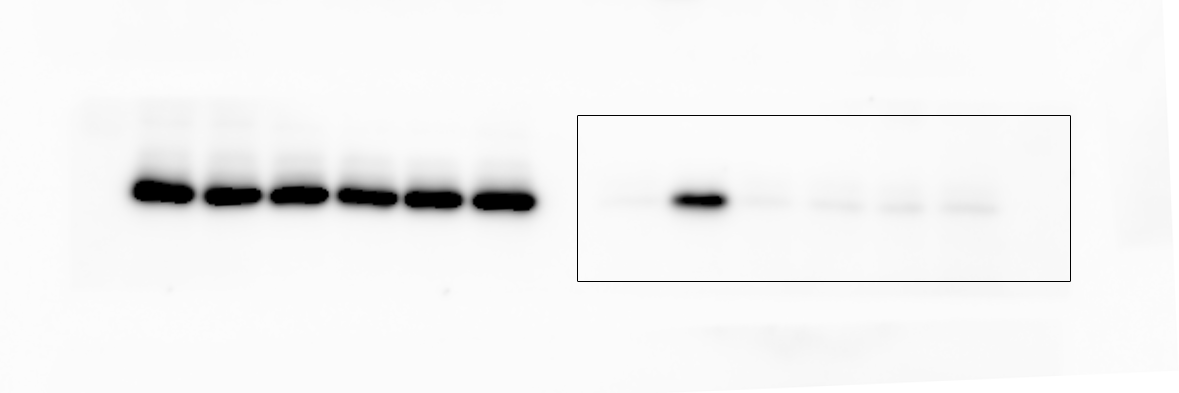

Supplement: Supplementary file 8 — Source data Fig. 5 [file 44318_2024_323_MOESM8_ESM.zip › SD figure 5/5B/western RPA32 IP.tif]

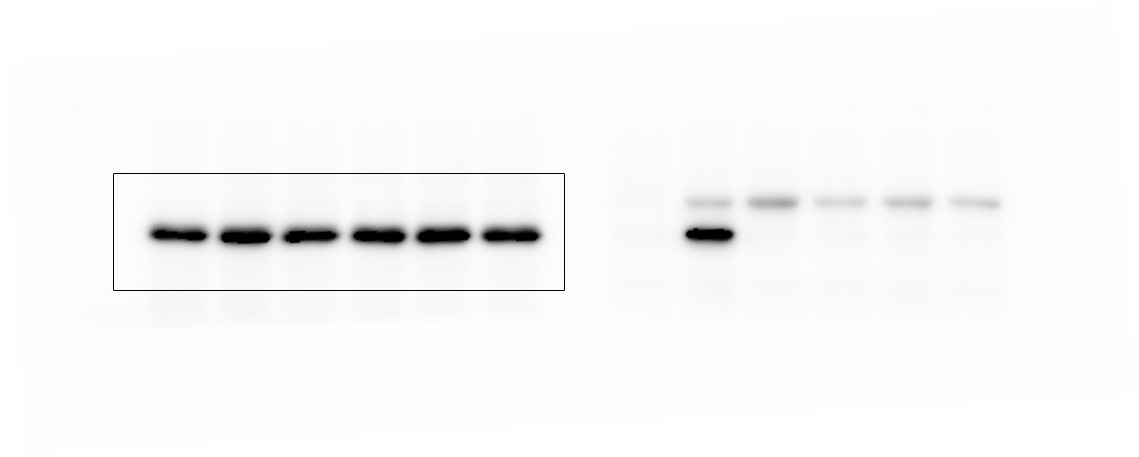

Supplement: Supplementary file 8 — Source data Fig. 5 [file 44318_2024_323_MOESM8_ESM.zip › SD figure 5/5B/western RPA70 INPUT.tif]

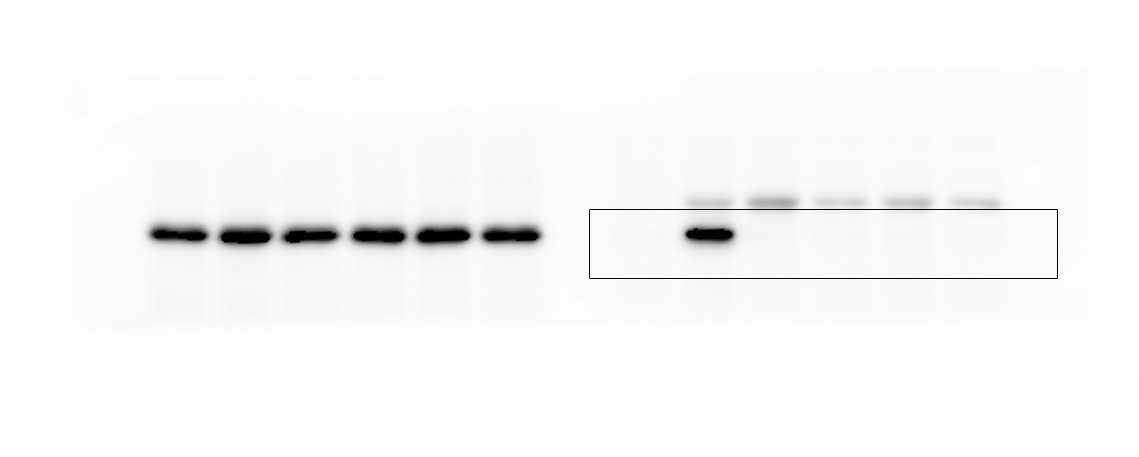

Supplement: Supplementary file 8 — Source data Fig. 5 [file 44318_2024_323_MOESM8_ESM.zip › SD figure 5/5B/western RPA70 IP.tif]

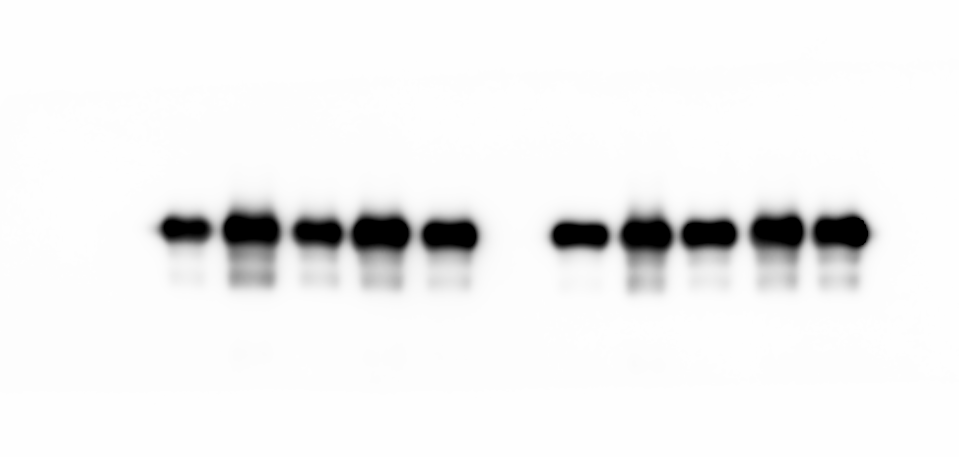

Supplement: Supplementary file 8 — Source data Fig. 5 [file 44318_2024_323_MOESM8_ESM.zip › SD figure 5/5C/western FLAG.tif]

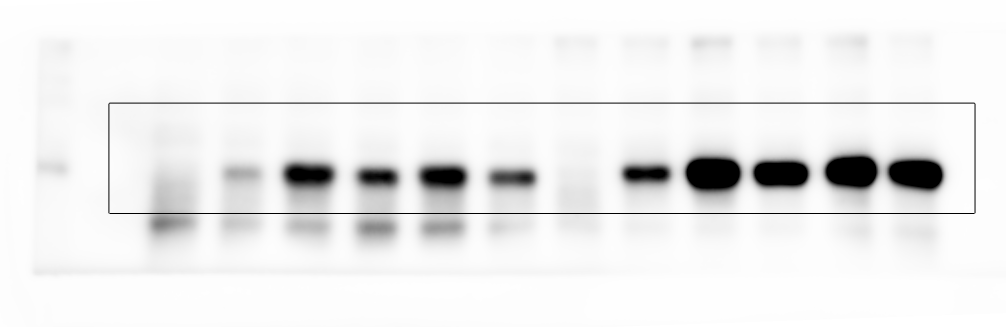

Supplement: Supplementary file 8 — Source data Fig. 5 [file 44318_2024_323_MOESM8_ESM.zip › SD figure 5/5C/western O-GlcNAc LE.tif]

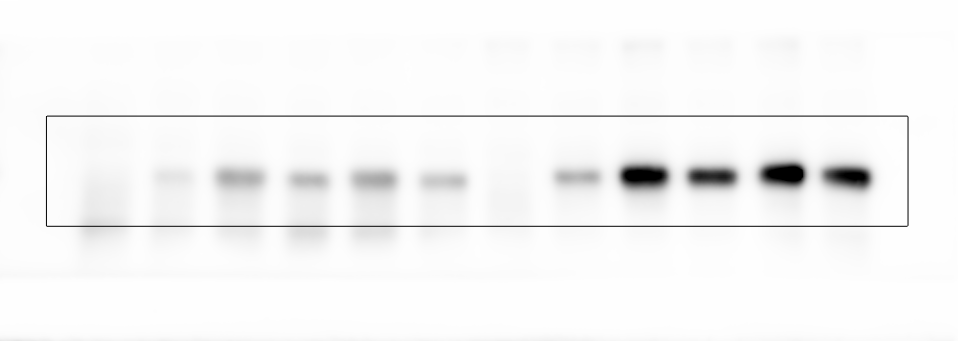

Supplement: Supplementary file 8 — Source data Fig. 5 [file 44318_2024_323_MOESM8_ESM.zip › SD figure 5/5C/western O-GlcNAc SE.tif]

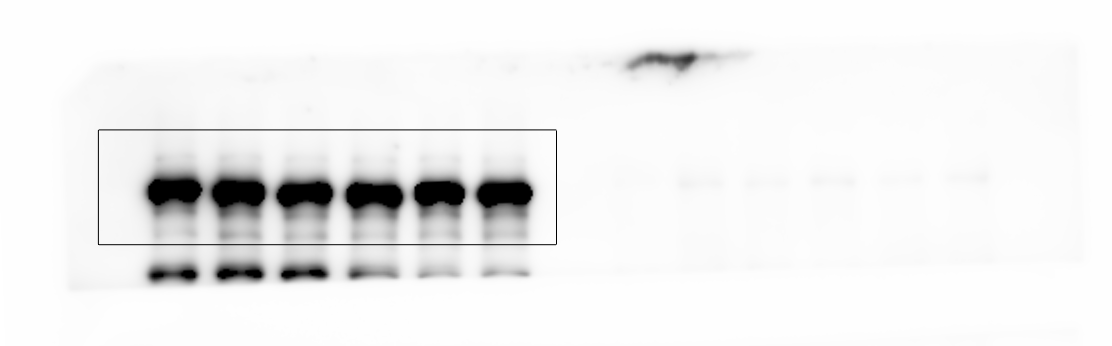

Supplement: Supplementary file 8 — Source data Fig. 5 [file 44318_2024_323_MOESM8_ESM.zip › SD figure 5/5D/western ATR INPUT.tif]

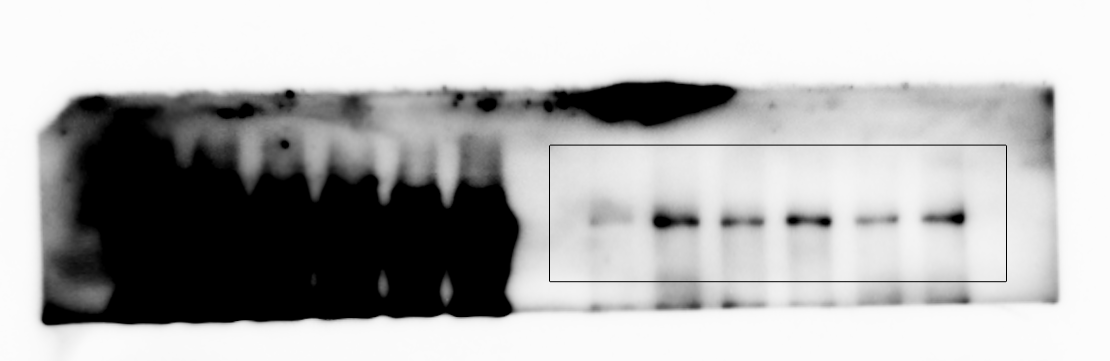

Supplement: Supplementary file 8 — Source data Fig. 5 [file 44318_2024_323_MOESM8_ESM.zip › SD figure 5/5D/western ATR IP.tif]

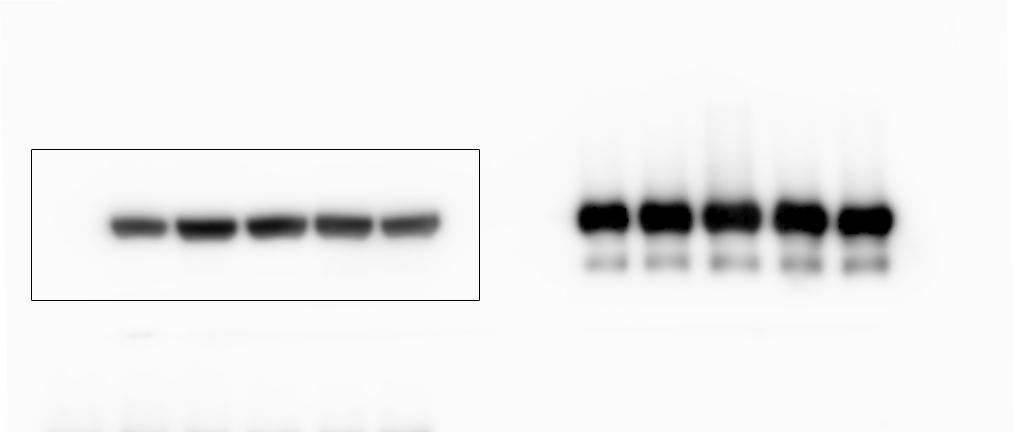

Supplement: Supplementary file 8 — Source data Fig. 5 [file 44318_2024_323_MOESM8_ESM.zip › SD figure 5/5D/western FLAG INPUT.tif]

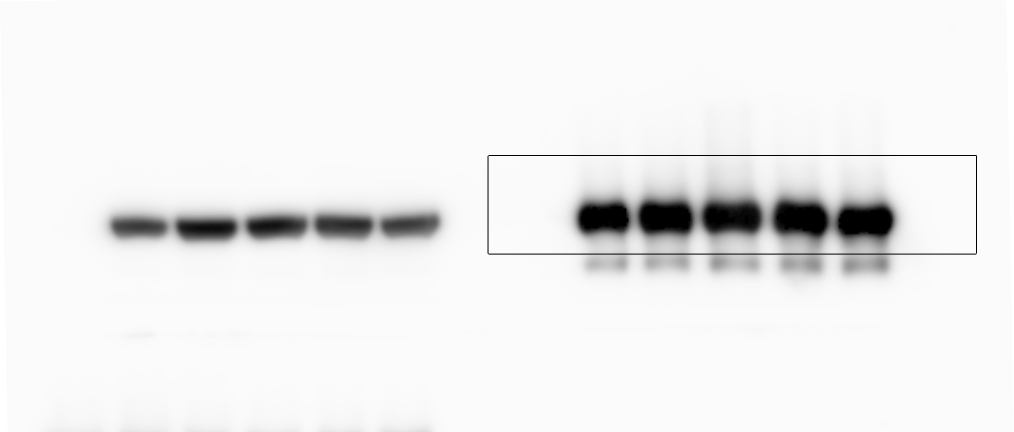

Supplement: Supplementary file 8 — Source data Fig. 5 [file 44318_2024_323_MOESM8_ESM.zip › SD figure 5/5D/western FLAG IP.tif]

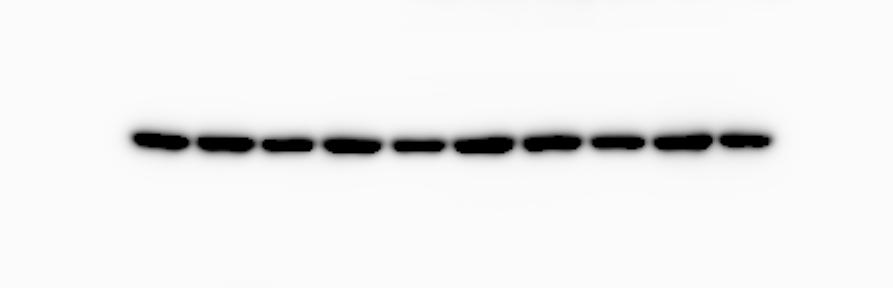

Supplement: Supplementary file 8 — Source data Fig. 5 [file 44318_2024_323_MOESM8_ESM.zip › SD figure 5/5E/western Actin.tif]

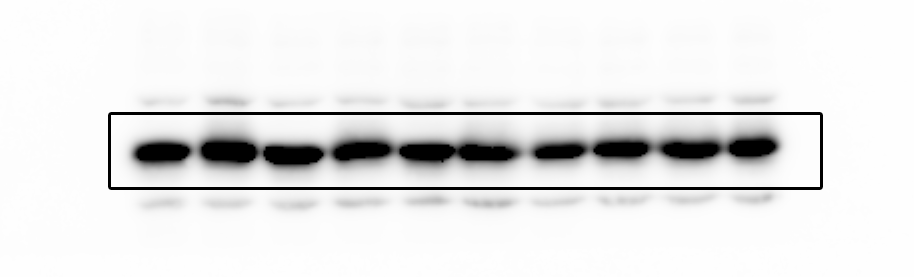

Supplement: Supplementary file 8 — Source data Fig. 5 [file 44318_2024_323_MOESM8_ESM.zip › SD figure 5/5E/western CHK1.tif]

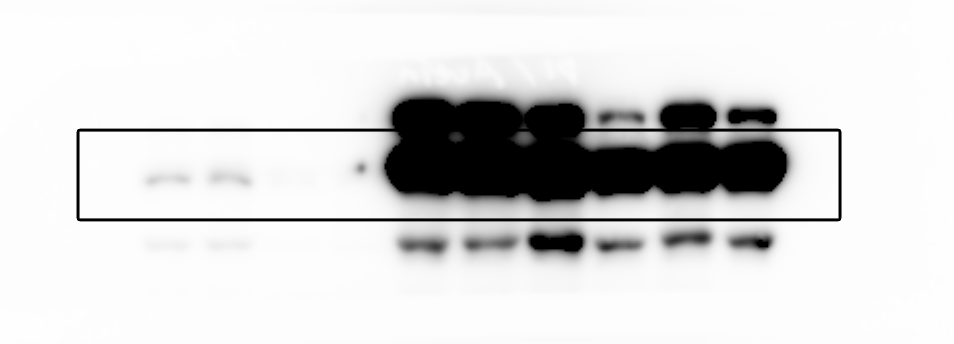

Supplement: Supplementary file 8 — Source data Fig. 5 [file 44318_2024_323_MOESM8_ESM.zip › SD figure 5/5E/western FOXP1 LE.tif]

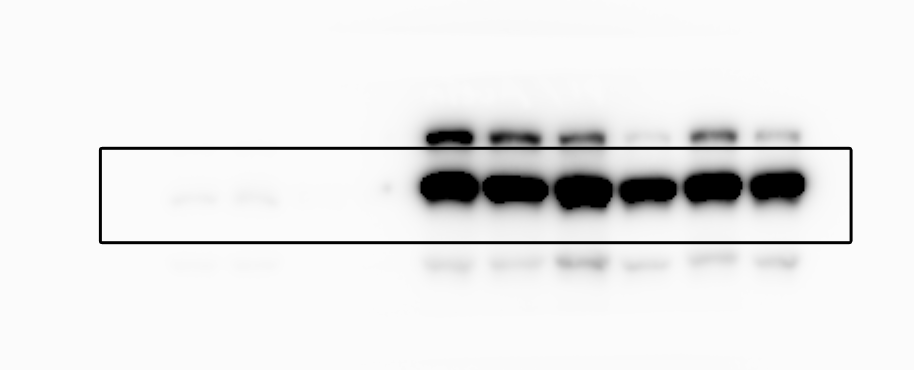

Supplement: Supplementary file 8 — Source data Fig. 5 [file 44318_2024_323_MOESM8_ESM.zip › SD figure 5/5E/western FOXP1 SE.tif]

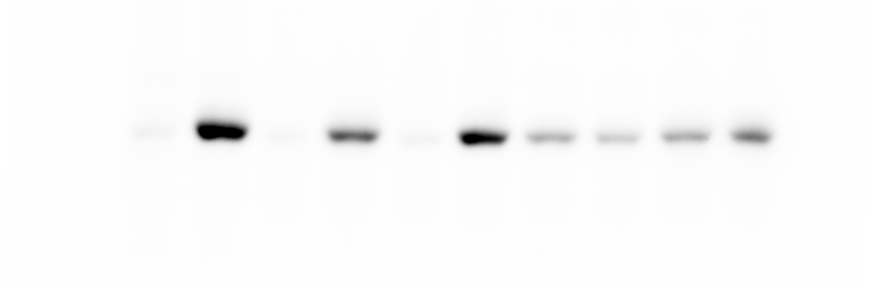

Supplement: Supplementary file 8 — Source data Fig. 5 [file 44318_2024_323_MOESM8_ESM.zip › SD figure 5/5E/western pCHK1S345.tif]

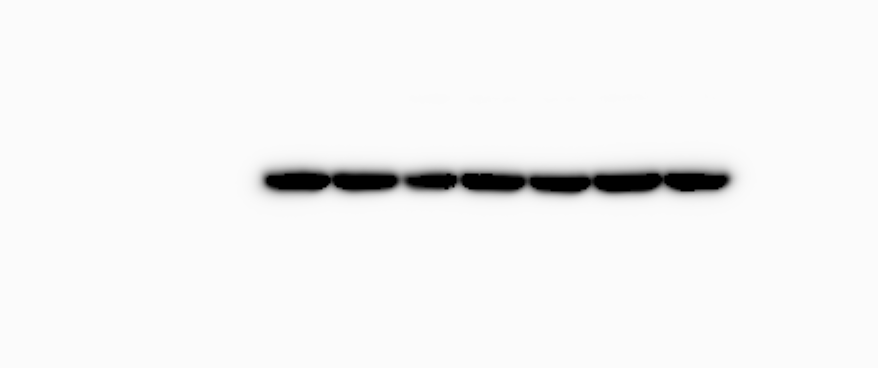

Supplement: Supplementary file 8 — Source data Fig. 5 [file 44318_2024_323_MOESM8_ESM.zip › SD figure 5/5F/western Actin.tif]

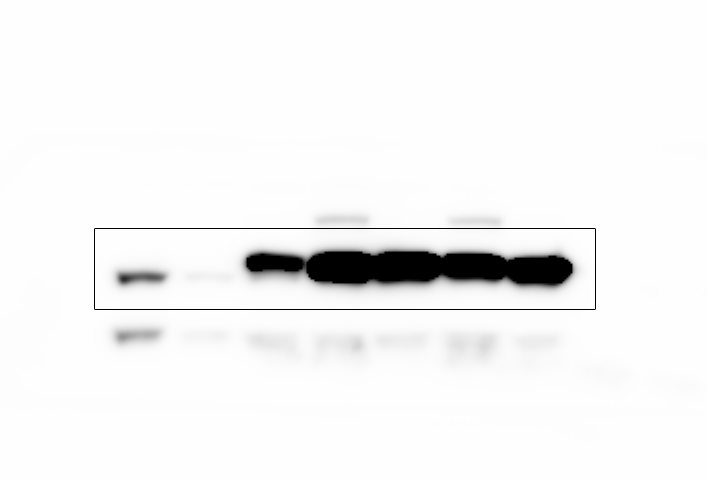

Supplement: Supplementary file 8 — Source data Fig. 5 [file 44318_2024_323_MOESM8_ESM.zip › SD figure 5/5F/western FOXP1.tif]

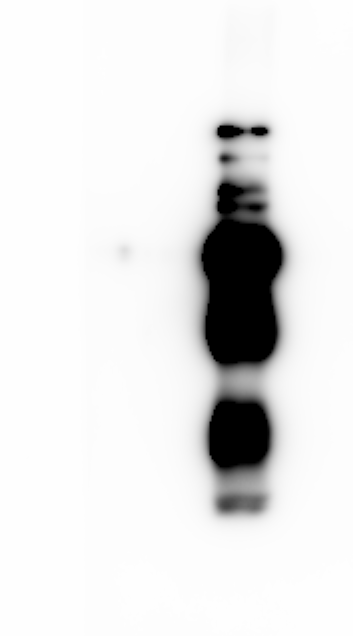

Supplement: Supplementary file 9 — Figure EV1-5 Source Data [file 44318_2024_323_MOESM9_ESM.zip › SD figure EV1-5/SD figure EV1/EV 1B/western ATRIP.tif]

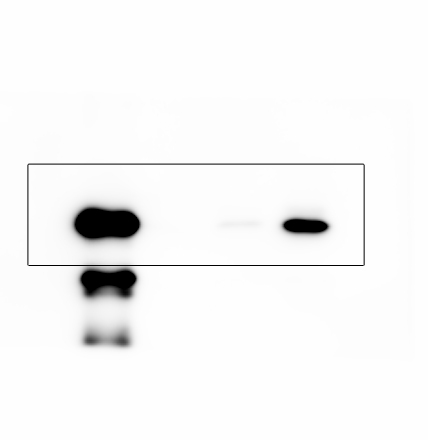

Supplement: Supplementary file 9 — Figure EV1-5 Source Data [file 44318_2024_323_MOESM9_ESM.zip › SD figure EV1-5/SD figure EV1/EV 1B/western FOXP1.tif]

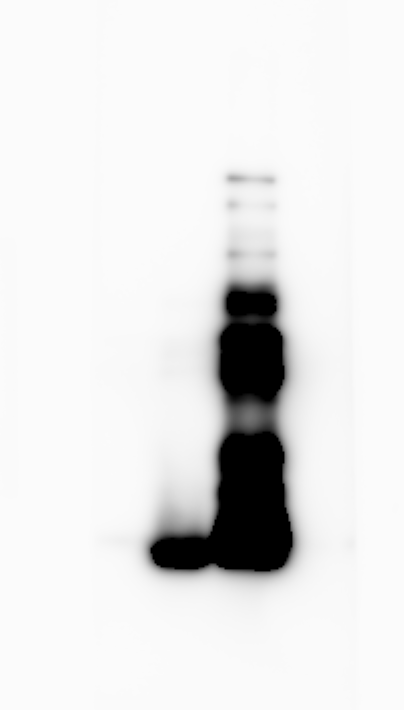

Supplement: Supplementary file 9 — Figure EV1-5 Source Data [file 44318_2024_323_MOESM9_ESM.zip › SD figure EV1-5/SD figure EV1/EV 1B/western GST.tif]

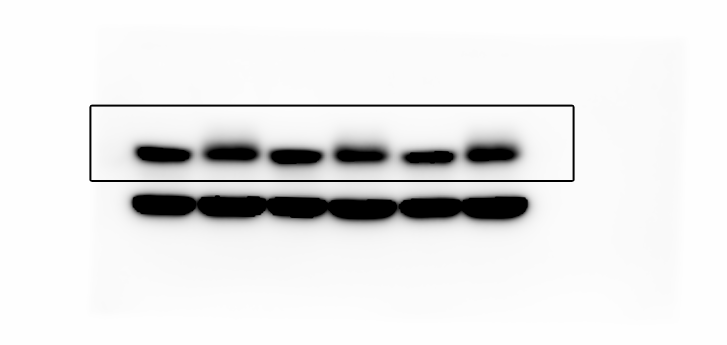

Supplement: Supplementary file 9 — Figure EV1-5 Source Data [file 44318_2024_323_MOESM9_ESM.zip › SD figure EV1-5/SD figure EV1/EV 1C/western CHK1.tif]

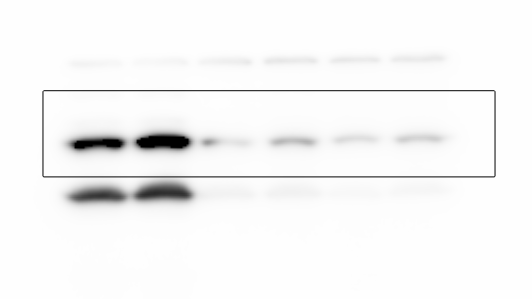

Supplement: Supplementary file 9 — Figure EV1-5 Source Data [file 44318_2024_323_MOESM9_ESM.zip › SD figure EV1-5/SD figure EV1/EV 1C/western FOXP1.tif]

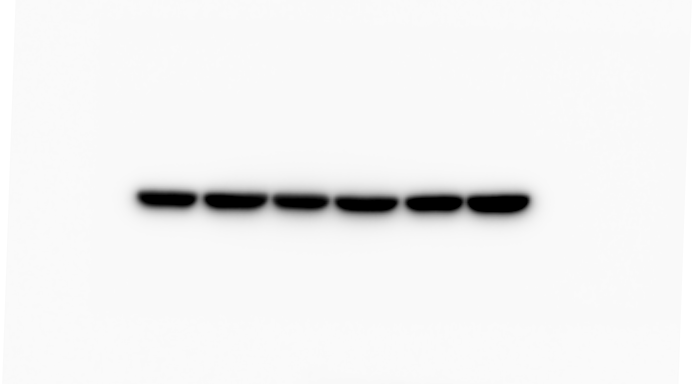

Supplement: Supplementary file 9 — Figure EV1-5 Source Data [file 44318_2024_323_MOESM9_ESM.zip › SD figure EV1-5/SD figure EV1/EV 1C/western actin.tif]

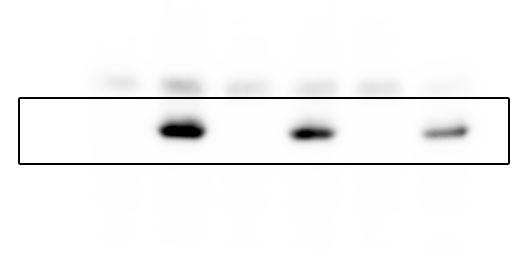

Supplement: Supplementary file 9 — Figure EV1-5 Source Data [file 44318_2024_323_MOESM9_ESM.zip › SD figure EV1-5/SD figure EV1/EV 1C/western pCHK1 S345.tif]

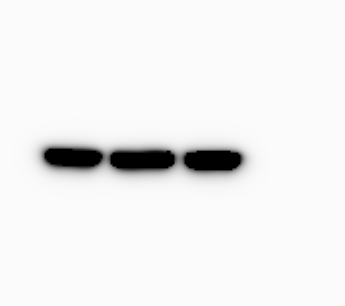

Supplement: Supplementary file 9 — Figure EV1-5 Source Data [file 44318_2024_323_MOESM9_ESM.zip › SD figure EV1-5/SD figure EV1/EV 1E/western Actin H1975.tif]

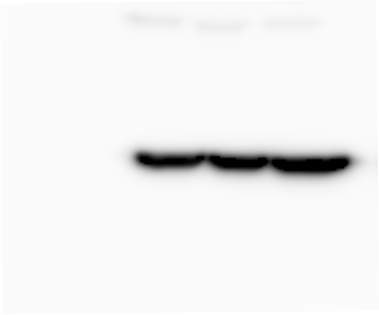

Supplement: Supplementary file 9 — Figure EV1-5 Source Data [file 44318_2024_323_MOESM9_ESM.zip › SD figure EV1-5/SD figure EV1/EV 1E/western Actin HEK293T.tif]

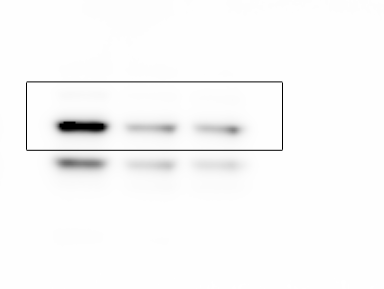

Supplement: Supplementary file 9 — Figure EV1-5 Source Data [file 44318_2024_323_MOESM9_ESM.zip › SD figure EV1-5/SD figure EV1/EV 1E/western FOXP1 H1975.tif]

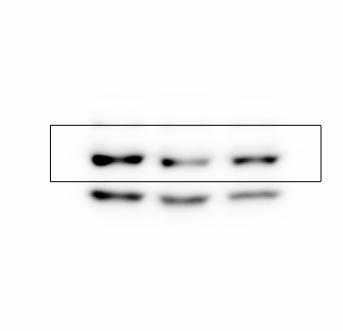

Supplement: Supplementary file 9 — Figure EV1-5 Source Data [file 44318_2024_323_MOESM9_ESM.zip › SD figure EV1-5/SD figure EV1/EV 1E/western FOXP1 HEK293T.tif]

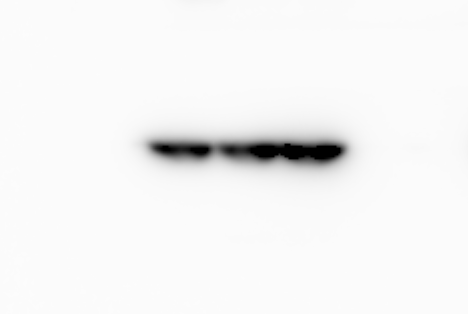

Supplement: Supplementary file 9 — Figure EV1-5 Source Data [file 44318_2024_323_MOESM9_ESM.zip › SD figure EV1-5/SD figure EV1/EV 1F/western Actin.tif]

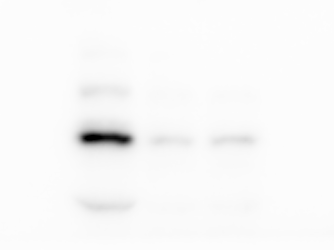

Supplement: Supplementary file 9 — Figure EV1-5 Source Data [file 44318_2024_323_MOESM9_ESM.zip › SD figure EV1-5/SD figure EV1/EV 1F/western FOXP1.tif]

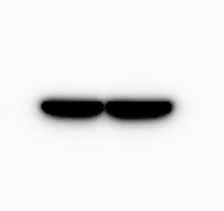

Supplement: Supplementary file 9 — Figure EV1-5 Source Data [file 44318_2024_323_MOESM9_ESM.zip › SD figure EV1-5/SD figure EV1/EV 1G/western Actin H1975.tif]

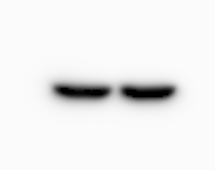

Supplement: Supplementary file 9 — Figure EV1-5 Source Data [file 44318_2024_323_MOESM9_ESM.zip › SD figure EV1-5/SD figure EV1/EV 1G/western Actin HEK293T.tif]

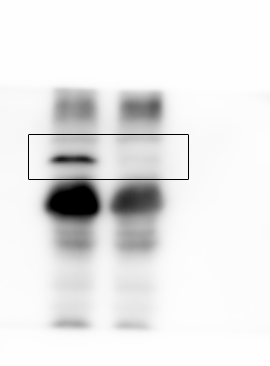

Supplement: Supplementary file 9 — Figure EV1-5 Source Data [file 44318_2024_323_MOESM9_ESM.zip › SD figure EV1-5/SD figure EV1/EV 1G/western FOXP1 H1975.tif]

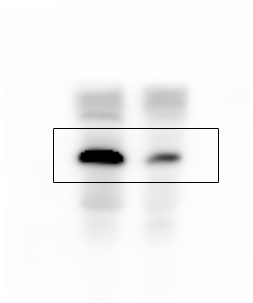

Supplement: Supplementary file 9 — Figure EV1-5 Source Data [file 44318_2024_323_MOESM9_ESM.zip › SD figure EV1-5/SD figure EV1/EV 1G/western FOXP1 HEK293T.tif]

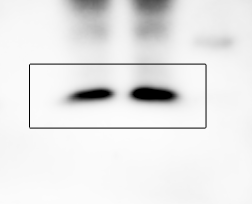

Supplement: Supplementary file 9 — Figure EV1-5 Source Data [file 44318_2024_323_MOESM9_ESM.zip › SD figure EV1-5/SD figure EV1/EV 1G/western GINS1 H1975.tif]

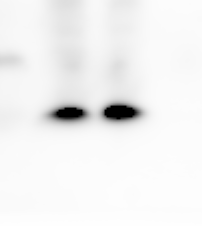

Supplement: Supplementary file 9 — Figure EV1-5 Source Data [file 44318_2024_323_MOESM9_ESM.zip › SD figure EV1-5/SD figure EV1/EV 1G/western GINS1 HEK293T.tif]

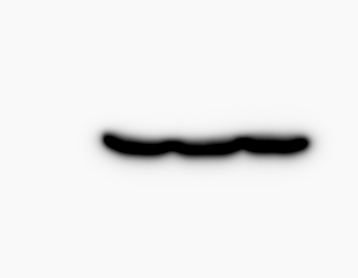

Supplement: Supplementary file 9 — Figure EV1-5 Source Data [file 44318_2024_323_MOESM9_ESM.zip › SD figure EV1-5/SD figure EV1/EV 1H/wester Actin.tif]

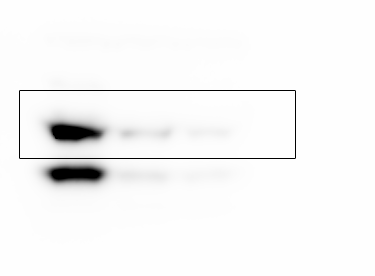

Supplement: Supplementary file 9 — Figure EV1-5 Source Data [file 44318_2024_323_MOESM9_ESM.zip › SD figure EV1-5/SD figure EV1/EV 1H/wester FOXP1.tif]

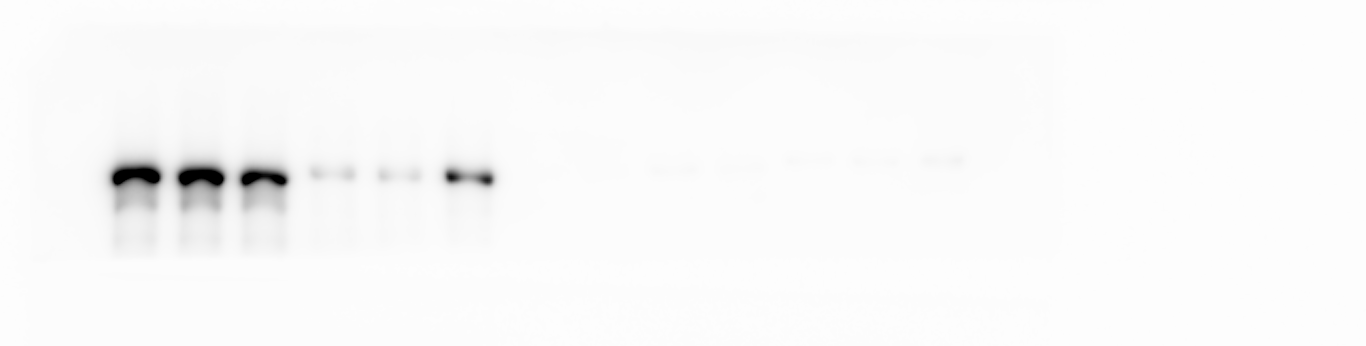

Supplement: Supplementary file 9 — Figure EV1-5 Source Data [file 44318_2024_323_MOESM9_ESM.zip › SD figure EV1-5/SD figure EV1/EV 1I/wester ATR INPUT.tif]

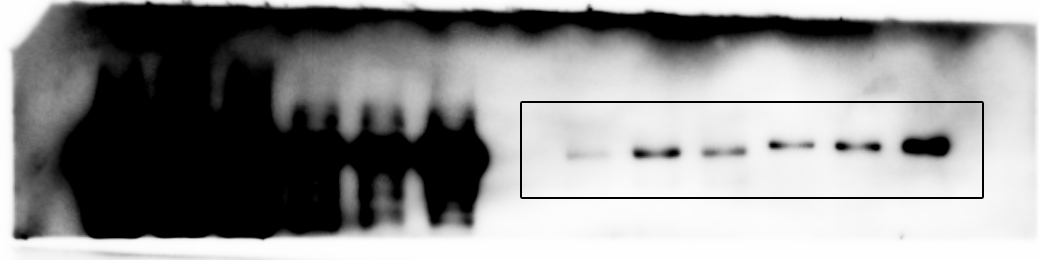

Supplement: Supplementary file 9 — Figure EV1-5 Source Data [file 44318_2024_323_MOESM9_ESM.zip › SD figure EV1-5/SD figure EV1/EV 1I/wester ATR IP.tif]

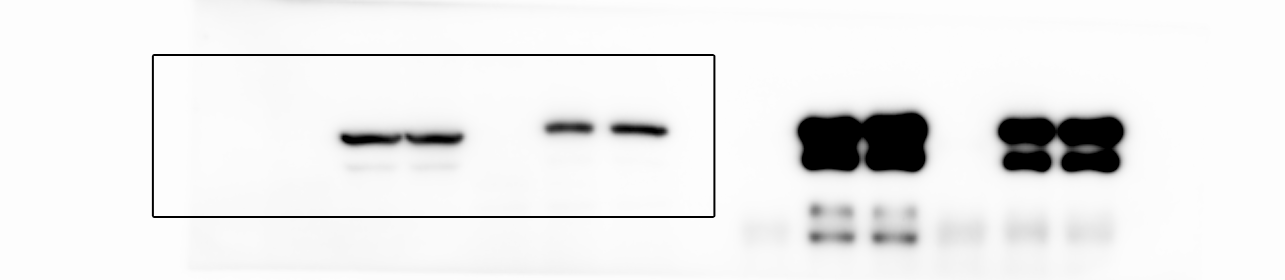

Supplement: Supplementary file 9 — Figure EV1-5 Source Data [file 44318_2024_323_MOESM9_ESM.zip › SD figure EV1-5/SD figure EV1/EV 1I/wester FLAG INPUT.tif]

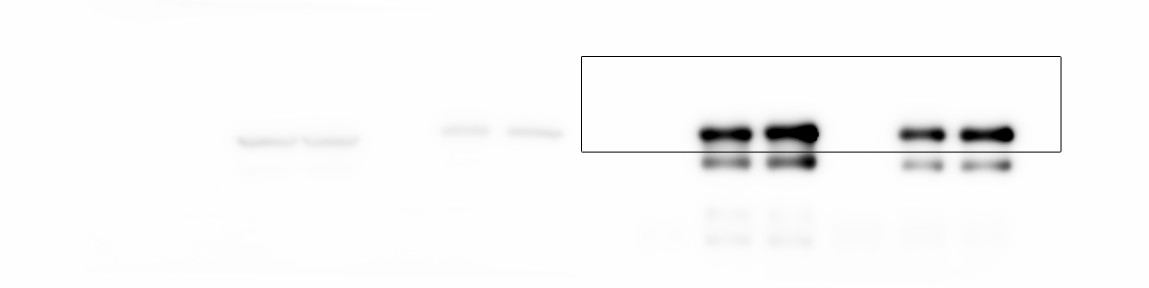

Supplement: Supplementary file 9 — Figure EV1-5 Source Data [file 44318_2024_323_MOESM9_ESM.zip › SD figure EV1-5/SD figure EV1/EV 1I/wester FLAG IP.tif]

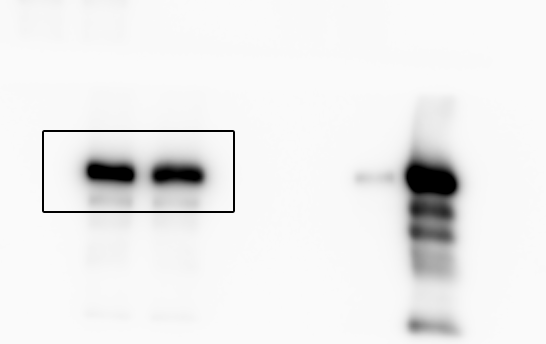

Supplement: Supplementary file 9 — Figure EV1-5 Source Data [file 44318_2024_323_MOESM9_ESM.zip › SD figure EV1-5/SD figure EV2/EV 2A/western FOXP1 INPUT.tif]

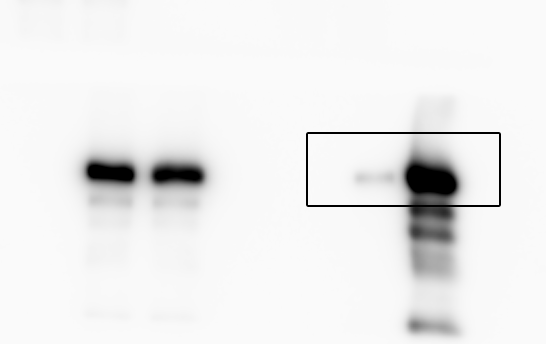

Supplement: Supplementary file 9 — Figure EV1-5 Source Data [file 44318_2024_323_MOESM9_ESM.zip › SD figure EV1-5/SD figure EV2/EV 2A/western FOXP1 pull-down.tif]

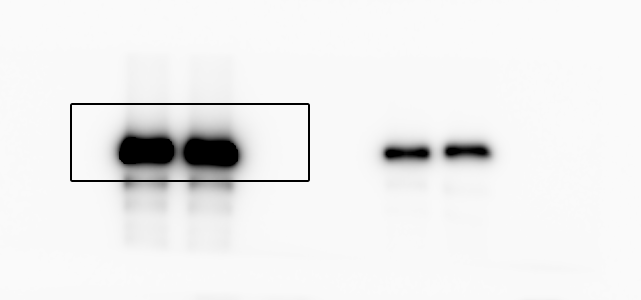

Supplement: Supplementary file 9 — Figure EV1-5 Source Data [file 44318_2024_323_MOESM9_ESM.zip › SD figure EV1-5/SD figure EV2/EV 2B/western FOXP1 INPUT.tif]

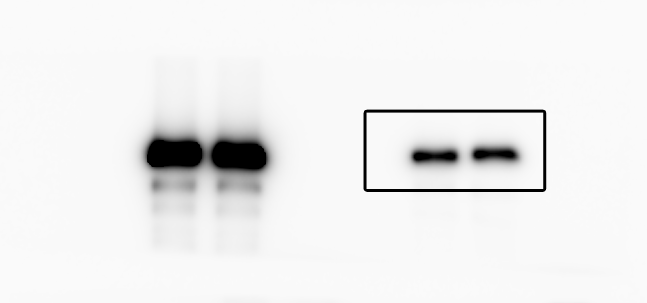

Supplement: Supplementary file 9 — Figure EV1-5 Source Data [file 44318_2024_323_MOESM9_ESM.zip › SD figure EV1-5/SD figure EV2/EV 2B/western FOXP1 pull down.tif]

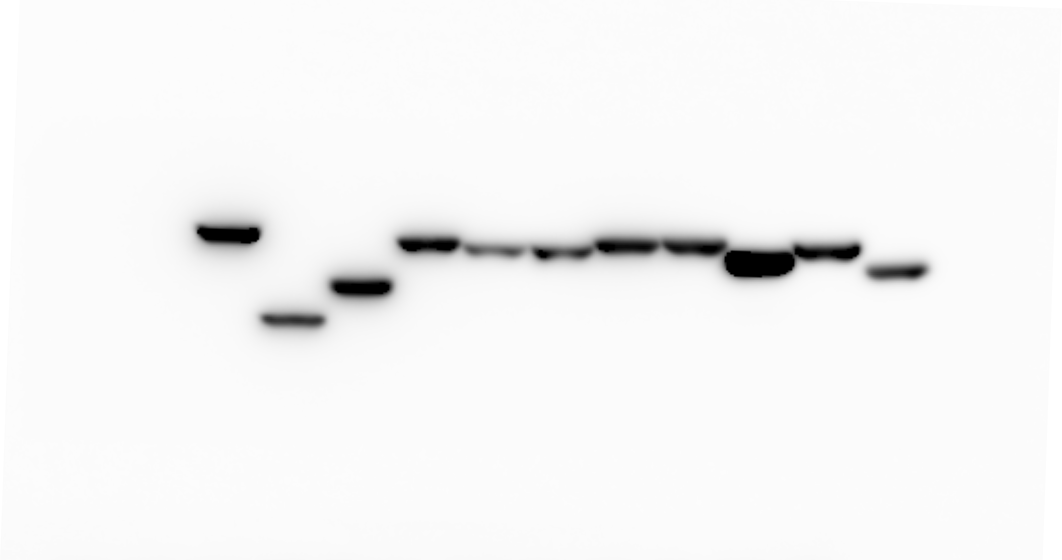

Supplement: Supplementary file 9 — Figure EV1-5 Source Data [file 44318_2024_323_MOESM9_ESM.zip › SD figure EV1-5/SD figure EV2/EV 2D/western FLAG INPUT.tif]

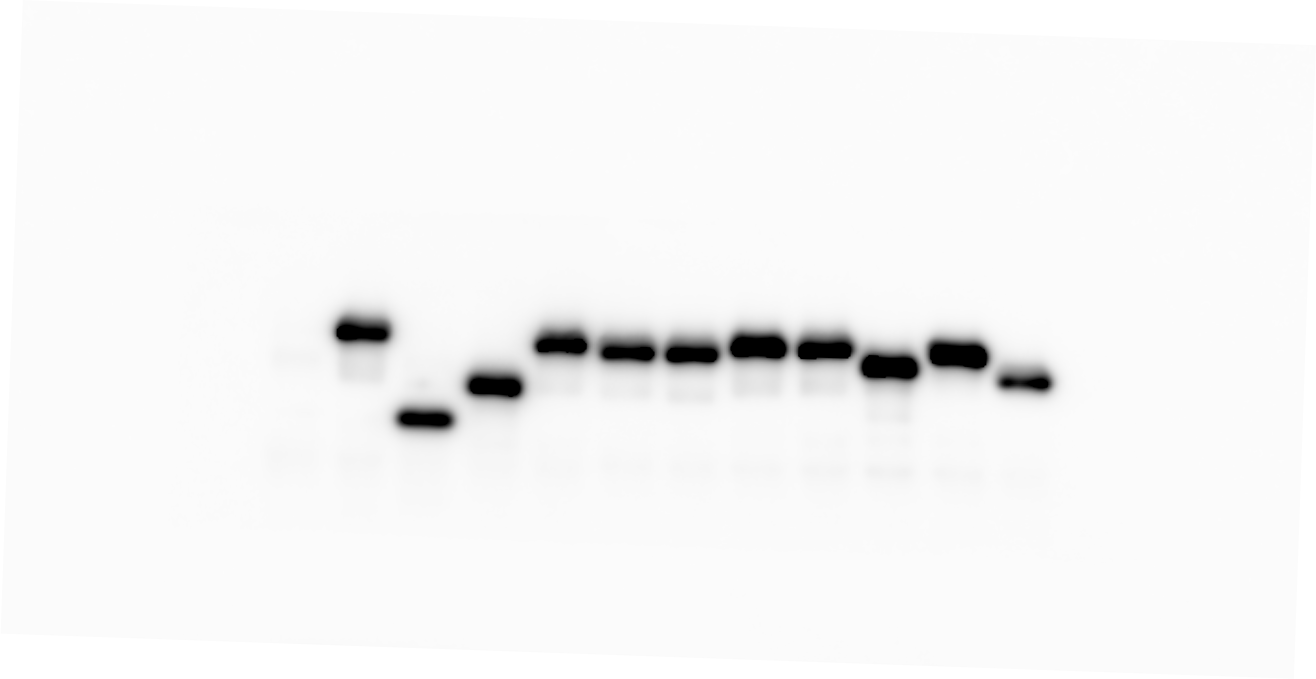

Supplement: Supplementary file 9 — Figure EV1-5 Source Data [file 44318_2024_323_MOESM9_ESM.zip › SD figure EV1-5/SD figure EV2/EV 2D/western FLAG IP.tif]

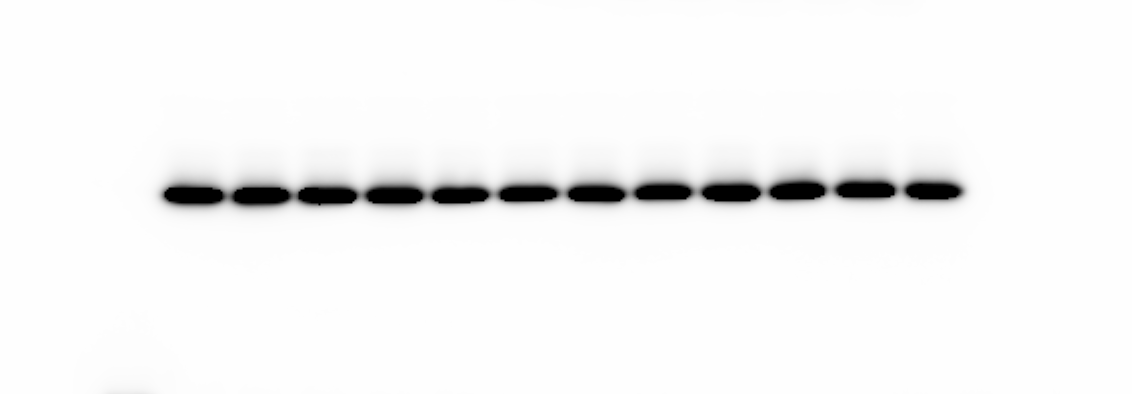

Supplement: Supplementary file 9 — Figure EV1-5 Source Data [file 44318_2024_323_MOESM9_ESM.zip › SD figure EV1-5/SD figure EV2/EV 2D/western RPA32 INPUT.tif]

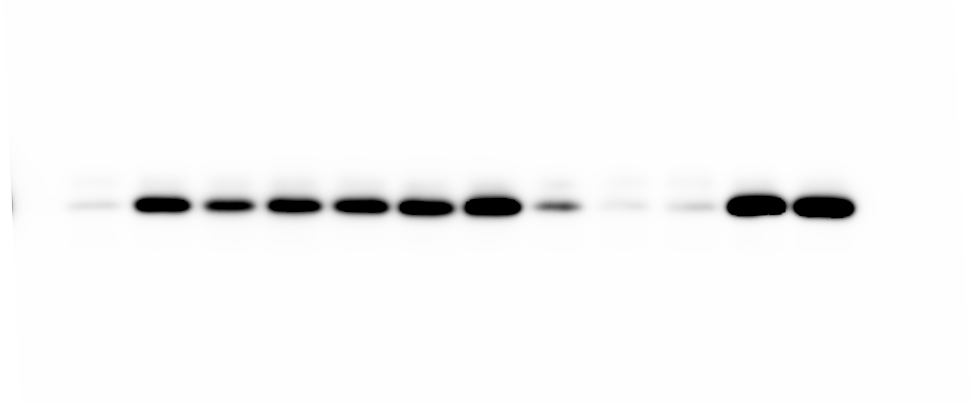

Supplement: Supplementary file 9 — Figure EV1-5 Source Data [file 44318_2024_323_MOESM9_ESM.zip › SD figure EV1-5/SD figure EV2/EV 2D/western RPA32 IP.tif]

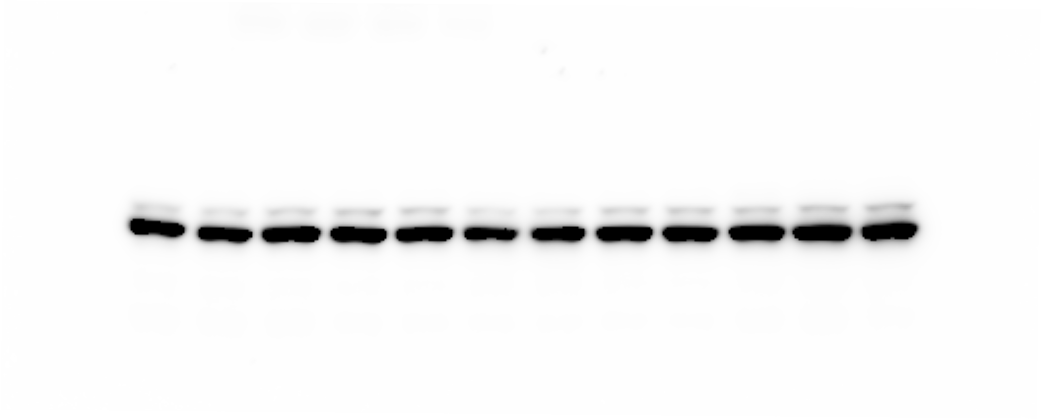

Supplement: Supplementary file 9 — Figure EV1-5 Source Data [file 44318_2024_323_MOESM9_ESM.zip › SD figure EV1-5/SD figure EV2/EV 2D/western RPA70 INPUT.tif]

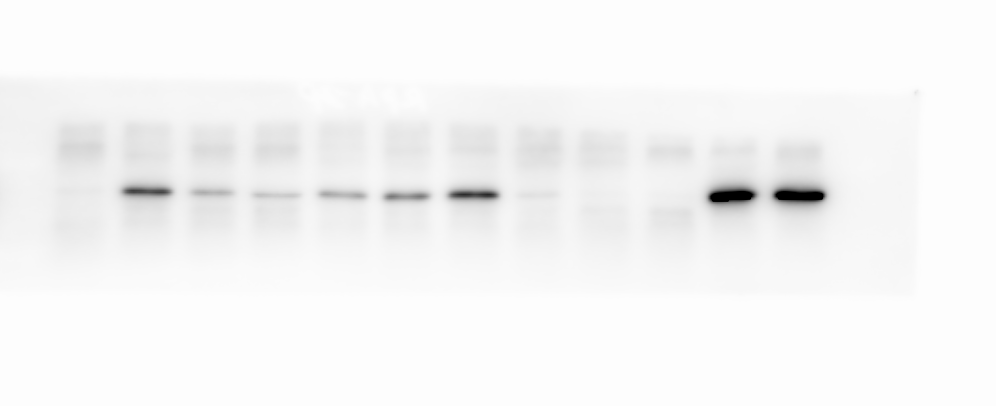

Supplement: Supplementary file 9 — Figure EV1-5 Source Data [file 44318_2024_323_MOESM9_ESM.zip › SD figure EV1-5/SD figure EV2/EV 2D/western RPA70 IP.tif]

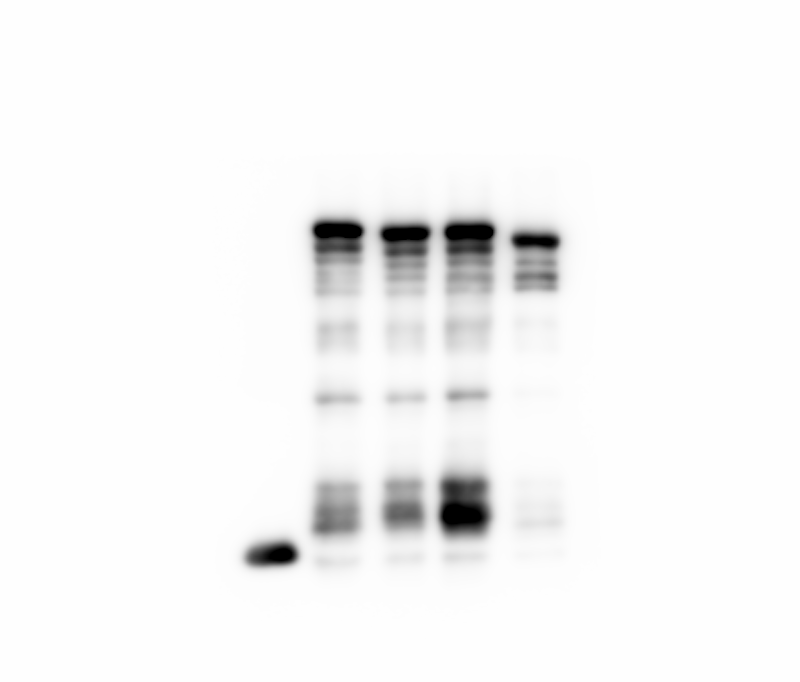

Supplement: Supplementary file 9 — Figure EV1-5 Source Data [file 44318_2024_323_MOESM9_ESM.zip › SD figure EV1-5/SD figure EV2/EV 2E/western GST.tif]

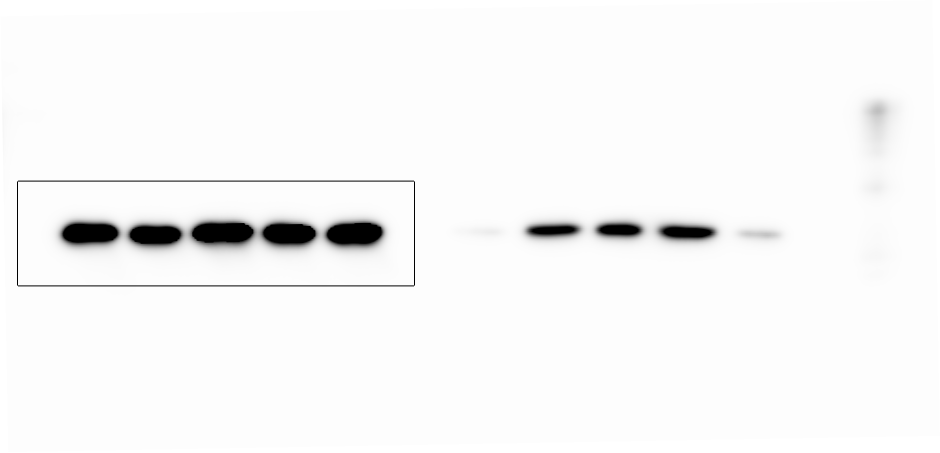

Supplement: Supplementary file 9 — Figure EV1-5 Source Data [file 44318_2024_323_MOESM9_ESM.zip › SD figure EV1-5/SD figure EV2/EV 2E/western His INPUT.tif]

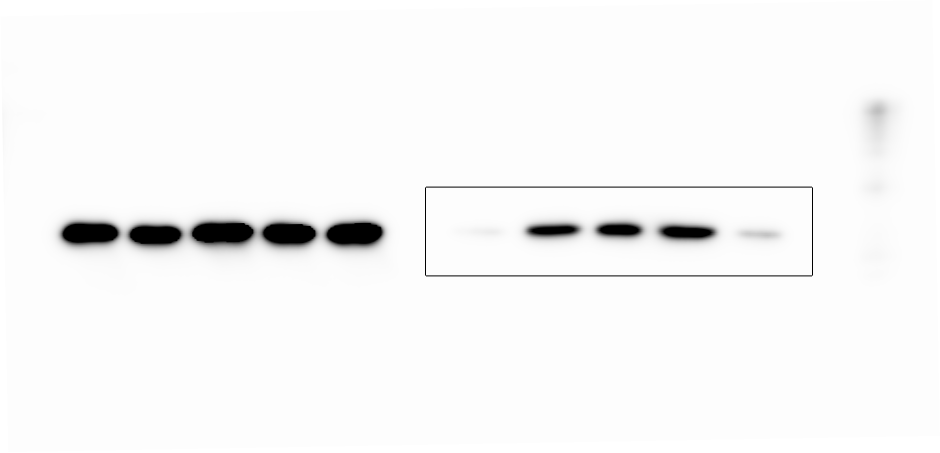

Supplement: Supplementary file 9 — Figure EV1-5 Source Data [file 44318_2024_323_MOESM9_ESM.zip › SD figure EV1-5/SD figure EV2/EV 2E/western His pulldown.tif]

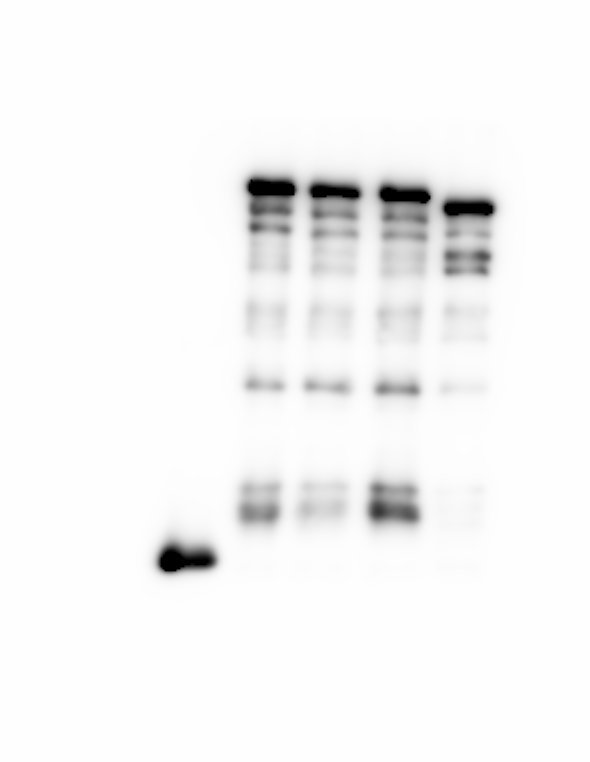

Supplement: Supplementary file 9 — Figure EV1-5 Source Data [file 44318_2024_323_MOESM9_ESM.zip › SD figure EV1-5/SD figure EV2/EV 2F/western GST.tif]

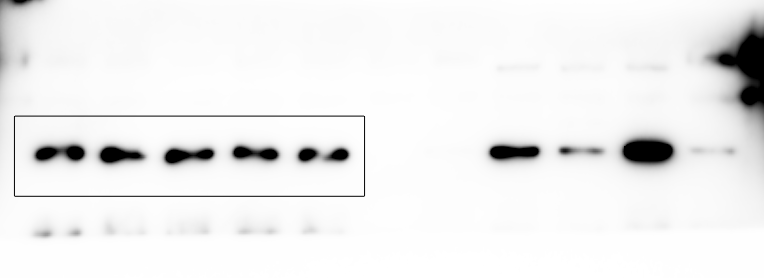

Supplement: Supplementary file 9 — Figure EV1-5 Source Data [file 44318_2024_323_MOESM9_ESM.zip › SD figure EV1-5/SD figure EV2/EV 2F/western His INPUT.tif]

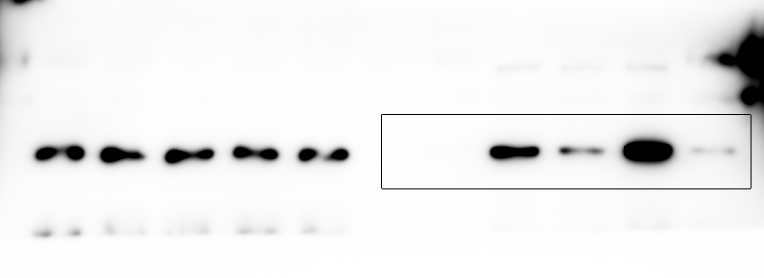

Supplement: Supplementary file 9 — Figure EV1-5 Source Data [file 44318_2024_323_MOESM9_ESM.zip › SD figure EV1-5/SD figure EV2/EV 2F/western His pulldown.tif]

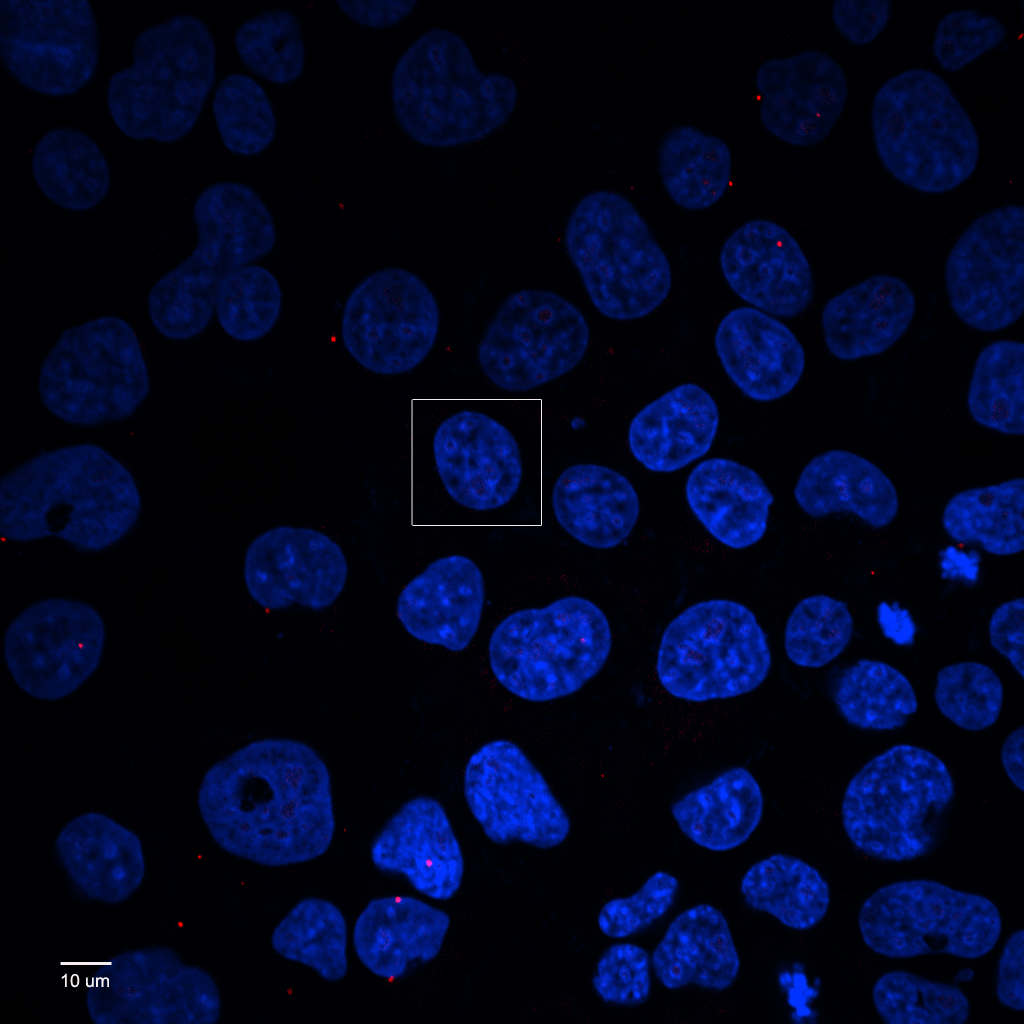

Supplement: Supplementary file 9 — Figure EV1-5 Source Data [file 44318_2024_323_MOESM9_ESM.zip › SD figure EV1-5/SD figure EV2/EV 2G/image siNC CON no click.tif]

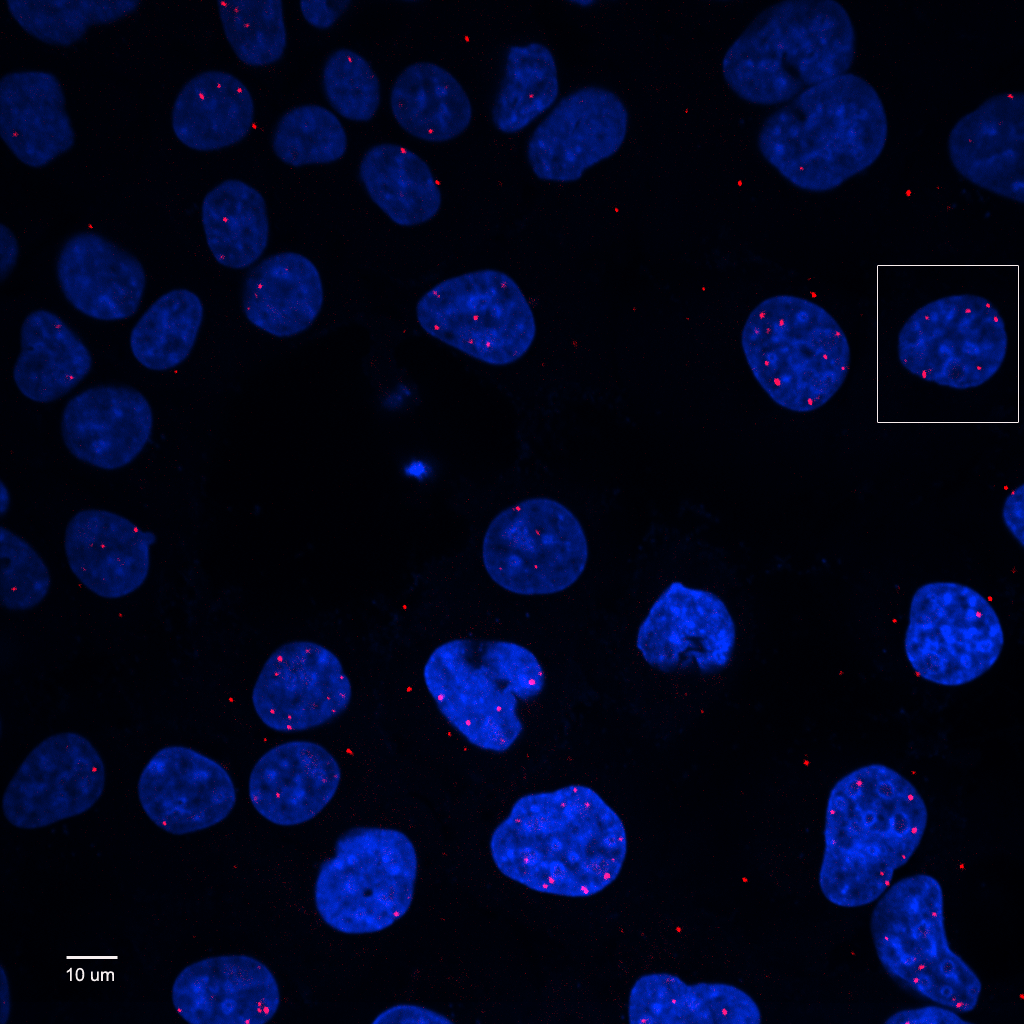

Supplement: Supplementary file 9 — Figure EV1-5 Source Data [file 44318_2024_323_MOESM9_ESM.zip › SD figure EV1-5/SD figure EV2/EV 2G/image siNC CON.tif]

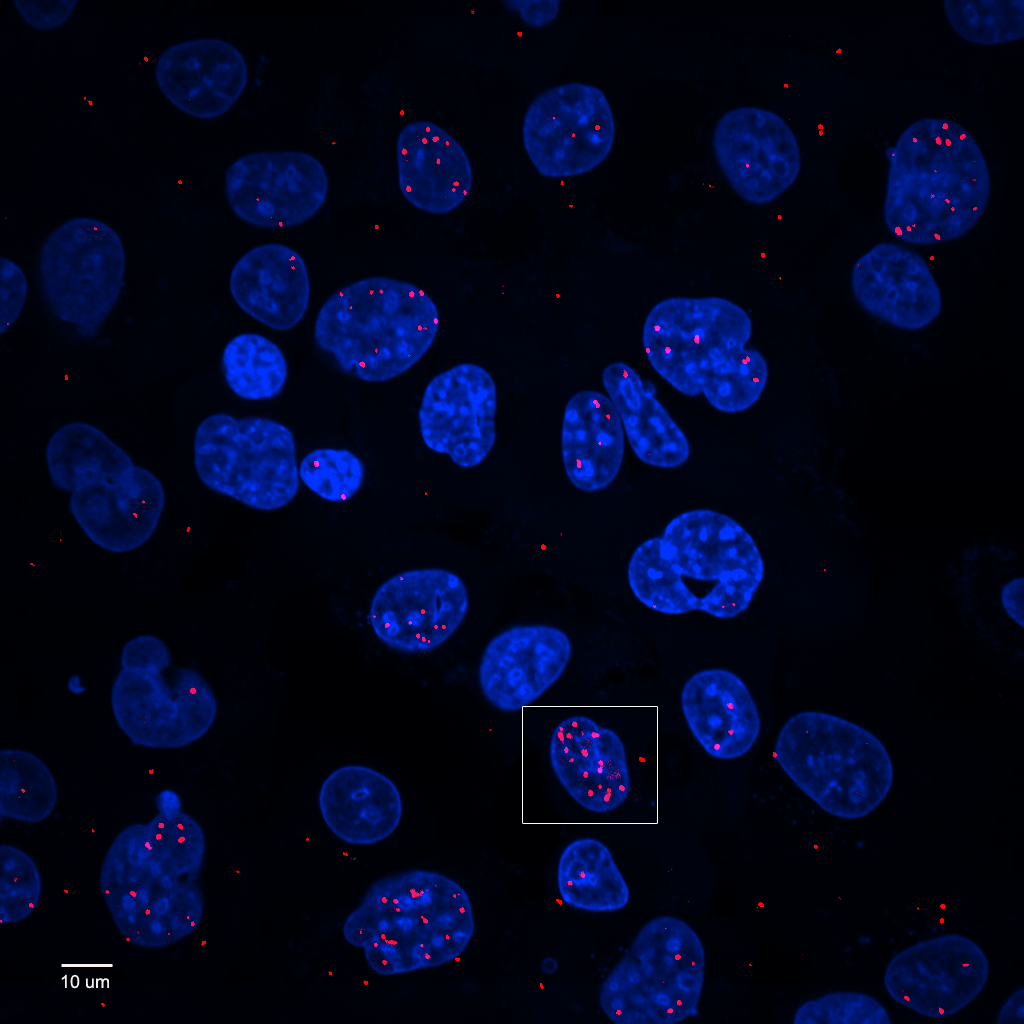

Supplement: Supplementary file 9 — Figure EV1-5 Source Data [file 44318_2024_323_MOESM9_ESM.zip › SD figure EV1-5/SD figure EV2/EV 2G/image siNC HU.tif]

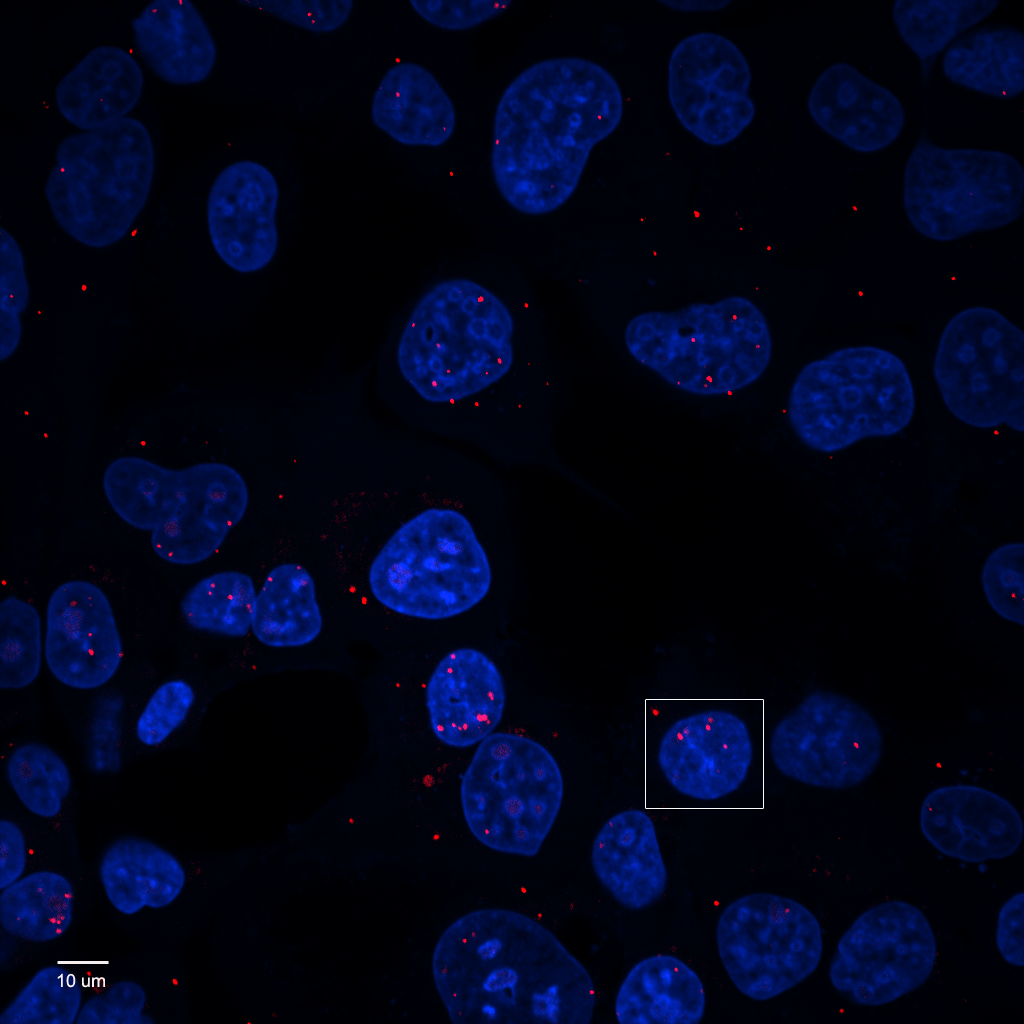

Supplement: Supplementary file 9 — Figure EV1-5 Source Data [file 44318_2024_323_MOESM9_ESM.zip › SD figure EV1-5/SD figure EV2/EV 2G/image siRPA32 CON.tif]

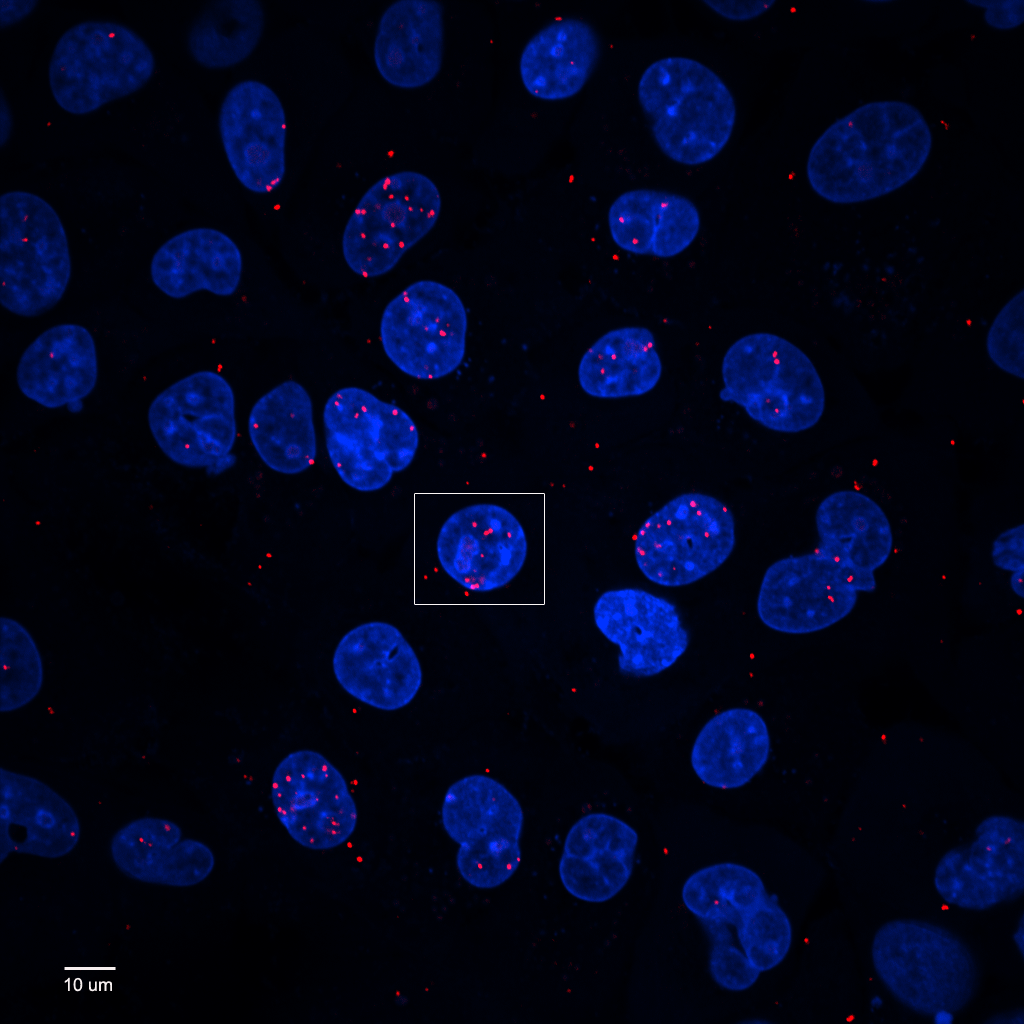

Supplement: Supplementary file 9 — Figure EV1-5 Source Data [file 44318_2024_323_MOESM9_ESM.zip › SD figure EV1-5/SD figure EV2/EV 2G/image siRPA32 HU.tif]

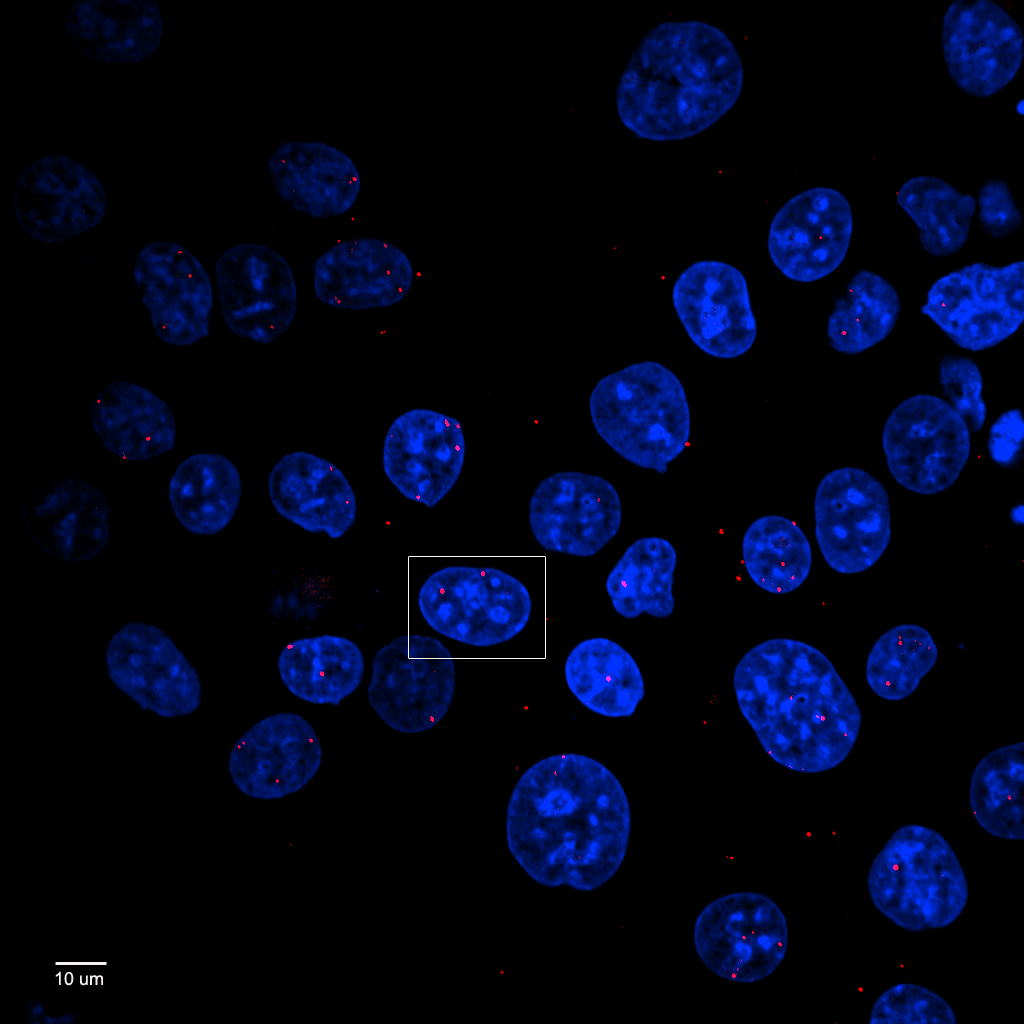

Supplement: Supplementary file 9 — Figure EV1-5 Source Data [file 44318_2024_323_MOESM9_ESM.zip › SD figure EV1-5/SD figure EV2/EV 2G/image siRPA70 CON.tif]

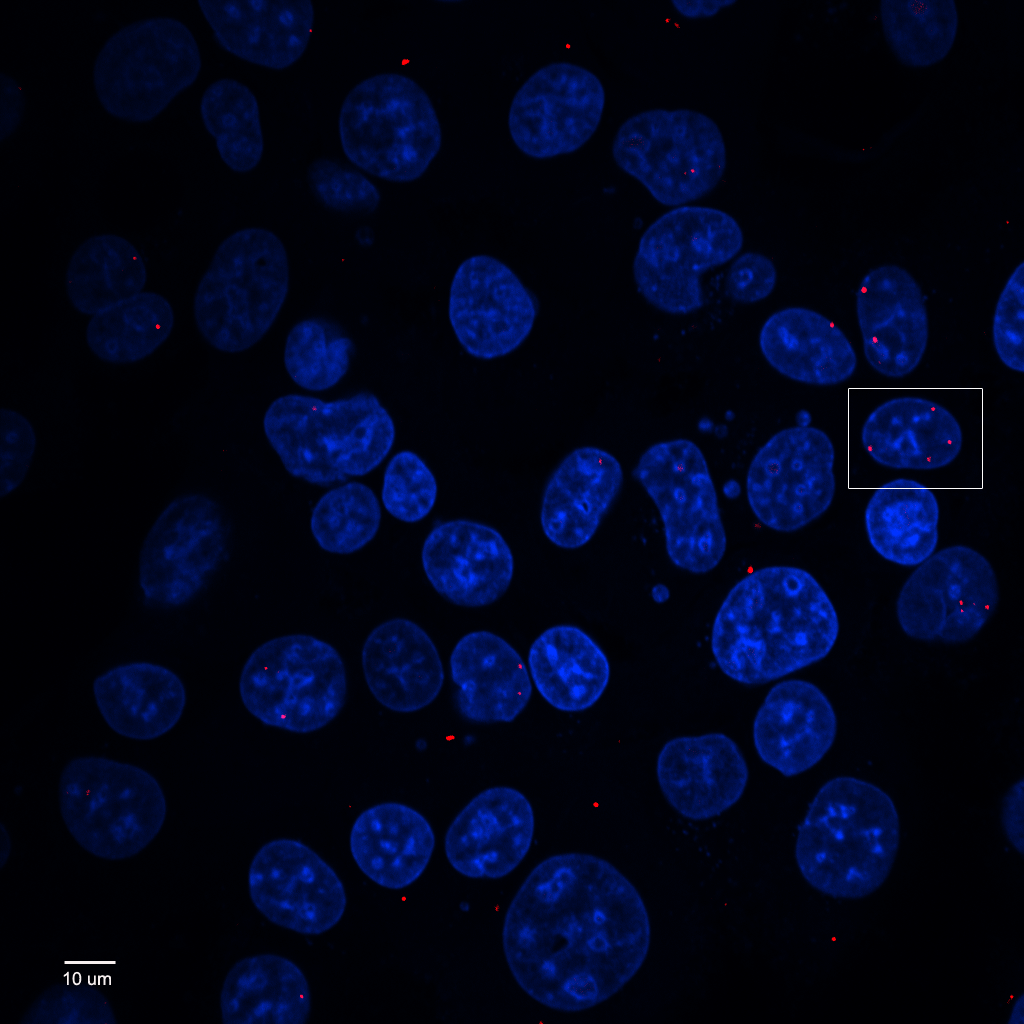

Supplement: Supplementary file 9 — Figure EV1-5 Source Data [file 44318_2024_323_MOESM9_ESM.zip › SD figure EV1-5/SD figure EV2/EV 2G/image siRPA70 HU.tif]

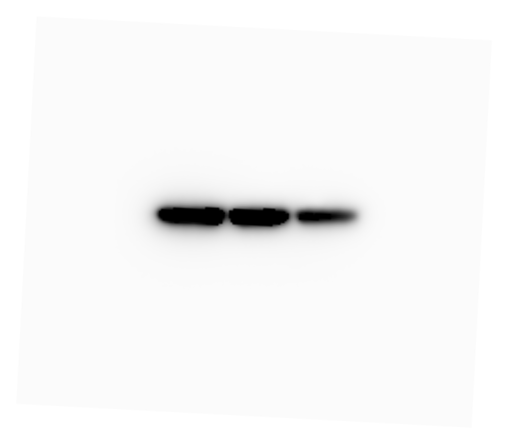

Supplement: Supplementary file 9 — Figure EV1-5 Source Data [file 44318_2024_323_MOESM9_ESM.zip › SD figure EV1-5/SD figure EV2/EV 2G/western Actin.tif]
