# Supplementary figures and images for: FOXP1 phosphorylation antagonizes its O-GlcNAcylation in regulating ATR activation in response to replication stress (part 3 of 3)
Source: EMBO J. 2024 Dec 2;44(2):457–83. doi: 10.1038/s44318-024-00323-x (PMC11729909; doi:10.1038/s44318-024-00323-x)

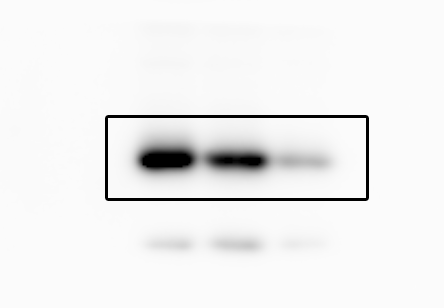

Supplement: Supplementary file 9 — Figure EV1-5 Source Data [file 44318_2024_323_MOESM9_ESM.zip › SD figure EV1-5/SD figure EV2/EV 2G/western RPA32.tif]

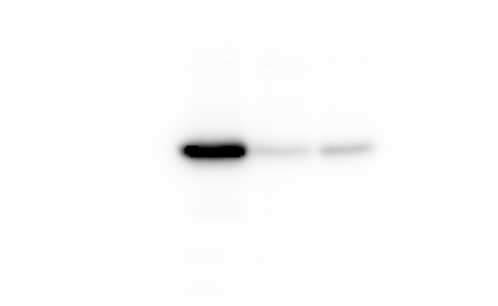

Supplement: Supplementary file 9 — Figure EV1-5 Source Data [file 44318_2024_323_MOESM9_ESM.zip › SD figure EV1-5/SD figure EV2/EV 2G/western RPA70.tif]

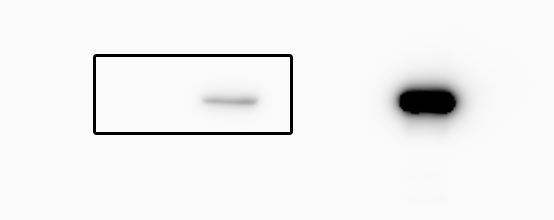

Supplement: Supplementary file 9 — Figure EV1-5 Source Data [file 44318_2024_323_MOESM9_ESM.zip › SD figure EV1-5/SD figure EV3/EV 3A/western FLAG INPUT.tif]

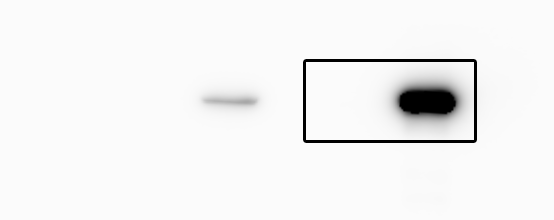

Supplement: Supplementary file 9 — Figure EV1-5 Source Data [file 44318_2024_323_MOESM9_ESM.zip › SD figure EV1-5/SD figure EV3/EV 3A/western FLAG IP.tif]

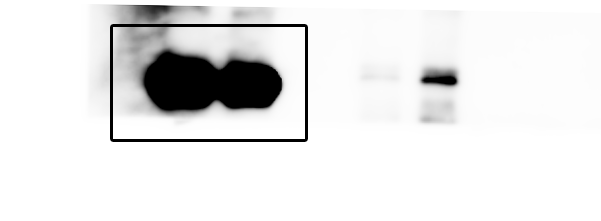

Supplement: Supplementary file 9 — Figure EV1-5 Source Data [file 44318_2024_323_MOESM9_ESM.zip › SD figure EV1-5/SD figure EV3/EV 3A/western OGT INPUT.tif]

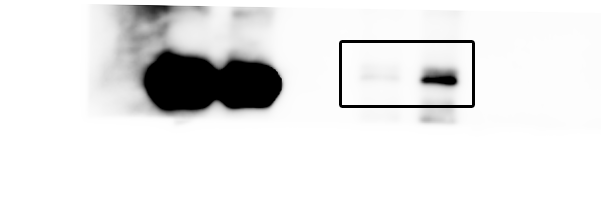

Supplement: Supplementary file 9 — Figure EV1-5 Source Data [file 44318_2024_323_MOESM9_ESM.zip › SD figure EV1-5/SD figure EV3/EV 3A/western OGT IP.tif]

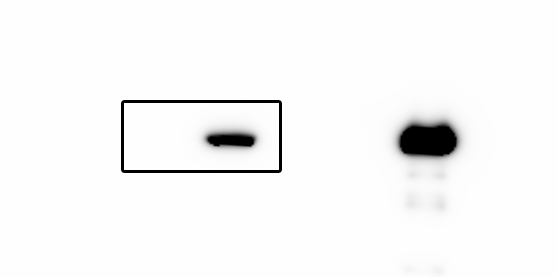

Supplement: Supplementary file 9 — Figure EV1-5 Source Data [file 44318_2024_323_MOESM9_ESM.zip › SD figure EV1-5/SD figure EV3/EV 3B/western FLAG INPUT.tif]

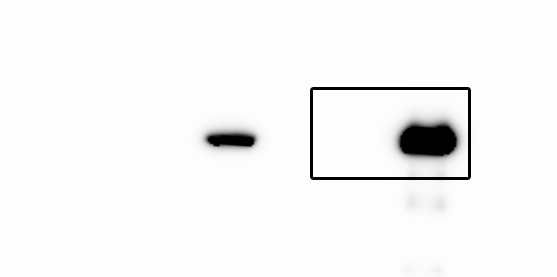

Supplement: Supplementary file 9 — Figure EV1-5 Source Data [file 44318_2024_323_MOESM9_ESM.zip › SD figure EV1-5/SD figure EV3/EV 3B/western FLAG IP.tif]

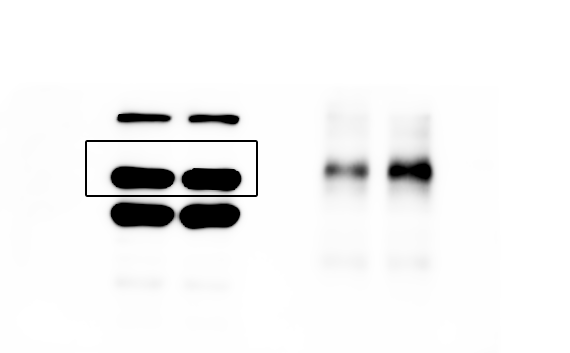

Supplement: Supplementary file 9 — Figure EV1-5 Source Data [file 44318_2024_323_MOESM9_ESM.zip › SD figure EV1-5/SD figure EV3/EV 3B/western FOXP1 INPUT.tif]

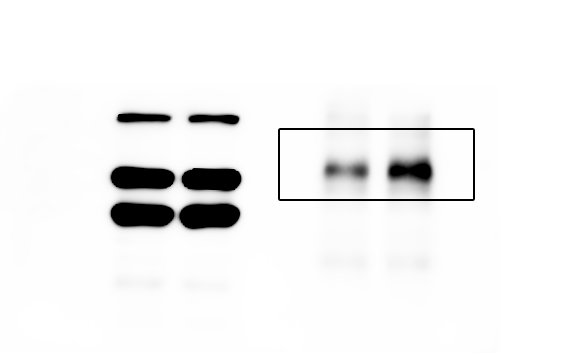

Supplement: Supplementary file 9 — Figure EV1-5 Source Data [file 44318_2024_323_MOESM9_ESM.zip › SD figure EV1-5/SD figure EV3/EV 3B/western FOXP1 IP.tif]

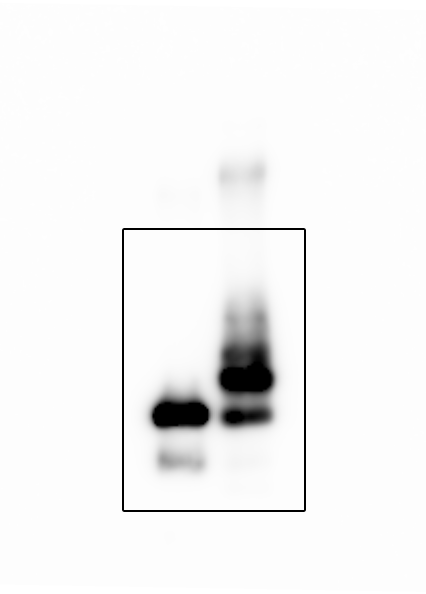

Supplement: Supplementary file 9 — Figure EV1-5 Source Data [file 44318_2024_323_MOESM9_ESM.zip › SD figure EV1-5/SD figure EV3/EV 3C/western FLAG.tif]

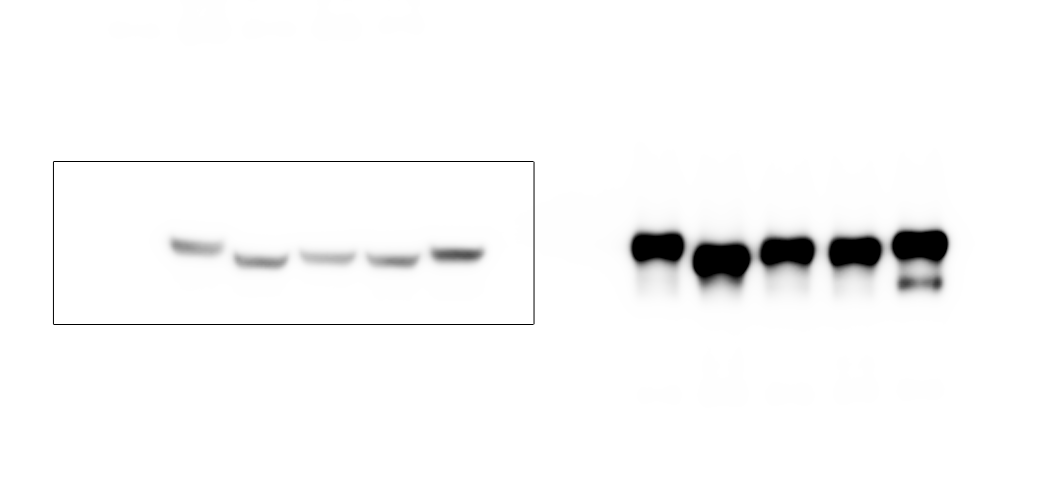

Supplement: Supplementary file 9 — Figure EV1-5 Source Data [file 44318_2024_323_MOESM9_ESM.zip › SD figure EV1-5/SD figure EV3/EV 3D/western FLAG INPUT.tif]

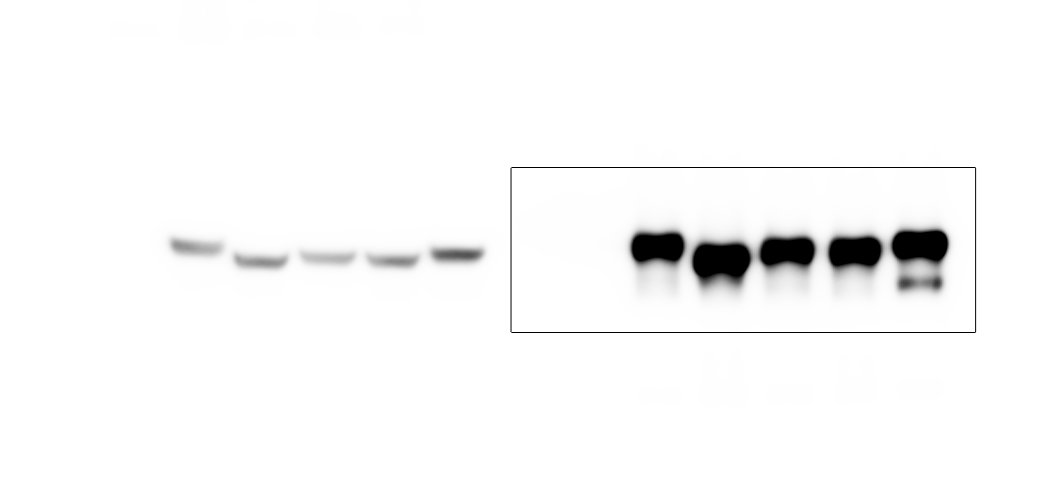

Supplement: Supplementary file 9 — Figure EV1-5 Source Data [file 44318_2024_323_MOESM9_ESM.zip › SD figure EV1-5/SD figure EV3/EV 3D/western FLAG IP.tif]

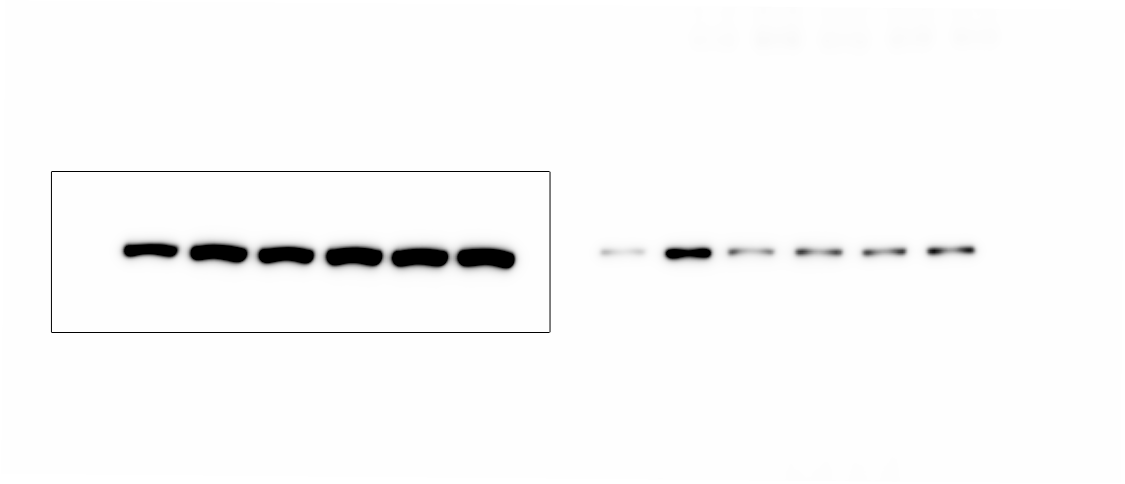

Supplement: Supplementary file 9 — Figure EV1-5 Source Data [file 44318_2024_323_MOESM9_ESM.zip › SD figure EV1-5/SD figure EV3/EV 3D/western GFP INPUT.tif]

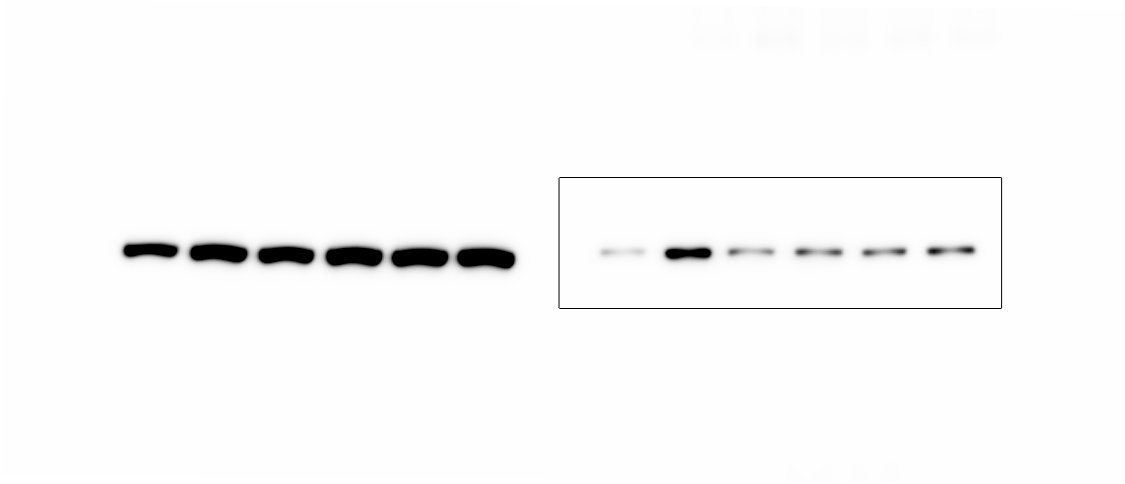

Supplement: Supplementary file 9 — Figure EV1-5 Source Data [file 44318_2024_323_MOESM9_ESM.zip › SD figure EV1-5/SD figure EV3/EV 3D/western GFP IP.tif]

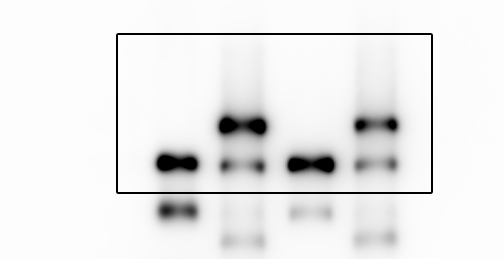

Supplement: Supplementary file 9 — Figure EV1-5 Source Data [file 44318_2024_323_MOESM9_ESM.zip › SD figure EV1-5/SD figure EV3/EV 3E/western FLAG.tif]

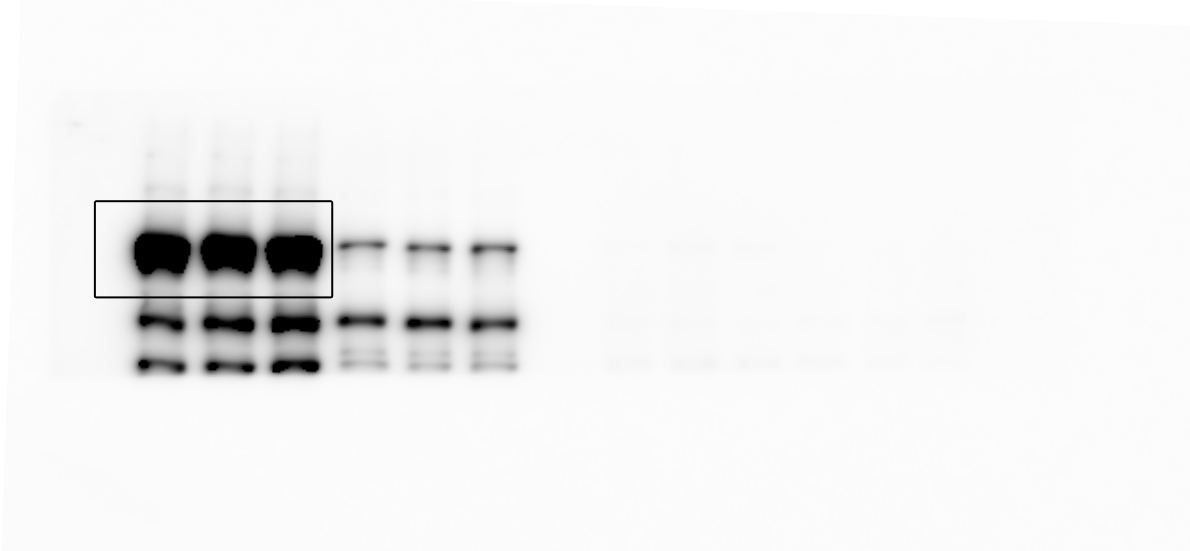

Supplement: Supplementary file 9 — Figure EV1-5 Source Data [file 44318_2024_323_MOESM9_ESM.zip › SD figure EV1-5/SD figure EV3/EV 3F/western ATR INPUT.tif]

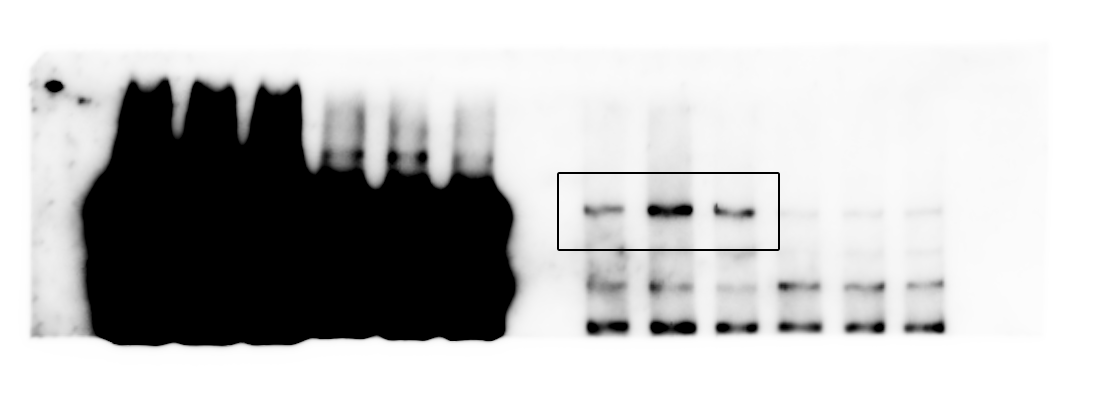

Supplement: Supplementary file 9 — Figure EV1-5 Source Data [file 44318_2024_323_MOESM9_ESM.zip › SD figure EV1-5/SD figure EV3/EV 3F/western ATR IP.tif]

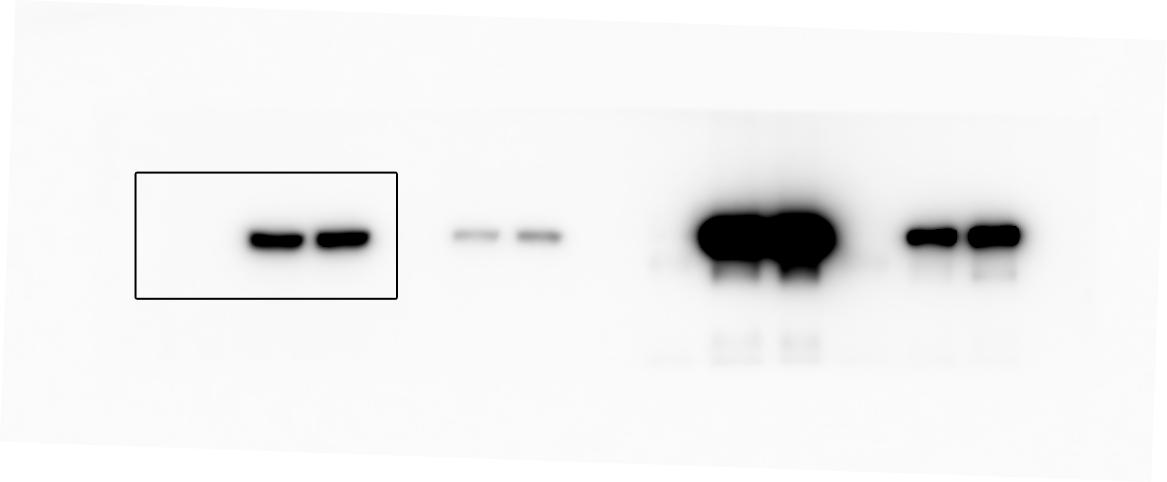

Supplement: Supplementary file 9 — Figure EV1-5 Source Data [file 44318_2024_323_MOESM9_ESM.zip › SD figure EV1-5/SD figure EV3/EV 3F/western FLAG INPUT.tif]

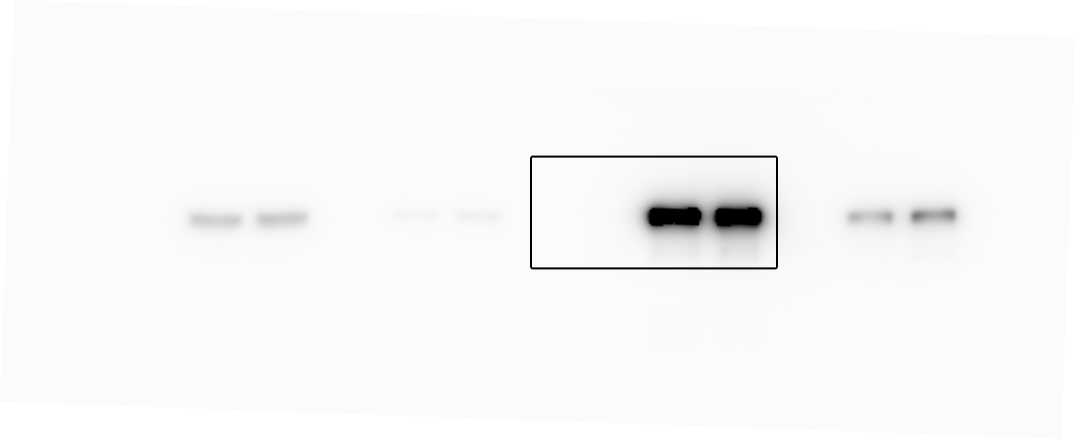

Supplement: Supplementary file 9 — Figure EV1-5 Source Data [file 44318_2024_323_MOESM9_ESM.zip › SD figure EV1-5/SD figure EV3/EV 3F/western FLAG IP.tif]

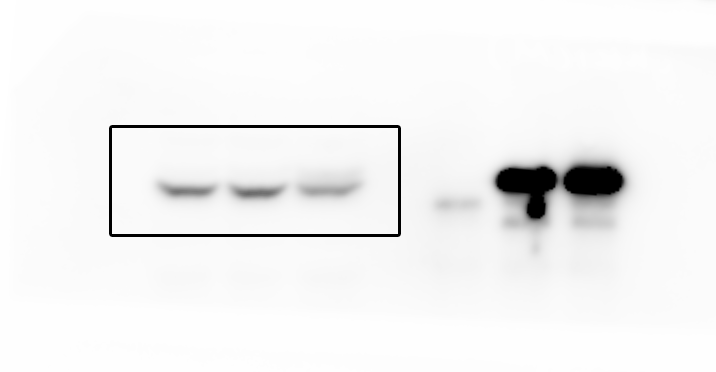

Supplement: Supplementary file 9 — Figure EV1-5 Source Data [file 44318_2024_323_MOESM9_ESM.zip › SD figure EV1-5/SD figure EV4/EV 4A/western CHK1 INPUT.tif]

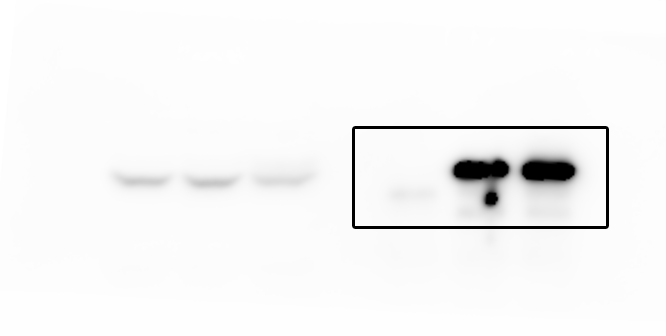

Supplement: Supplementary file 9 — Figure EV1-5 Source Data [file 44318_2024_323_MOESM9_ESM.zip › SD figure EV1-5/SD figure EV4/EV 4A/western CHK1 IP.tif]

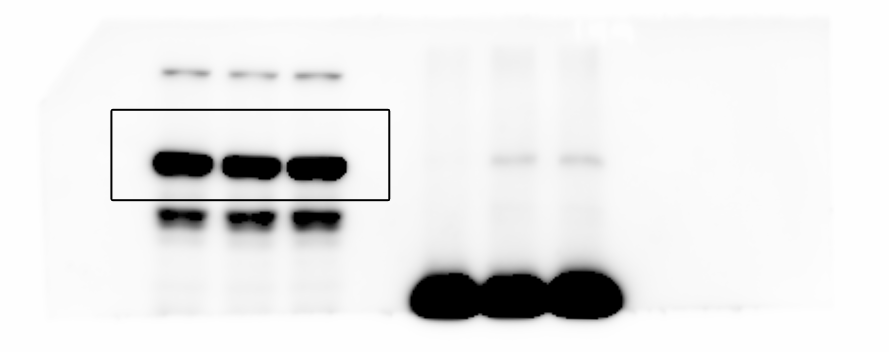

Supplement: Supplementary file 9 — Figure EV1-5 Source Data [file 44318_2024_323_MOESM9_ESM.zip › SD figure EV1-5/SD figure EV4/EV 4A/western FOXP1 INPUT.tif]

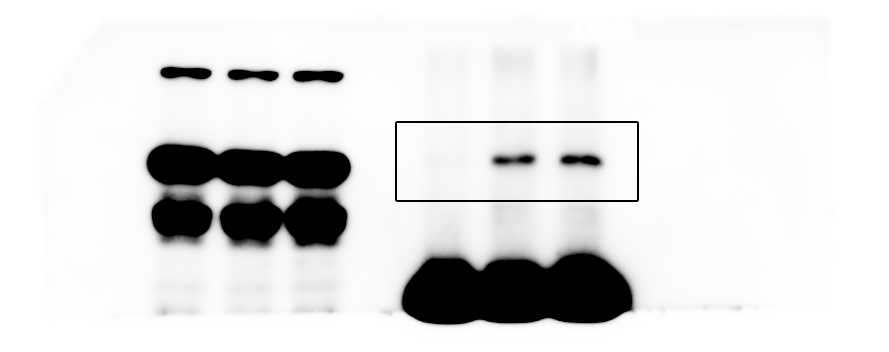

Supplement: Supplementary file 9 — Figure EV1-5 Source Data [file 44318_2024_323_MOESM9_ESM.zip › SD figure EV1-5/SD figure EV4/EV 4A/western FOXP1 IP.tif]

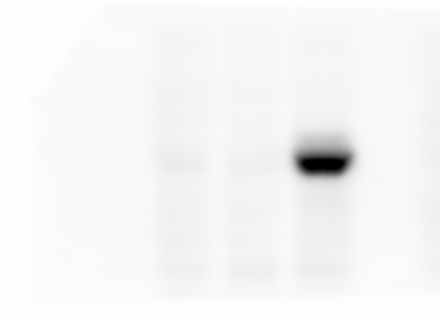

Supplement: Supplementary file 9 — Figure EV1-5 Source Data [file 44318_2024_323_MOESM9_ESM.zip › SD figure EV1-5/SD figure EV4/EV 4A/western pCHK1S345.tif]

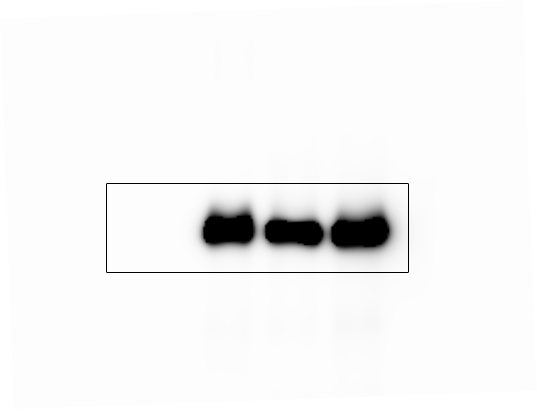

Supplement: Supplementary file 9 — Figure EV1-5 Source Data [file 44318_2024_323_MOESM9_ESM.zip › SD figure EV1-5/SD figure EV4/EV 4C/western CHK1.tif]

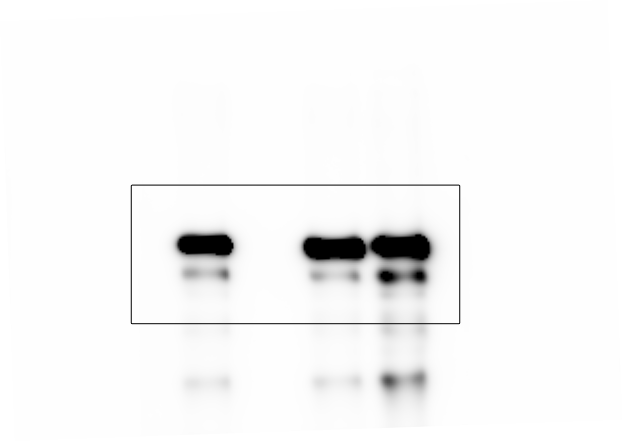

Supplement: Supplementary file 9 — Figure EV1-5 Source Data [file 44318_2024_323_MOESM9_ESM.zip › SD figure EV1-5/SD figure EV4/EV 4C/western FOXP1.tif]

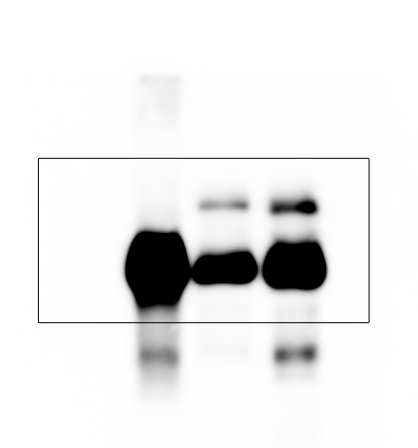

Supplement: Supplementary file 9 — Figure EV1-5 Source Data [file 44318_2024_323_MOESM9_ESM.zip › SD figure EV1-5/SD figure EV4/EV 4C/western Thiophosphate ester.tif]

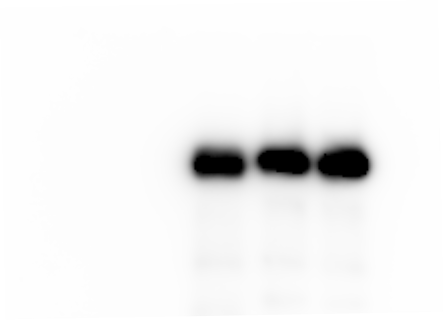

Supplement: Supplementary file 9 — Figure EV1-5 Source Data [file 44318_2024_323_MOESM9_ESM.zip › SD figure EV1-5/SD figure EV4/EV 4D/western CHK1.tif]

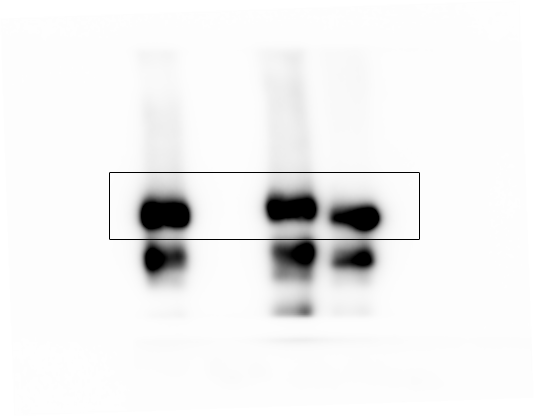

Supplement: Supplementary file 9 — Figure EV1-5 Source Data [file 44318_2024_323_MOESM9_ESM.zip › SD figure EV1-5/SD figure EV4/EV 4D/western FOXP1.tif]

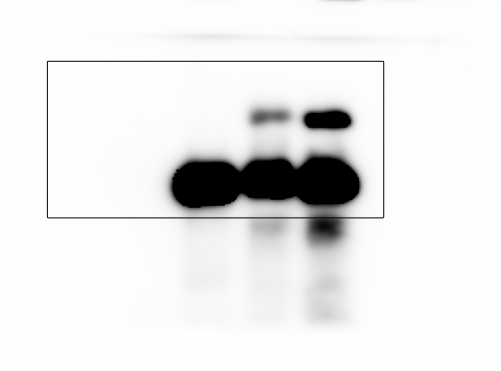

Supplement: Supplementary file 9 — Figure EV1-5 Source Data [file 44318_2024_323_MOESM9_ESM.zip › SD figure EV1-5/SD figure EV4/EV 4D/western Thiophosphate ester.tif]

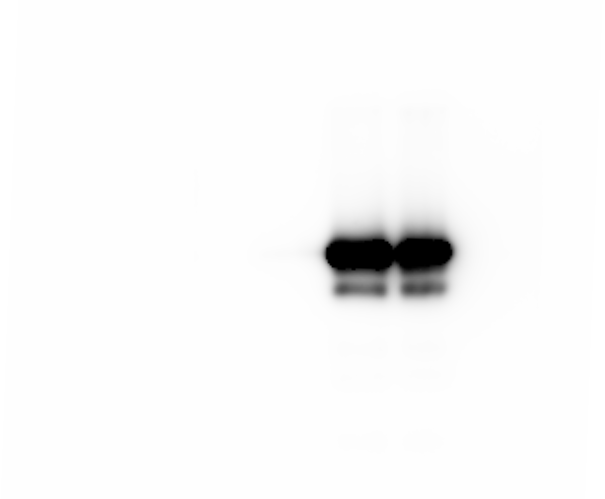

Supplement: Supplementary file 9 — Figure EV1-5 Source Data [file 44318_2024_323_MOESM9_ESM.zip › SD figure EV1-5/SD figure EV4/EV 4E/western FLAG.tif]

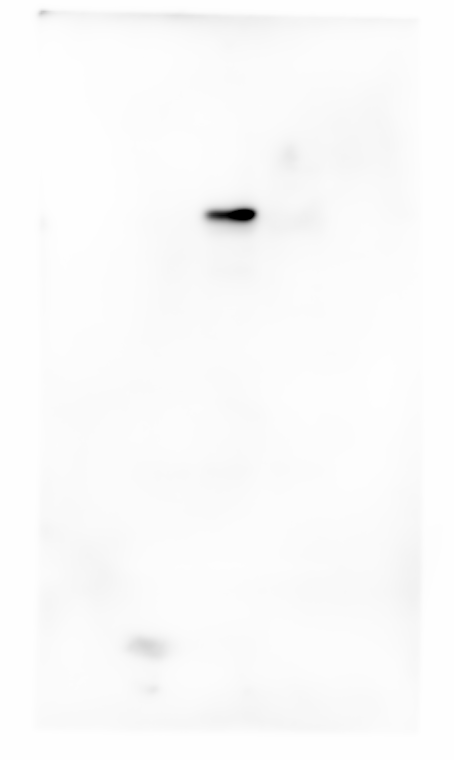

Supplement: Supplementary file 9 — Figure EV1-5 Source Data [file 44318_2024_323_MOESM9_ESM.zip › SD figure EV1-5/SD figure EV4/EV 4E/western pFOXP1S396.tif]

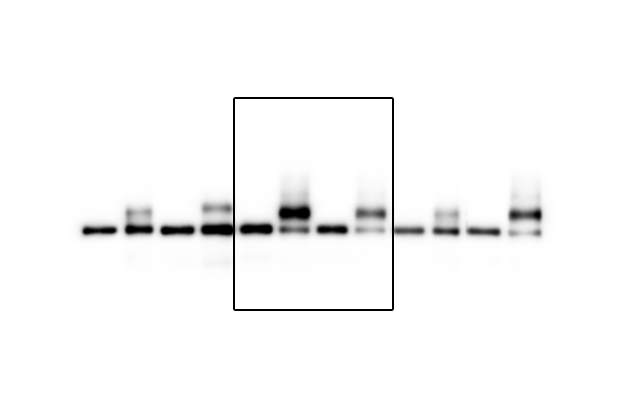

Supplement: Supplementary file 9 — Figure EV1-5 Source Data [file 44318_2024_323_MOESM9_ESM.zip › SD figure EV1-5/SD figure EV4/EV 4F/western FLAG.tif]

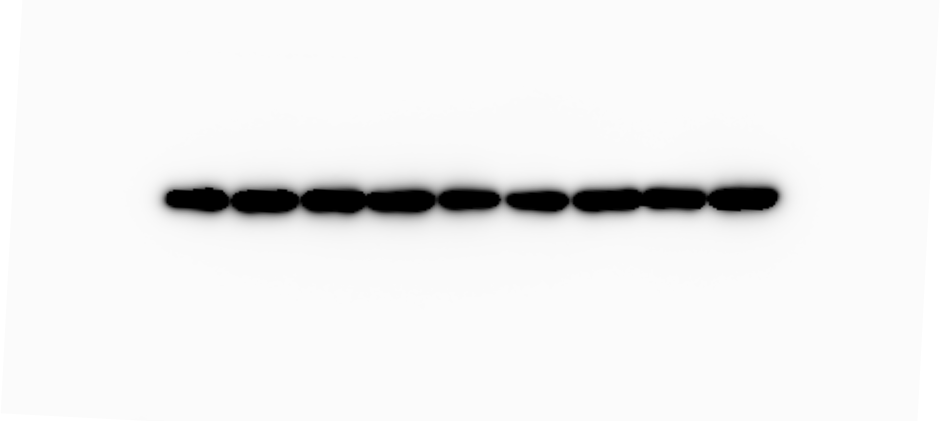

Supplement: Supplementary file 9 — Figure EV1-5 Source Data [file 44318_2024_323_MOESM9_ESM.zip › SD figure EV1-5/SD figure EV4/EV 4G/western Actin.tif]

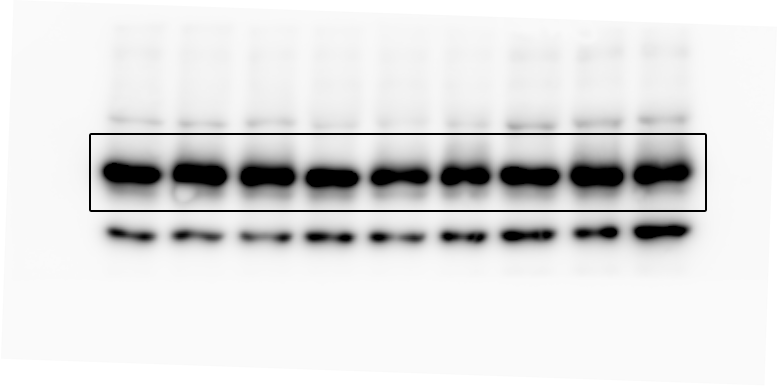

Supplement: Supplementary file 9 — Figure EV1-5 Source Data [file 44318_2024_323_MOESM9_ESM.zip › SD figure EV1-5/SD figure EV4/EV 4G/western CHK1.tif]

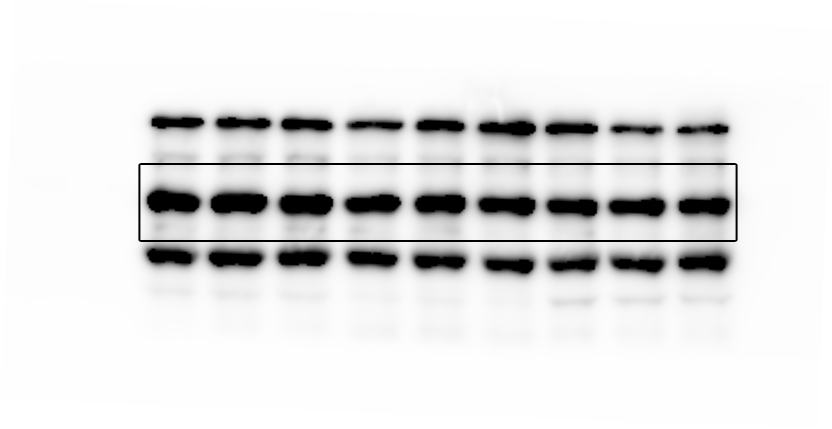

Supplement: Supplementary file 9 — Figure EV1-5 Source Data [file 44318_2024_323_MOESM9_ESM.zip › SD figure EV1-5/SD figure EV4/EV 4G/western FOXP1.tif]

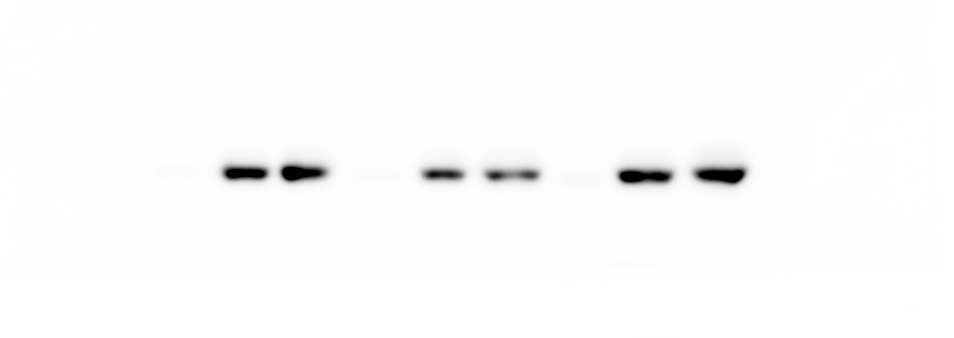

Supplement: Supplementary file 9 — Figure EV1-5 Source Data [file 44318_2024_323_MOESM9_ESM.zip › SD figure EV1-5/SD figure EV4/EV 4G/western pCHK1S345.tif]

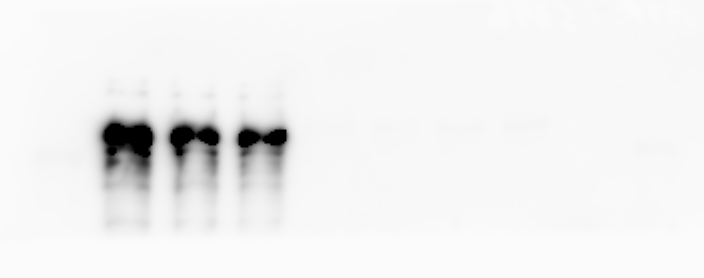

Supplement: Supplementary file 9 — Figure EV1-5 Source Data [file 44318_2024_323_MOESM9_ESM.zip › SD figure EV1-5/SD figure EV4/EV 4H/western ATR INPUT.tif]

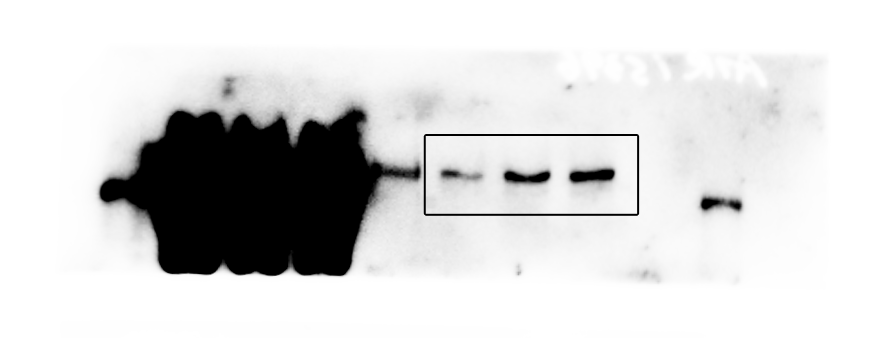

Supplement: Supplementary file 9 — Figure EV1-5 Source Data [file 44318_2024_323_MOESM9_ESM.zip › SD figure EV1-5/SD figure EV4/EV 4H/western ATR pull-down.tif]

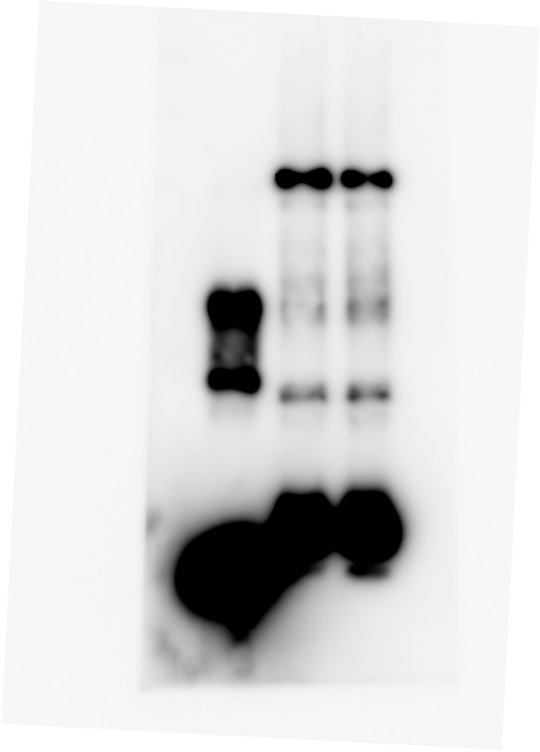

Supplement: Supplementary file 9 — Figure EV1-5 Source Data [file 44318_2024_323_MOESM9_ESM.zip › SD figure EV1-5/SD figure EV4/EV 4H/western GST.tif]

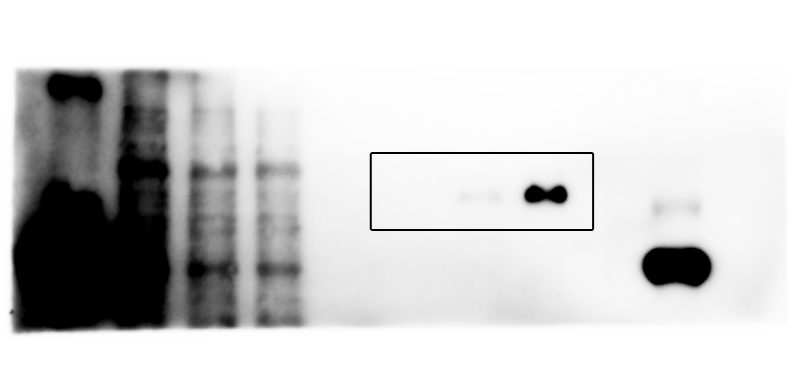

Supplement: Supplementary file 9 — Figure EV1-5 Source Data [file 44318_2024_323_MOESM9_ESM.zip › SD figure EV1-5/SD figure EV4/EV 4H/western pFOXP1S396.tif]

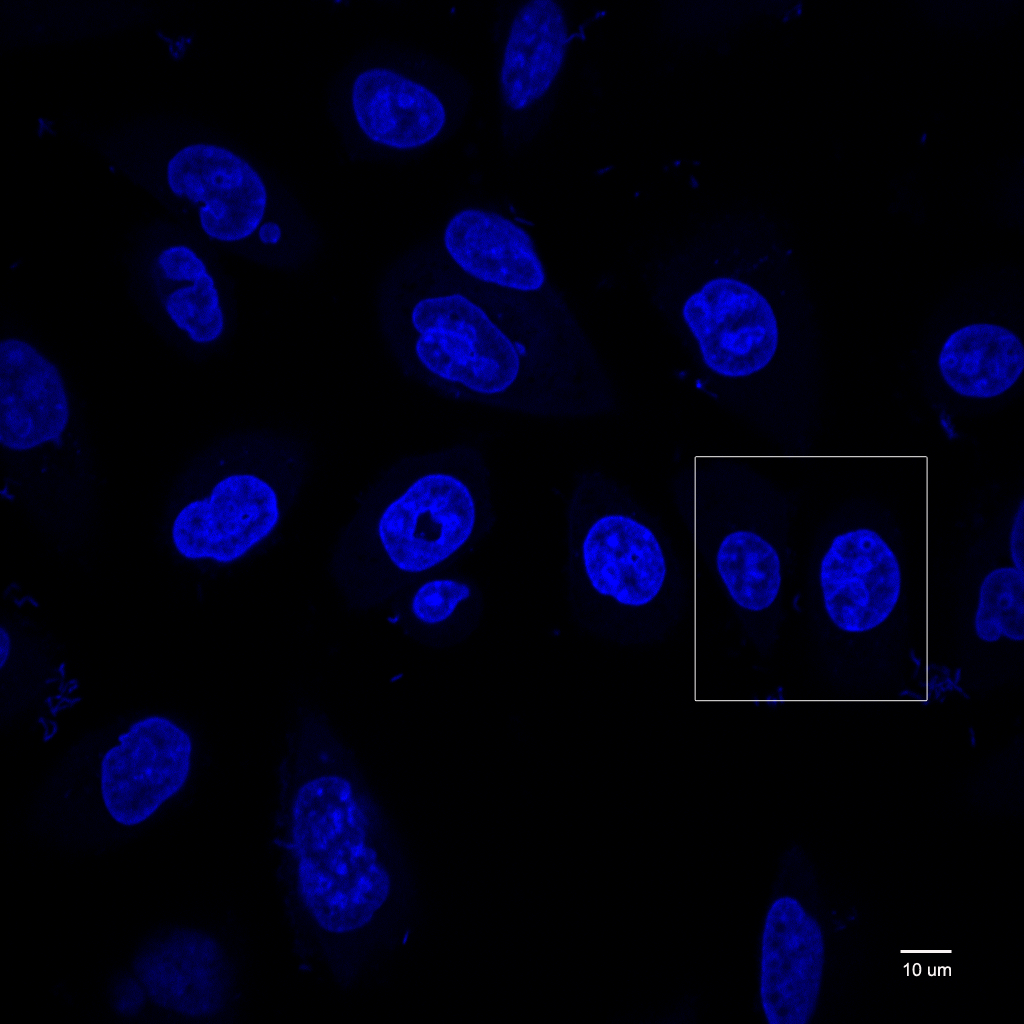

Supplement: Supplementary file 9 — Figure EV1-5 Source Data [file 44318_2024_323_MOESM9_ESM.zip › SD figure EV1-5/SD figure EV5/EV 5A/image R465G DAPI.tif]

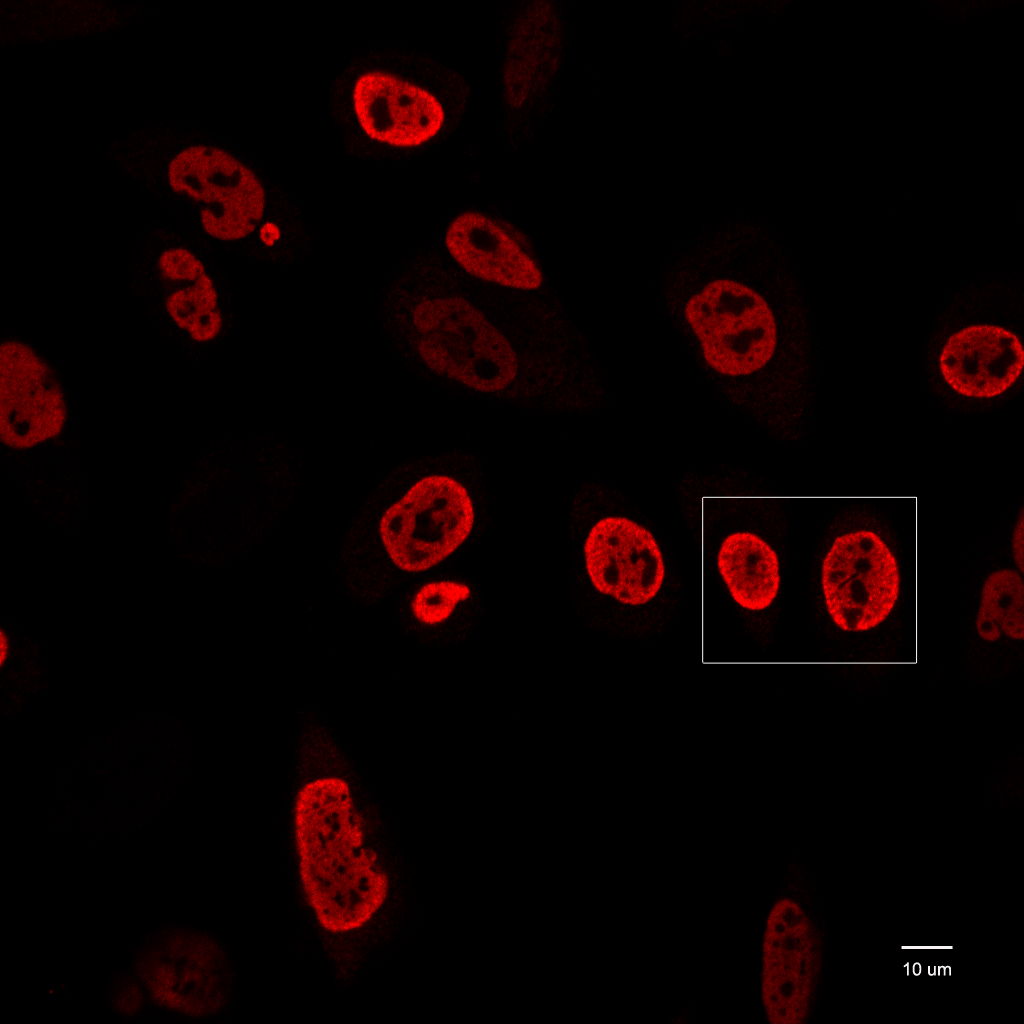

Supplement: Supplementary file 9 — Figure EV1-5 Source Data [file 44318_2024_323_MOESM9_ESM.zip › SD figure EV1-5/SD figure EV5/EV 5A/image R465G FLAG.tif]

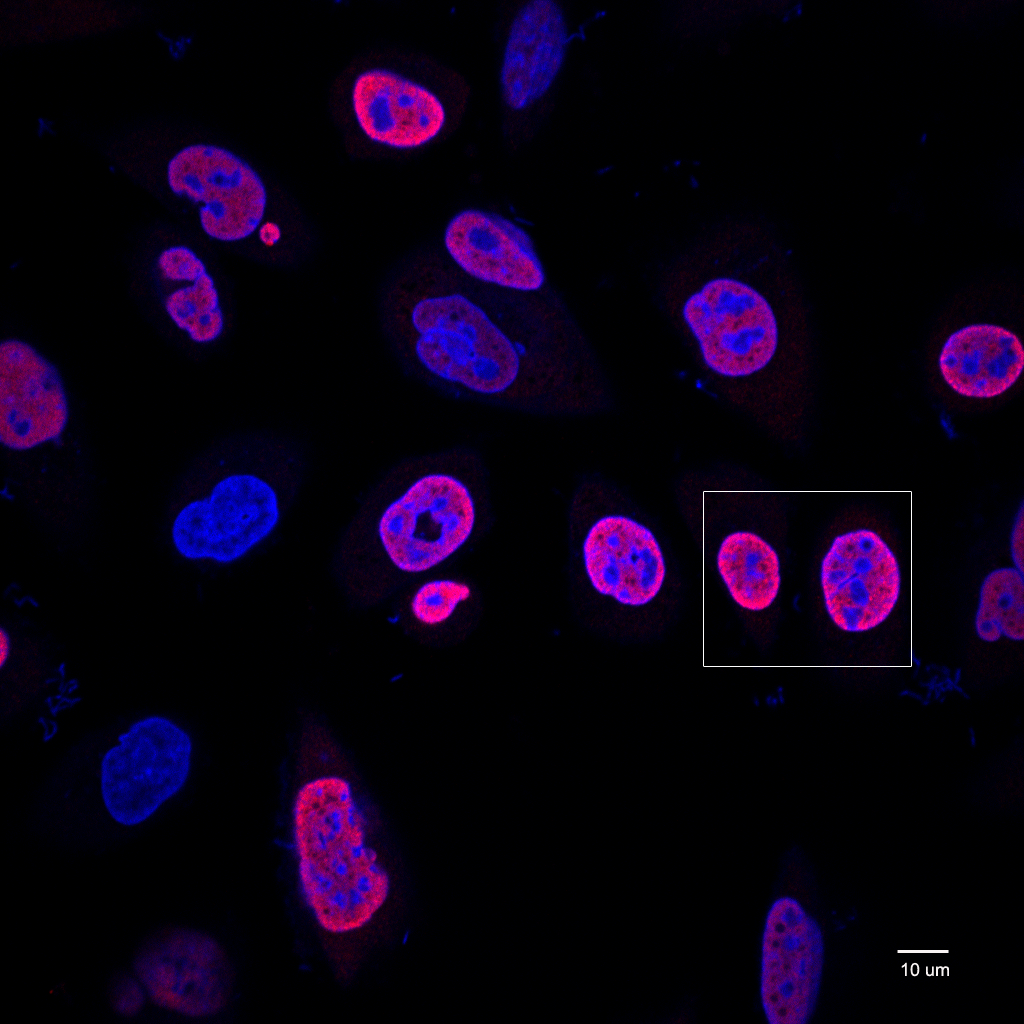

Supplement: Supplementary file 9 — Figure EV1-5 Source Data [file 44318_2024_323_MOESM9_ESM.zip › SD figure EV1-5/SD figure EV5/EV 5A/image R465G Merge.tif]

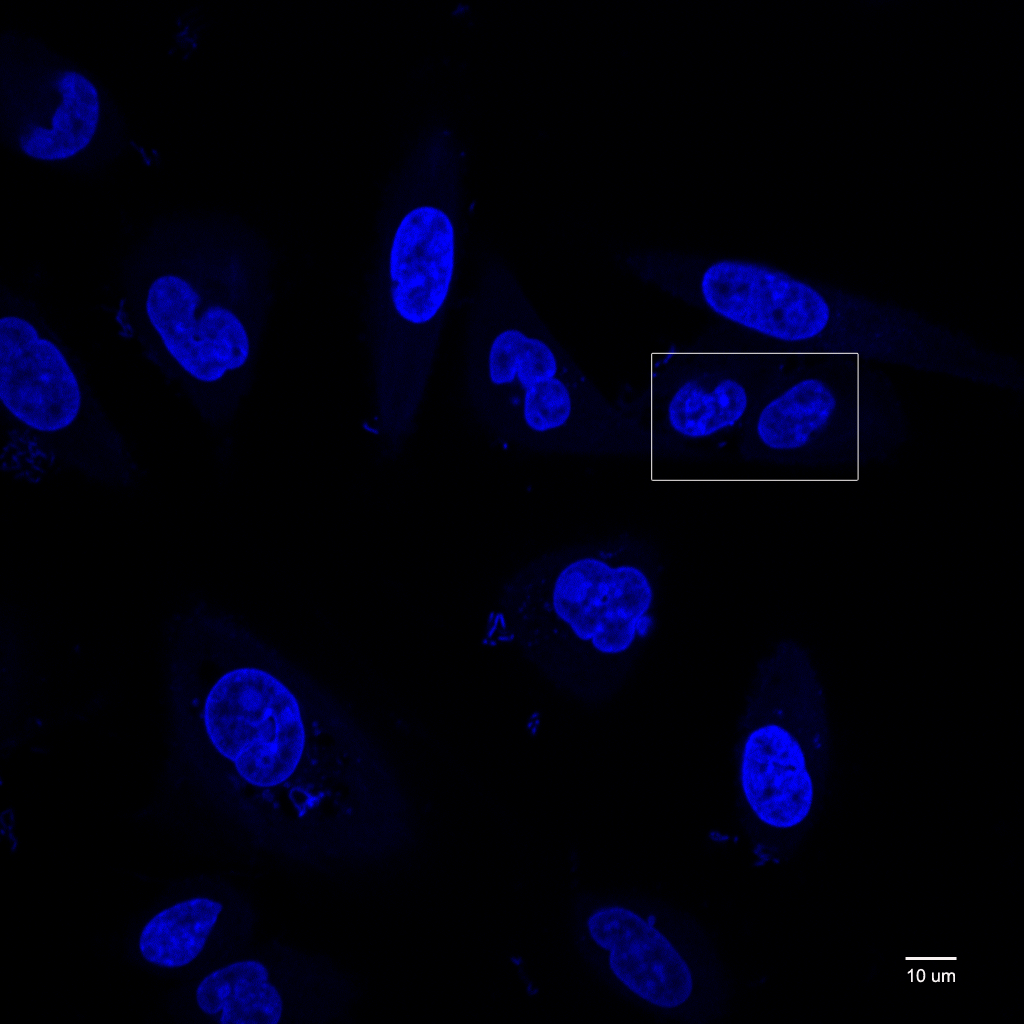

Supplement: Supplementary file 9 — Figure EV1-5 Source Data [file 44318_2024_323_MOESM9_ESM.zip › SD figure EV1-5/SD figure EV5/EV 5A/image R465T DAPI.tif]

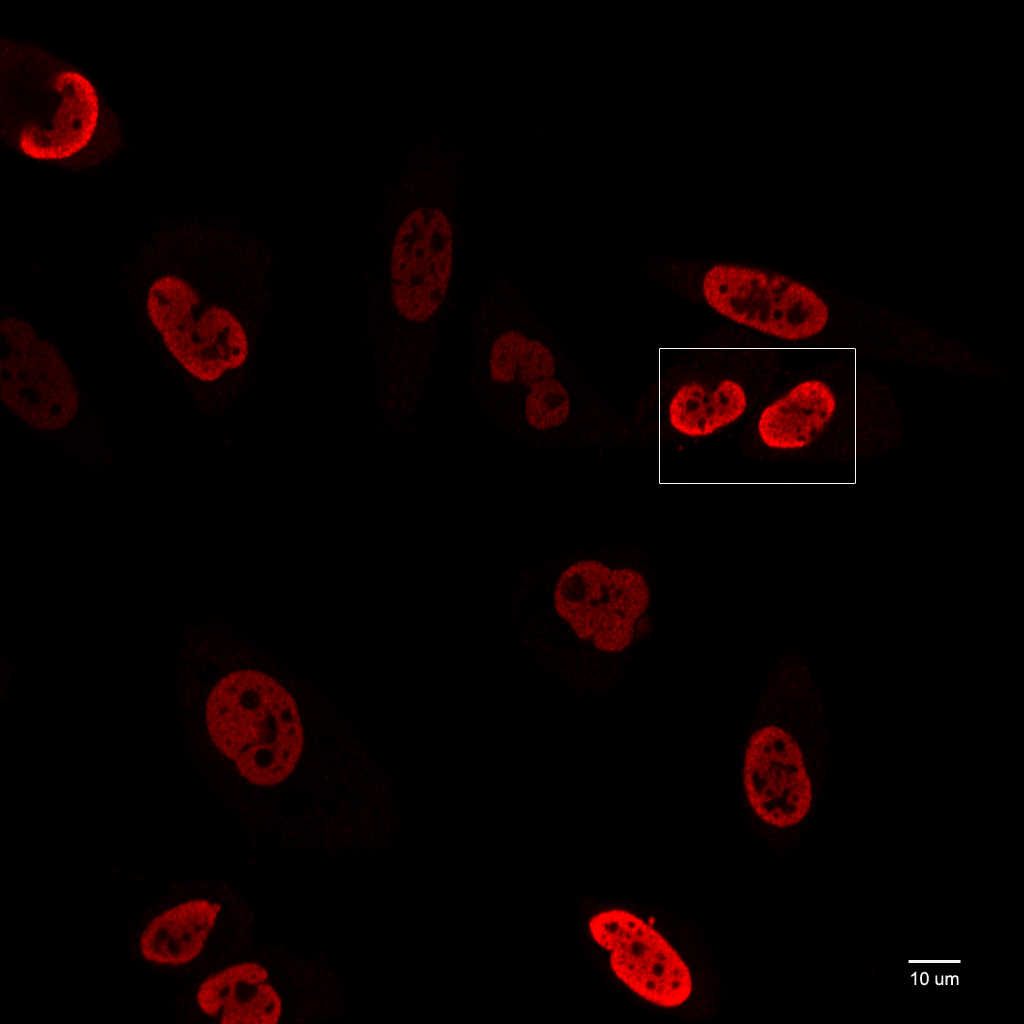

Supplement: Supplementary file 9 — Figure EV1-5 Source Data [file 44318_2024_323_MOESM9_ESM.zip › SD figure EV1-5/SD figure EV5/EV 5A/image R465T FLAG.tif]

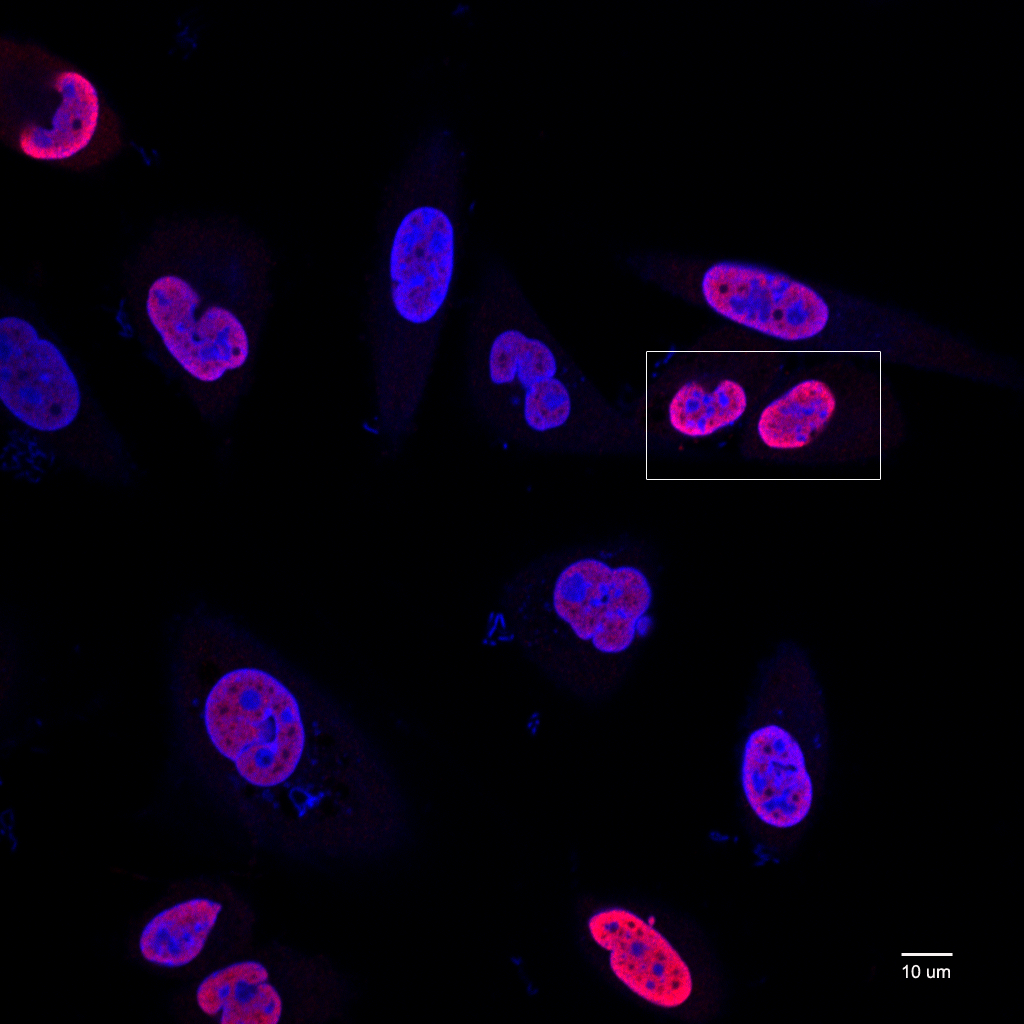

Supplement: Supplementary file 9 — Figure EV1-5 Source Data [file 44318_2024_323_MOESM9_ESM.zip › SD figure EV1-5/SD figure EV5/EV 5A/image R465T Merge.tif]

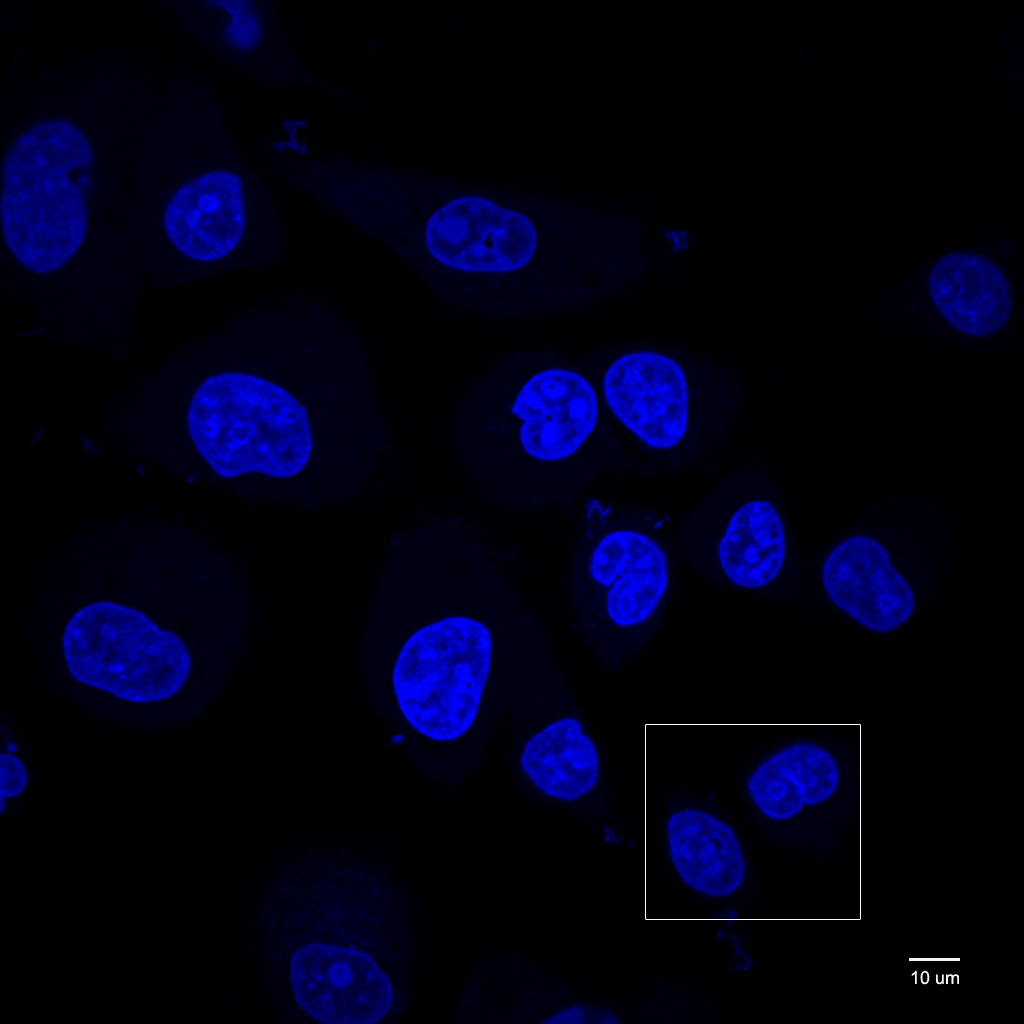

Supplement: Supplementary file 9 — Figure EV1-5 Source Data [file 44318_2024_323_MOESM9_ESM.zip › SD figure EV1-5/SD figure EV5/EV 5A/image R514C DAPI.tif]

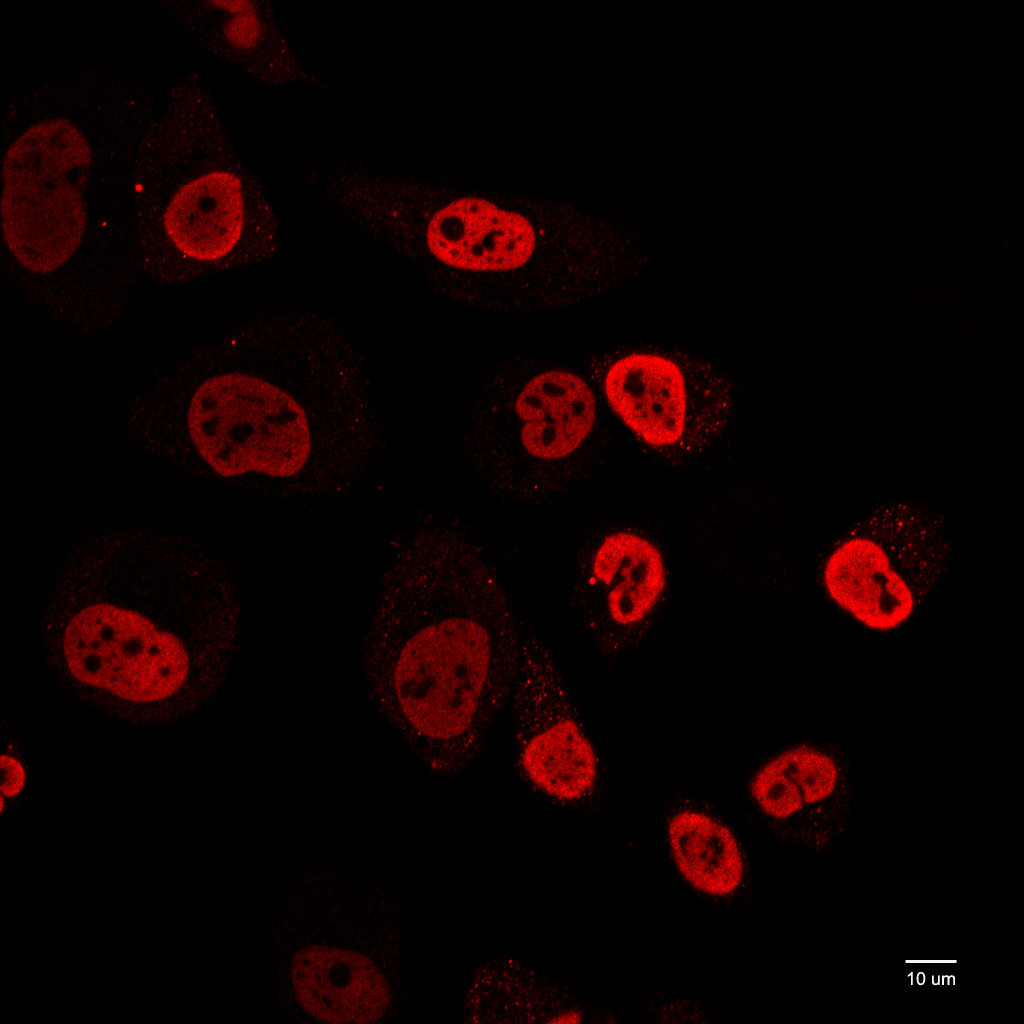

Supplement: Supplementary file 9 — Figure EV1-5 Source Data [file 44318_2024_323_MOESM9_ESM.zip › SD figure EV1-5/SD figure EV5/EV 5A/image R514C FLAG.tif]

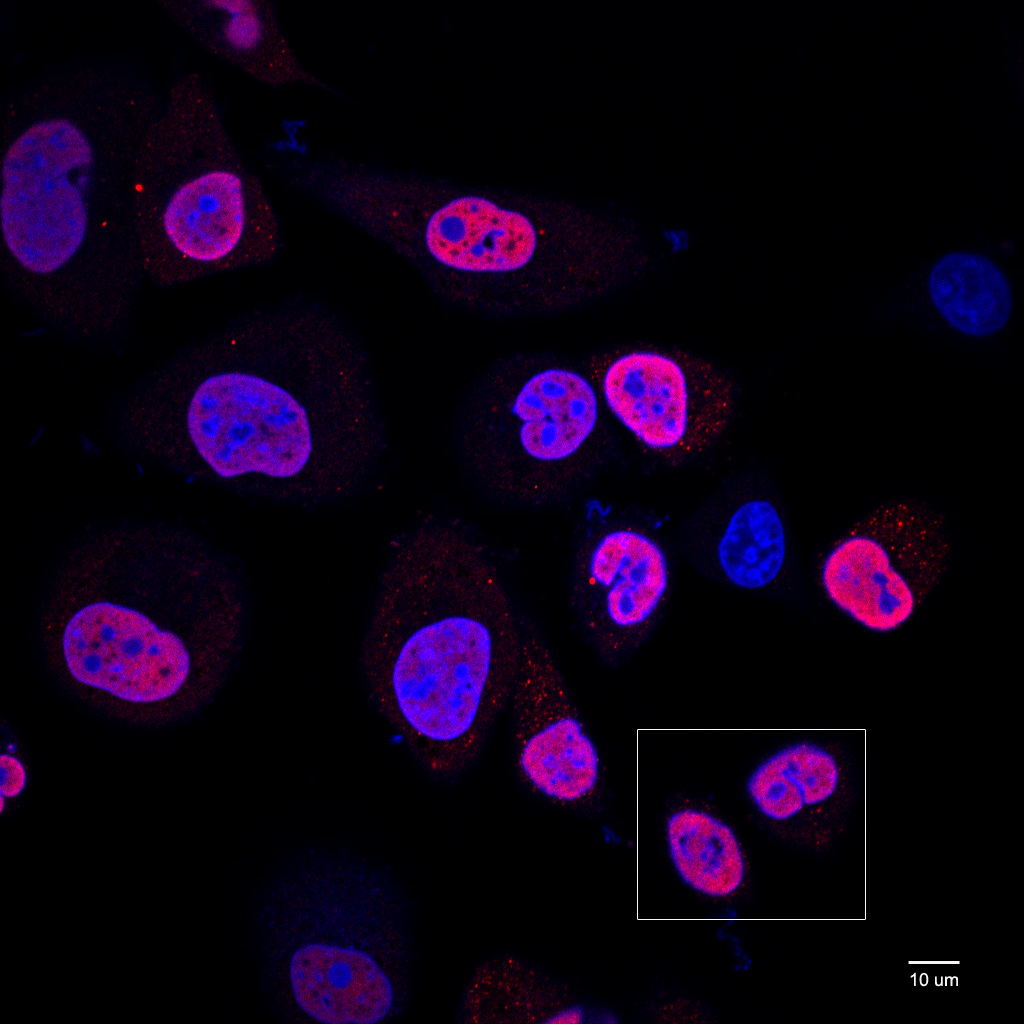

Supplement: Supplementary file 9 — Figure EV1-5 Source Data [file 44318_2024_323_MOESM9_ESM.zip › SD figure EV1-5/SD figure EV5/EV 5A/image R514C Merge.tif]

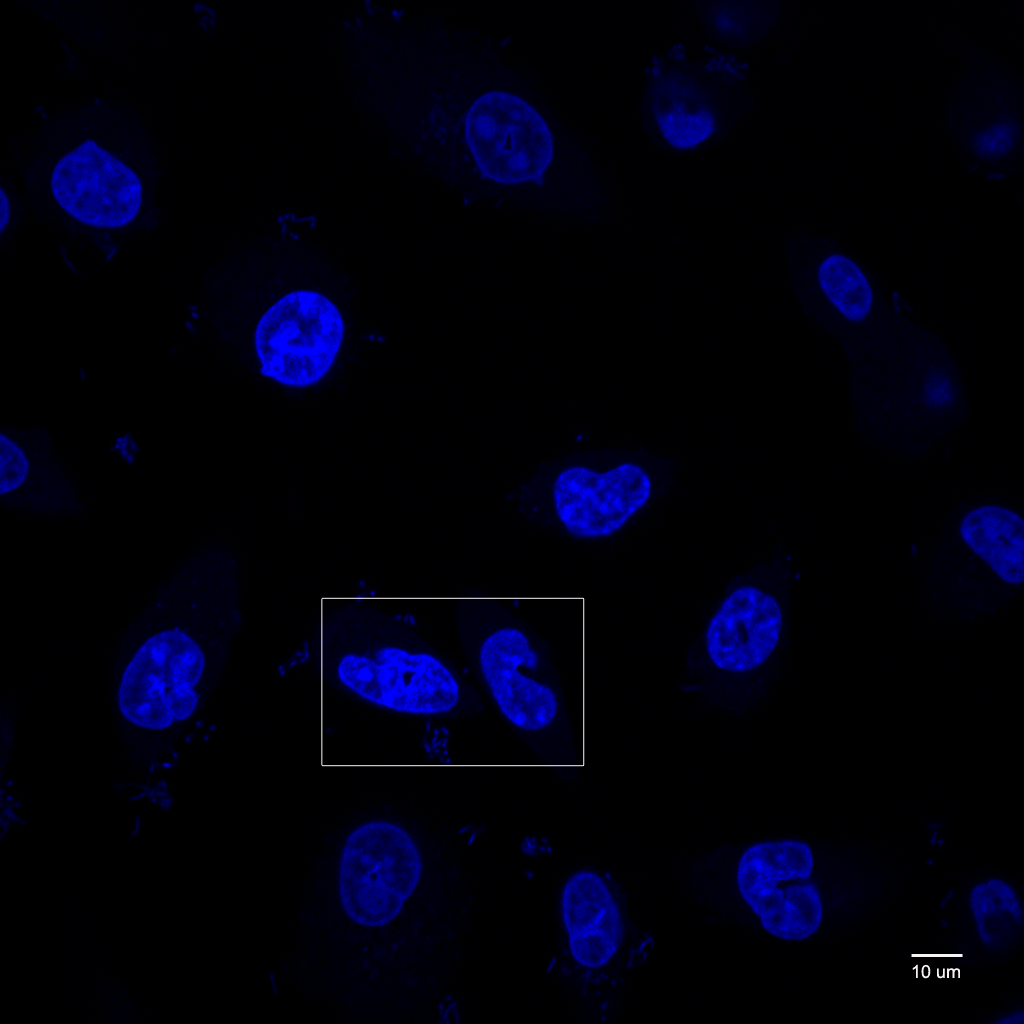

Supplement: Supplementary file 9 — Figure EV1-5 Source Data [file 44318_2024_323_MOESM9_ESM.zip › SD figure EV1-5/SD figure EV5/EV 5A/image R514H DAPI.tif]

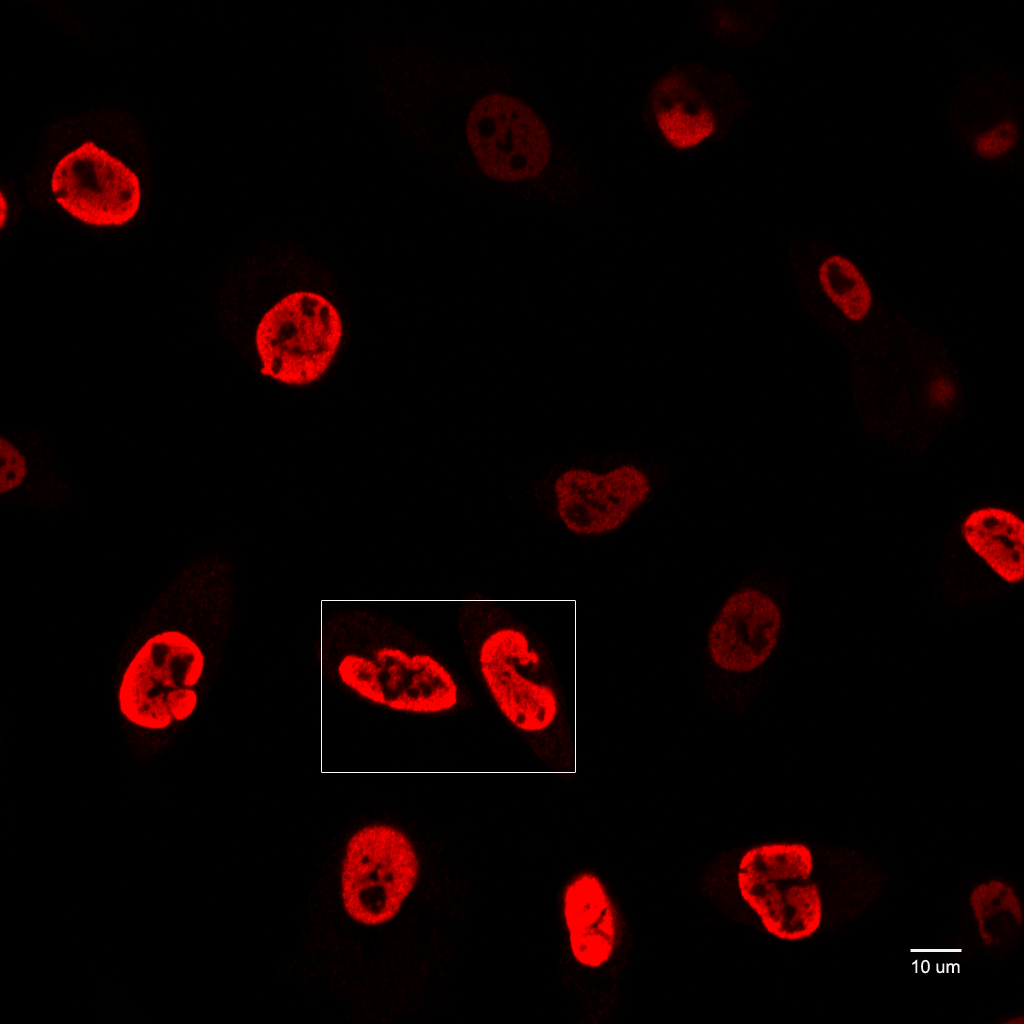

Supplement: Supplementary file 9 — Figure EV1-5 Source Data [file 44318_2024_323_MOESM9_ESM.zip › SD figure EV1-5/SD figure EV5/EV 5A/image R514H FLAG.tif]

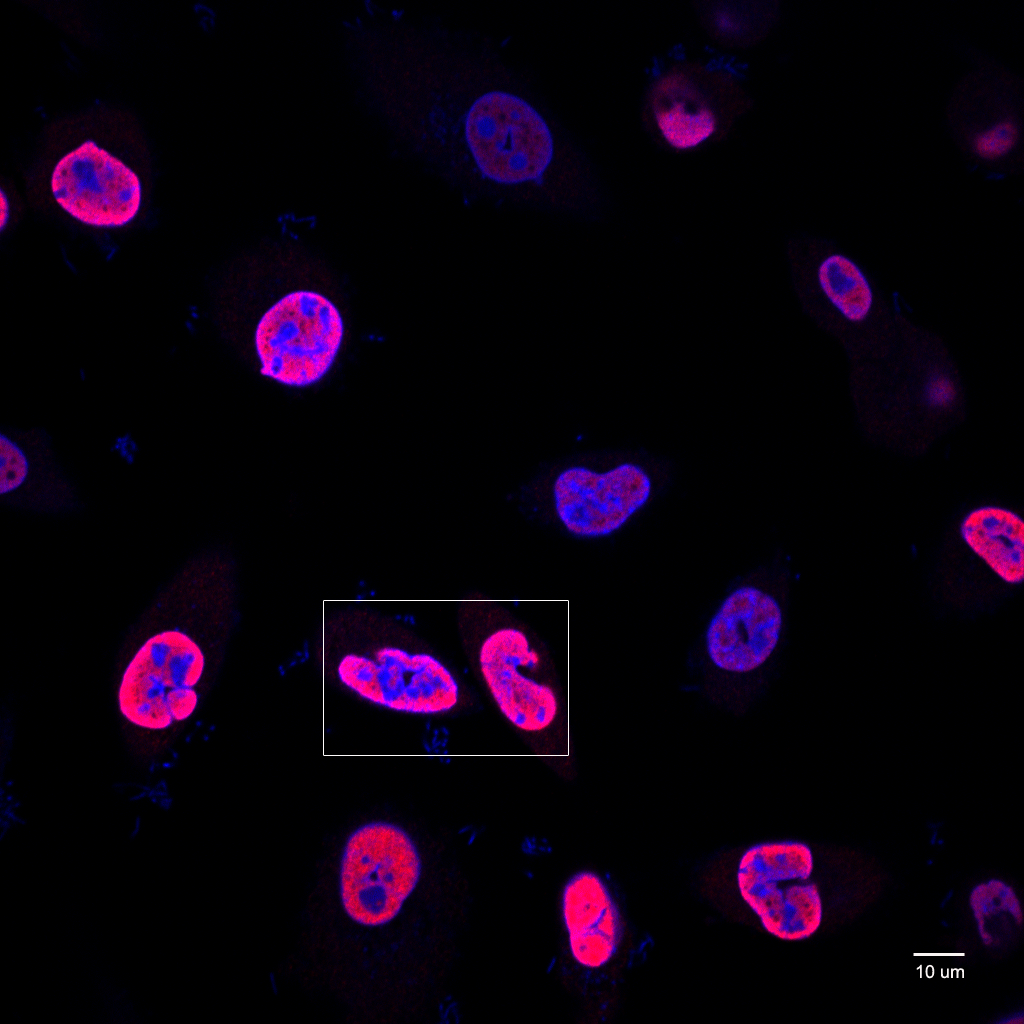

Supplement: Supplementary file 9 — Figure EV1-5 Source Data [file 44318_2024_323_MOESM9_ESM.zip › SD figure EV1-5/SD figure EV5/EV 5A/image R514H Merge.tif]

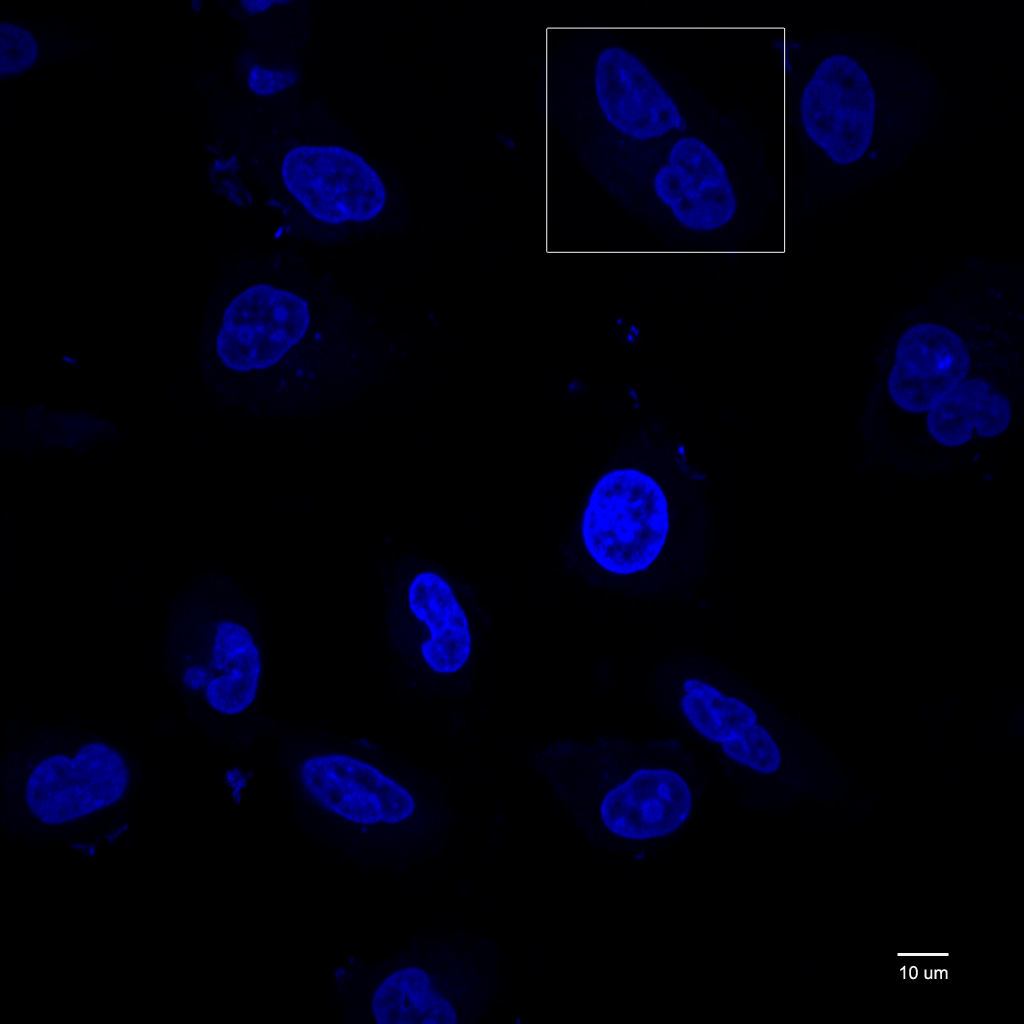

Supplement: Supplementary file 9 — Figure EV1-5 Source Data [file 44318_2024_323_MOESM9_ESM.zip › SD figure EV1-5/SD figure EV5/EV 5A/image WT DAPI.tif]

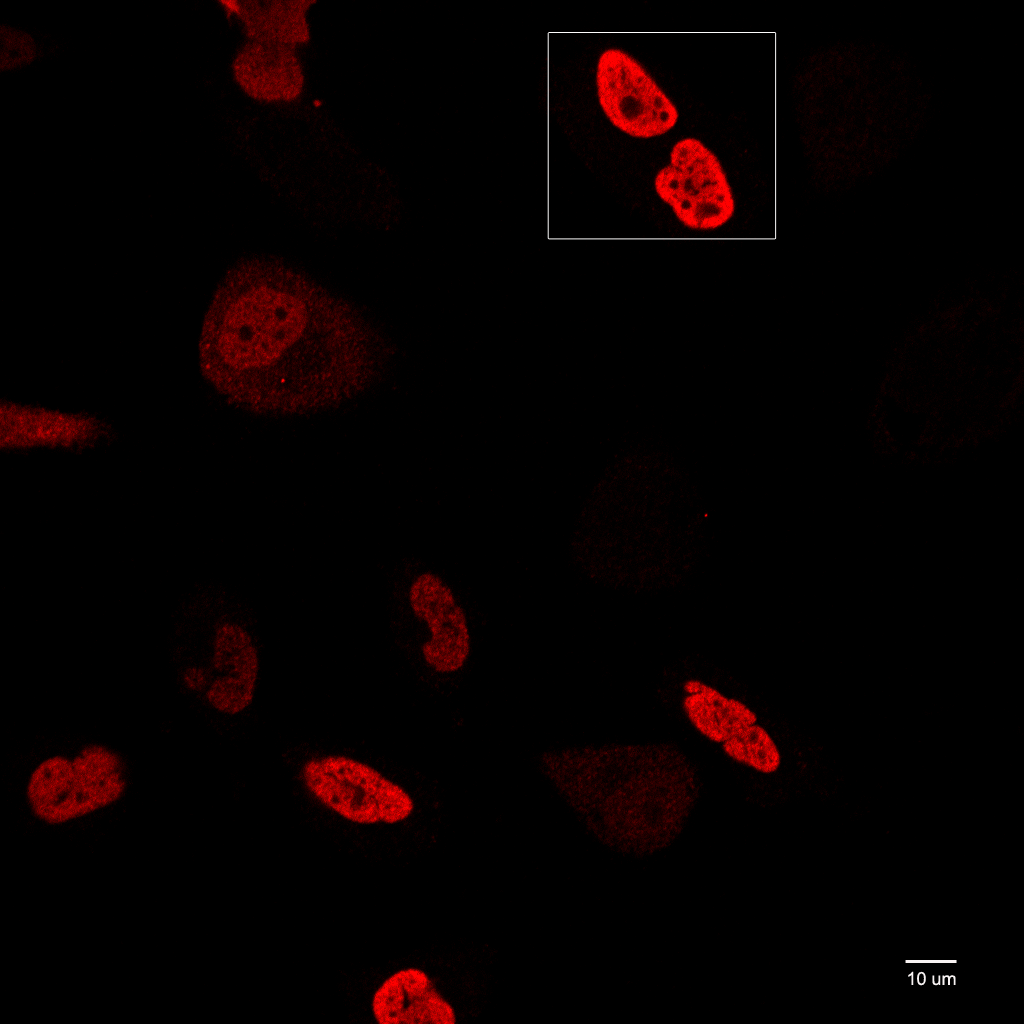

Supplement: Supplementary file 9 — Figure EV1-5 Source Data [file 44318_2024_323_MOESM9_ESM.zip › SD figure EV1-5/SD figure EV5/EV 5A/image WT FLAG.tif]

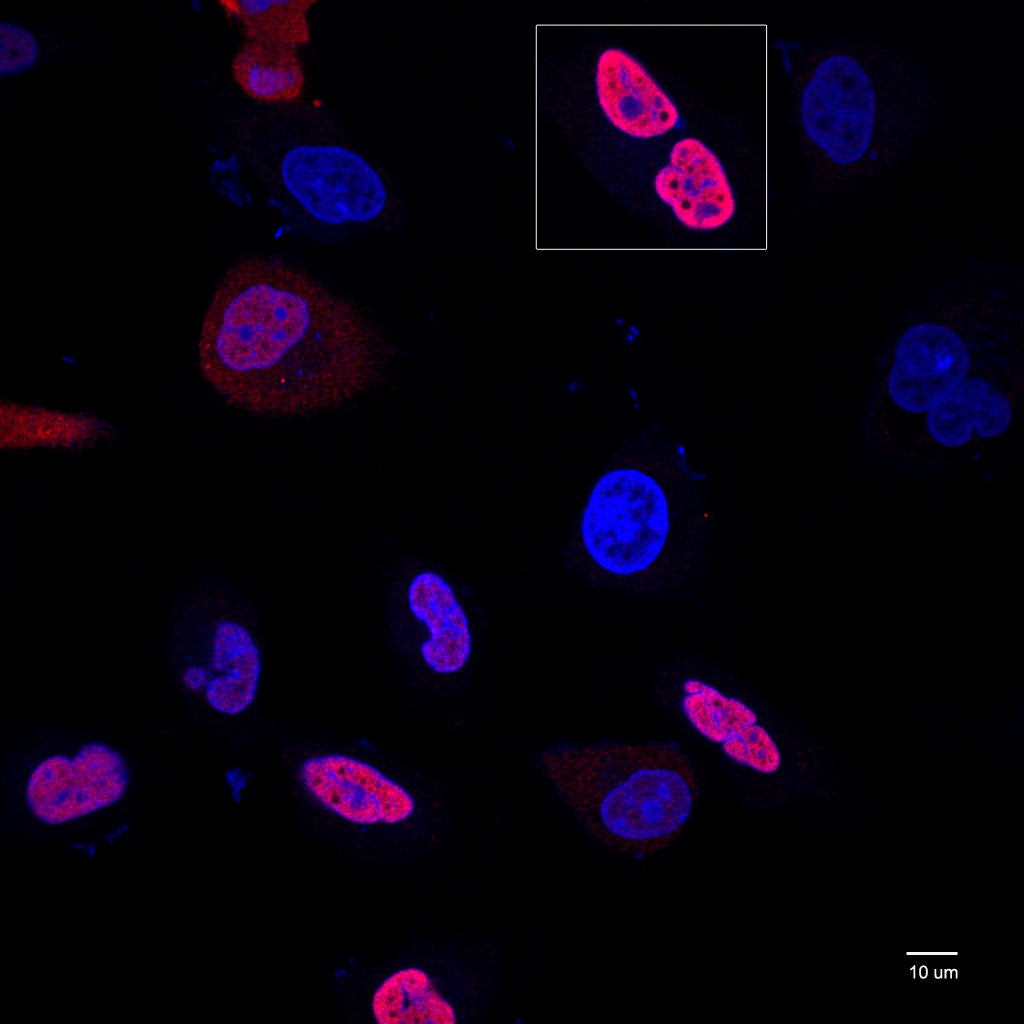

Supplement: Supplementary file 9 — Figure EV1-5 Source Data [file 44318_2024_323_MOESM9_ESM.zip › SD figure EV1-5/SD figure EV5/EV 5A/image WT Merge.tif]

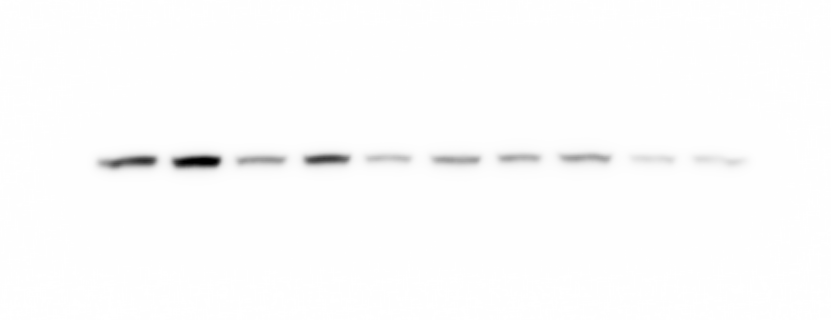

Supplement: Supplementary file 9 — Figure EV1-5 Source Data [file 44318_2024_323_MOESM9_ESM.zip › SD figure EV1-5/SD figure EV5/EV 5B/western FLAG CF.tif]

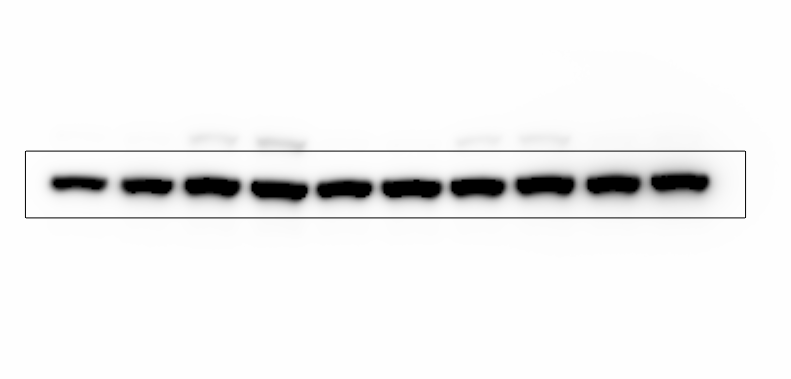

Supplement: Supplementary file 9 — Figure EV1-5 Source Data [file 44318_2024_323_MOESM9_ESM.zip › SD figure EV1-5/SD figure EV5/EV 5B/western FLAG WCL.tif]

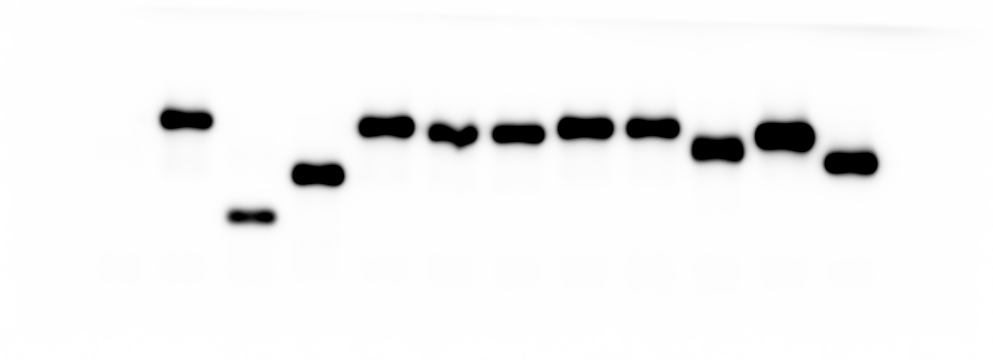

Supplement: Supplementary file 9 — Figure EV1-5 Source Data [file 44318_2024_323_MOESM9_ESM.zip › SD figure EV1-5/SD figure EV5/EV 5C/western FLAG.tif]

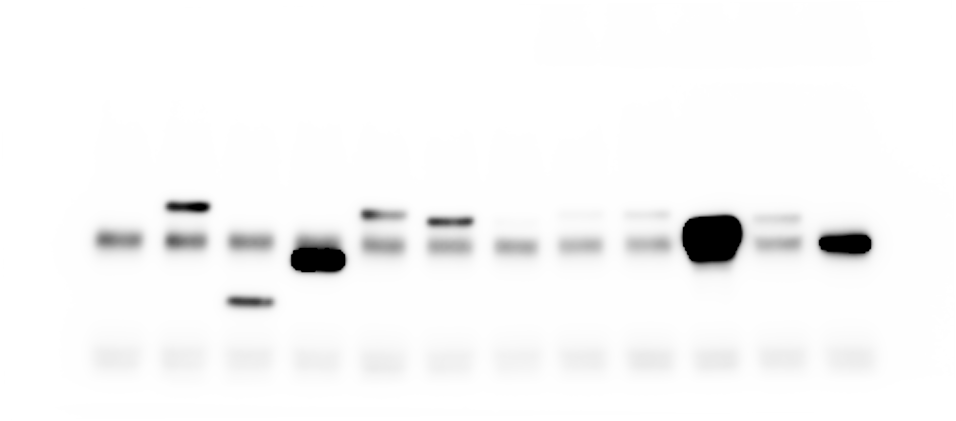

Supplement: Supplementary file 9 — Figure EV1-5 Source Data [file 44318_2024_323_MOESM9_ESM.zip › SD figure EV1-5/SD figure EV5/EV 5C/western O-GlcNAc.tif]

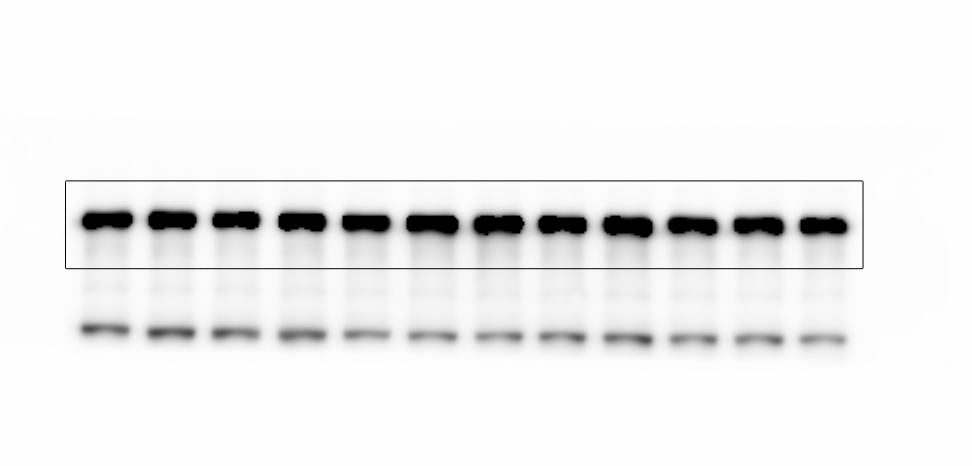

Supplement: Supplementary file 9 — Figure EV1-5 Source Data [file 44318_2024_323_MOESM9_ESM.zip › SD figure EV1-5/SD figure EV5/EV 5D/western ATR INPUT.tif]

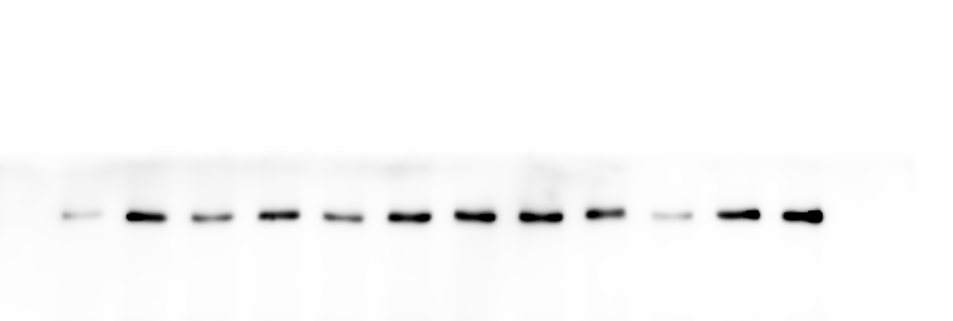

Supplement: Supplementary file 9 — Figure EV1-5 Source Data [file 44318_2024_323_MOESM9_ESM.zip › SD figure EV1-5/SD figure EV5/EV 5D/western ATR IP.tif]

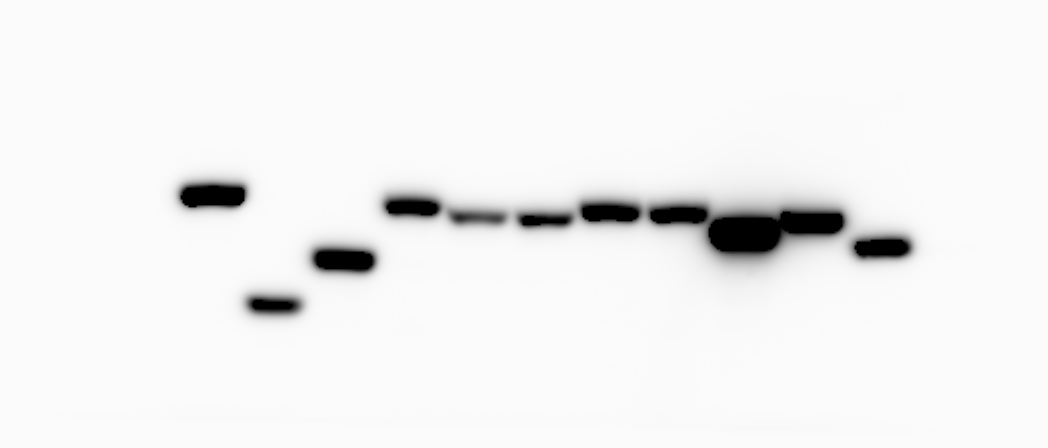

Supplement: Supplementary file 9 — Figure EV1-5 Source Data [file 44318_2024_323_MOESM9_ESM.zip › SD figure EV1-5/SD figure EV5/EV 5D/western FLAG INPUT.tif]

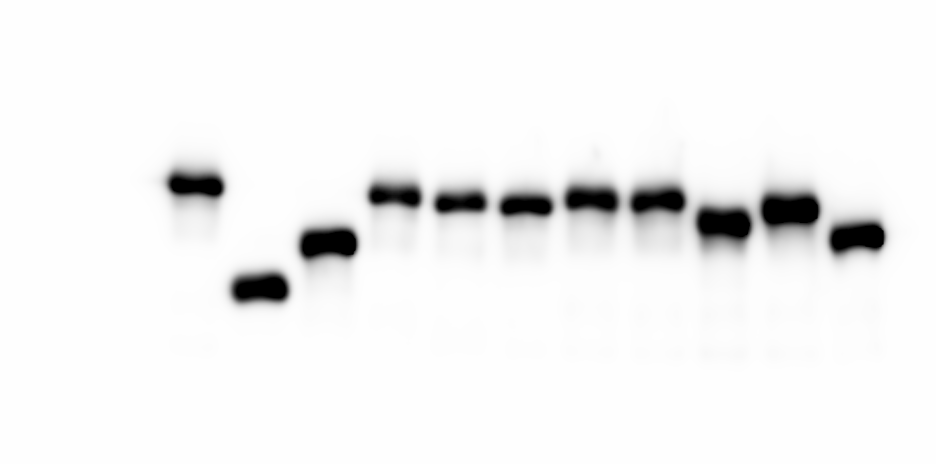

Supplement: Supplementary file 9 — Figure EV1-5 Source Data [file 44318_2024_323_MOESM9_ESM.zip › SD figure EV1-5/SD figure EV5/EV 5D/western FLAG IP.tif]

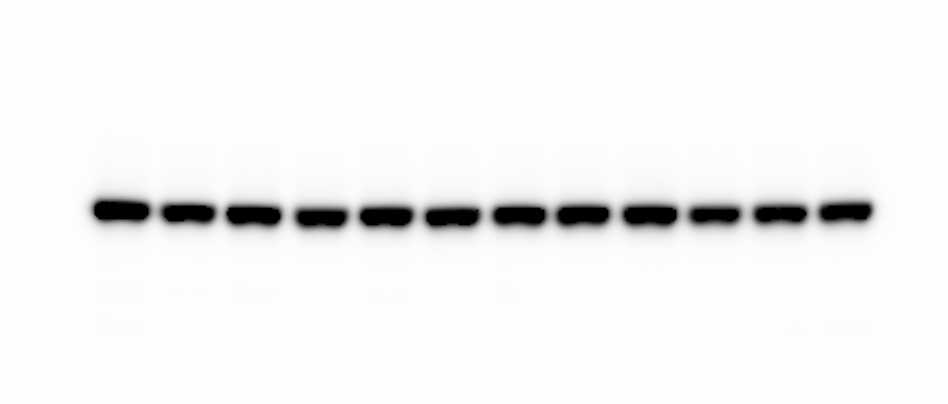

Supplement: Supplementary file 9 — Figure EV1-5 Source Data [file 44318_2024_323_MOESM9_ESM.zip › SD figure EV1-5/SD figure EV5/EV 5D/western HA INPUT.tif]

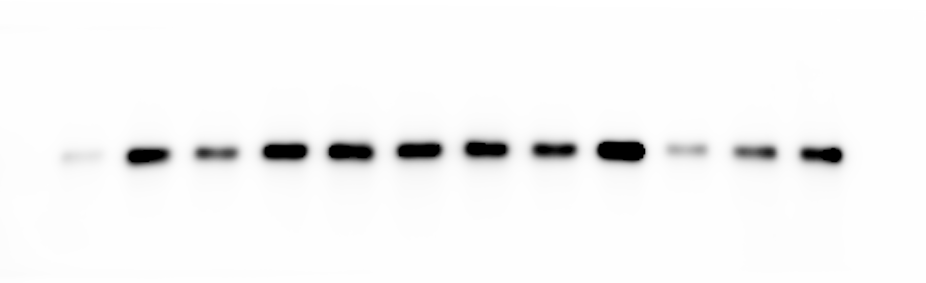

Supplement: Supplementary file 9 — Figure EV1-5 Source Data [file 44318_2024_323_MOESM9_ESM.zip › SD figure EV1-5/SD figure EV5/EV 5D/western HA IP.tif]

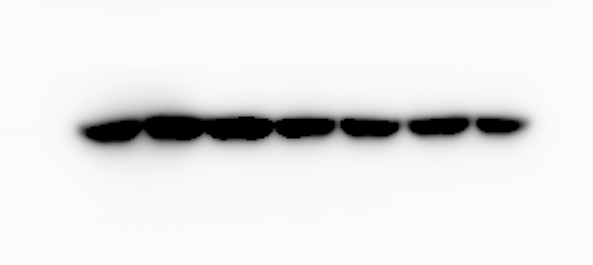

Supplement: Supplementary file 9 — Figure EV1-5 Source Data [file 44318_2024_323_MOESM9_ESM.zip › SD figure EV1-5/SD figure EV5/EV 5E/western Actin.tif]

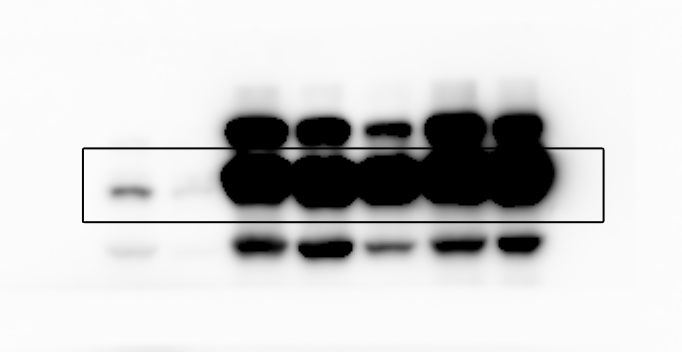

Supplement: Supplementary file 9 — Figure EV1-5 Source Data [file 44318_2024_323_MOESM9_ESM.zip › SD figure EV1-5/SD figure EV5/EV 5E/western FOXP1.tif]
