# Supplementary material for: FOXP1 phosphorylation antagonizes its O-GlcNAcylation in regulating ATR activation in response to replication stress
Source: EMBO J. 2024 Dec 2;44(2):457–83. doi: 10.1038/s44318-024-00323-x (PMC11729909; doi:10.1038/s44318-024-00323-x)
Supplement: Supplementary file 10 — Expanded View Figures [file 44318_2024_323_MOESM10_ESM.pdf]

Expanded View Figures

Figure EV1. FOXP1 promotes ATR activation.

(**A**) Coomassie blue staining of immunoprecipitates enriched with ATR-specific antibody. (**B**) His-tagged FOXP1 was incubated with GST-tagged ATRIP followed by GST pull-down assay. Proteins bound onto Glutathione beads were detected via immunoblotting using the indicated antibodies. (**C**) H1975 cells transfected with negative control siRNA or different siRNA targeting FOXP1 were treated with HU for 1 h before the whole cell lysates were harvested for immunoblotting with the indicated antibodies. (**D**) Statistical analysis of the IdU/CldU ratio mean in Fig. 1E, mean (biological replicates, $n = 3$) ± SD is shown. ***$P < 0.001$; ns, no significance; P values were calculated by one-way ANOVA, followed by Dunnett's test. P value: siNC vs siFOXP1-1, 0.0004; siNC vs siFOXP1-2, 0.0001; siFOXP1-1 vs siFOXP1-2, 0.9510. (**E**) HEK293T or H1975 cells transfected with siNC or siFOXP1 were pulse labeled with 10 μM BrdU for 30 min, followed by BrdU and PI staining and flow cytometric analysis. The percentage represents the BrdU-positive cells. (**F**) HEK293 cells transfected with negative control siRNA or different siRNA targeting FOXP1 were incubated with 100 nM CPT for 8 h or left untreated before harvested for PI staining and flow cytometric analysis. The percentage of S phase population was analyzed, mean (biological replicates, $n = 3$) ± SD is shown. ****$P < 0.0001$, P values were calculated by two-way ANOVA, followed by Sidak's test. P value: siNC vs siFOXP1-1, 6.40e-006; siNC vs siFOXP1-2, 1.02e-006. (**G**) The whole cell lysates of HEK293T or H1975 cells transfected with siNC or siFOXP1 were harvested for immunoblotting with the indicated antibodies. (**H**) H1975 cells with different FOXP1 levels were subjected to sequential labeling with CldU and IdU for 30 min each, followed by DNA fiber assay. Left: representative images of CldU and IdU replication tracks. Middle: the IdU tract length was analyzed, IdU length mean (red line) ±SD is shown in scatter plot, n, DNA fiber number; ns, no significance; P values were calculated by one-way ANOVA, followed by Kruskal–Wallis test. P value: siNC vs siFOXP1-1, $P > 0.9999$; siNC vs siFOXP1-2, $P > 0.9999$; siFOXP1-1 vs siFOXP1-2, $P > 0.9999$. Right: Mean IdU length ± SD (biological replicates, $n = 3$) was also analyzed and shown in column, ns, no significance, P values were calculated by one-way ANOVA, followed by Dunnett's test. P value: siNC vs siFOXP1-1, 0.9898; siNC vs siFOXP1-2, 0.7224; siFOXP1-1 vs siFOXP1-2, 0.6490. (**I**) HEK293T cells transfected with FLAG-FOXP1 treated with 2 mM HU for 1 h were subjected to chromatin fractionation. Both the soluble and chromatin fractions were subjected to immunoprecipitation using a FLAG-specific antibody. Source data are available online for this figure.

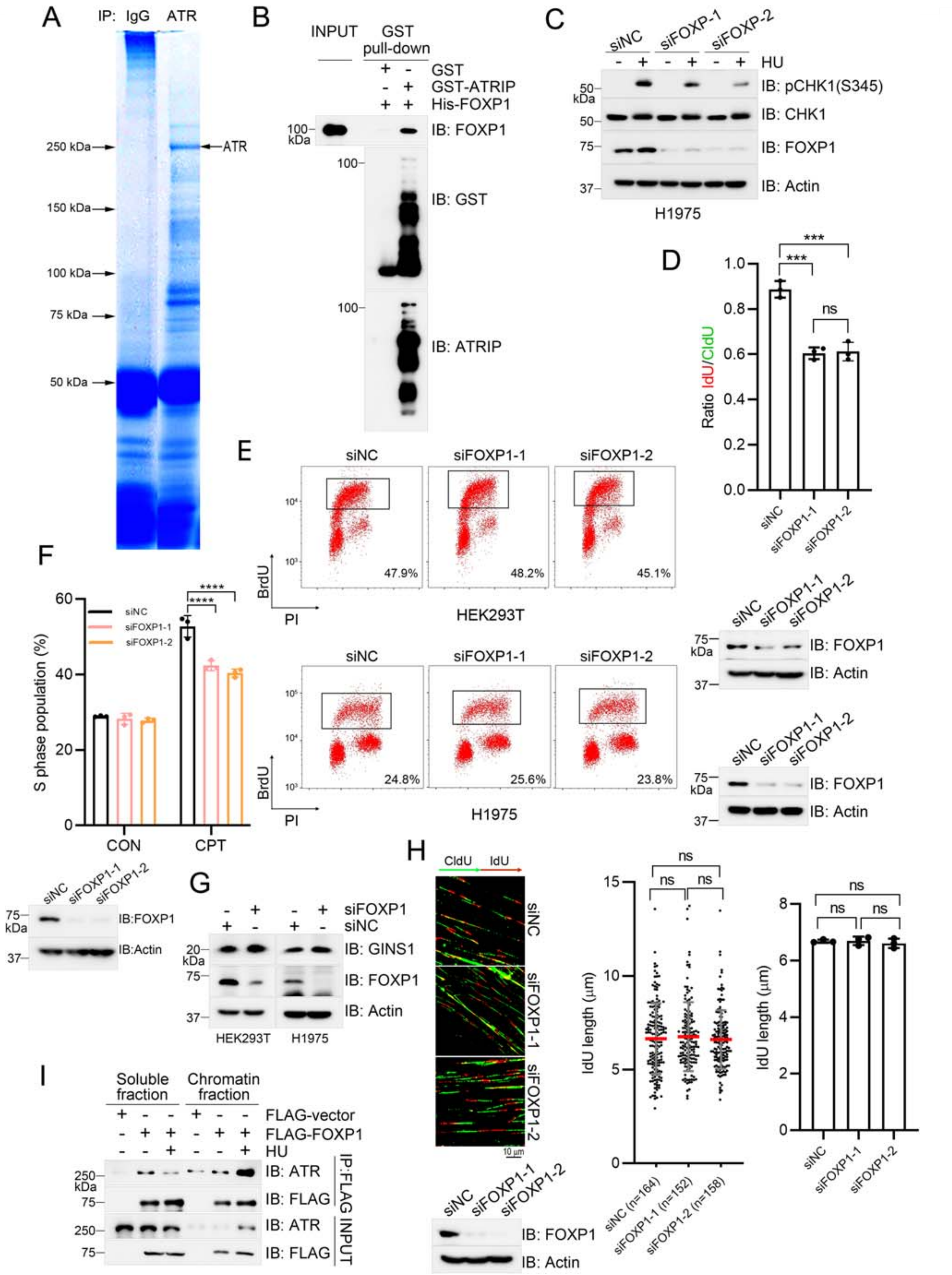
A
IP: IgG ATR
250 kDa
ATR
150 kDa
100 kDa
75 kDa
50 kDa
B
INPUT
GST pull-down
GST
GST-ATRIP
His-FOXP1
IB: FOXP1
IB: GST
IB: ATRIP
C
siNC
siFOXP-1
siFOXP-2
HU
IB: pCHK1(S345)
IB: CHK1
IB: FOXP1
IB: Actin
H1975
D
Ratio IdU/CldU
siNC
siFOXP1-1
siFOXP1-2
E
HEK293T
H1975
BrdU
PI
47.9%
48.2%
45.1%
24.8%
25.6%
23.8%
F
S phase population (%)
CON
CPT
G
siFOXP1
siNC
IB: GINS1
IB: FOXP1
IB: Actin
HEK293T
H1975
H
CldU
IdU
IdU length (μm)
siNC (n=164)
siFOXP1-1 (n=152)
siFOXP1-2 (n=158)
I
Soluble fraction
Chromatin fraction
FLAG-vector
FLAG-FOXP1
HU
IB: ATR
IB: FLAG
IP:FLAG
INPUT

 © The Author(s)

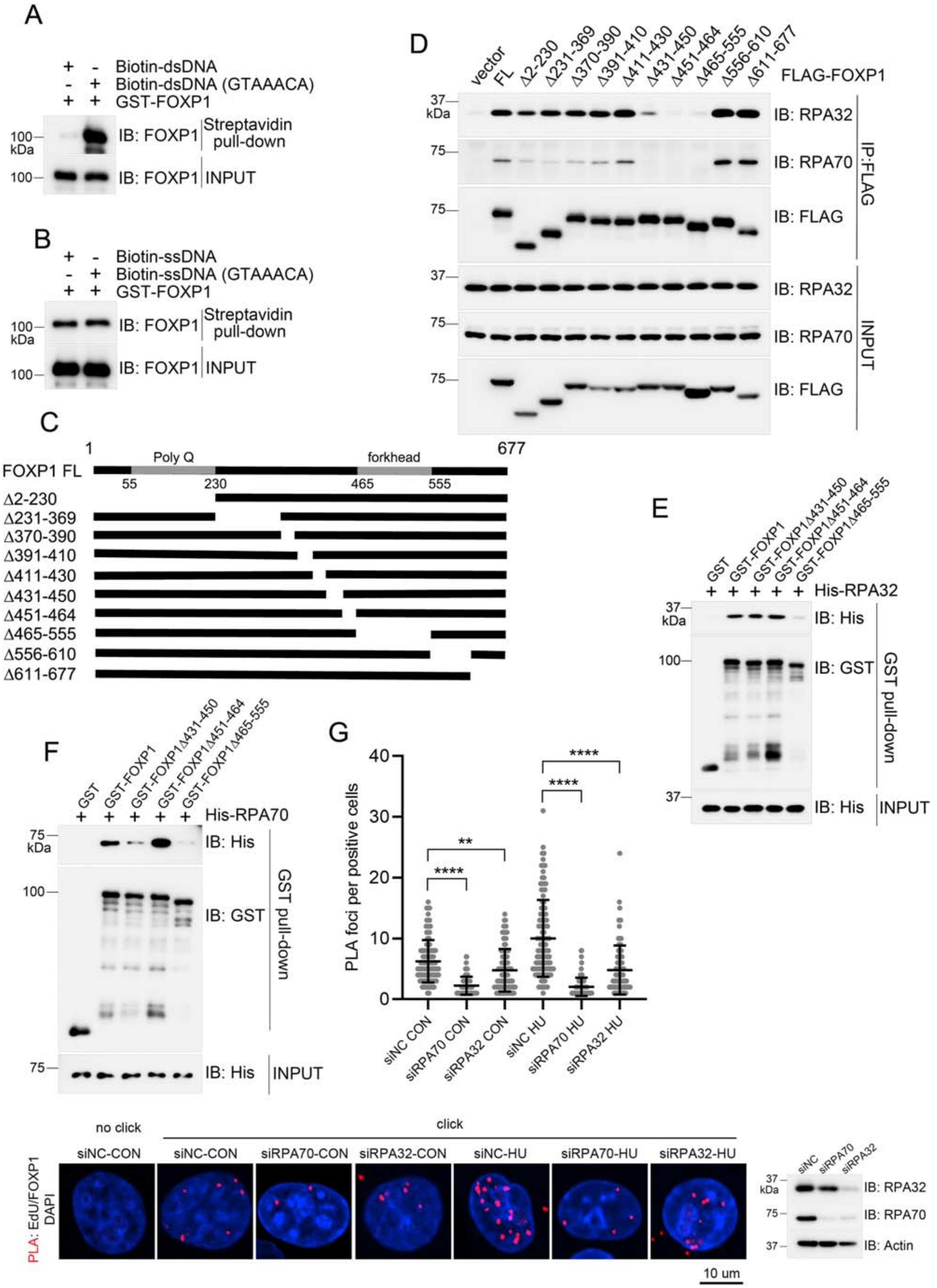
A
Biotin-dsDNA
Biotin-dsDNA (GTAAACA)
GST-FOXP1
IB: FOXP1
Streptavidin pull-down
IB: FOXP1
INPUT
B
Biotin-ssDNA
Biotin-ssDNA (GTAAACA)
GST-FOXP1
Streptavidin pull-down
INPUT
C
FOXP1 FL
Poly Q
forkhead
Δ2-230
Δ231-369
Δ370-390
Δ391-410
Δ411-430
Δ431-450
Δ451-464
Δ465-555
Δ556-610
Δ611-677
D
vector FL Δ2-230 Δ231-369 Δ370-390 Δ391-410 Δ411-430 Δ431-450 Δ451-464 Δ465-555 Δ556-610 Δ611-677
FLAG-FOXP1
IB: RPA32
IB: RPA70
IB: FLAG
IP:FLAG
INPUT
E
GST GST-FOXP1 GST-FOXP1Δ431-450 GST-FOXP1Δ451-464 GST-FOXP1Δ465-555
His-RPA32
IB: His
IB: GST
GST pull-down
INPUT
F
His-RPA70
G
PLA foci per positive cells
siNC CON
siRPA70 CON
siRPA32 CON
siNC HU
siRPA70 HU
siRPA32 HU
no click
click
siNC-CON
siRPA70-CON
siRPA32-CON
siNC-HU
siRPA70-HU
siRPA32-HU
PLA: EdU/FOXP1
DAPI
10 um
siNC siRPA70 siRPA32
IB: Actin

Figure EV2. Interaction mapping between FOXP1 and RPA.

(**A**) Biotin-labeled random dsDNA or dsDNA containing GTAAACA consensus motif was conjugated on streptavidin magnetic beads and incubated with GST-tagged FOXP1 purified from *E. coli*. Streptavidin-bound FOXP1 was detected via immunoblotting with the indicated antibodies. (**B**) Biotin-labeled random ssDNA or ssDNA containing GTAAACA was used to test FOXP1 binding as described in (**A**). (**C**) Schematic of full-length FOXP1 and its deletion mutants. (**D**) HEK293T cells transfected with FLAG-FOXP1 or its deletion mutants were subjected to immunoprecipitation using a FLAG-specific antibody. FOXP1 bound RPA proteins were detected via immunoblotting. (**E**, **F**) His-tagged RPA32 (**E**) or RPA70 (**F**) were incubated with GST-tagged FOXP1 (or its deletion mutants) followed by GST pull-down assay. Proteins bound onto Glutathione beads were detected via immunoblotting using the indicated antibodies. (**G**) Proximity ligation assay experiments using FOXP1 and biotin-specific antibodies in H1975 cells transfected with a negative control siRNA or siRNAs targeting RPA32 or RPA70. Upper panel: quantification of the number of PLA foci per foci-positive cells (cell number: siNC CON, $n = 103$; siRPA70-CON, $n = 102$; siRPA32-CON, $n = 102$; siNC HU, $n = 110$; siRPA70-CON, $n = 106$; siRPA32-CON, $n = 106$), mean ± SD is shown. **$P < 0.01$, ****$P < 0.0001$, P values were calculated by one-way ANOVA, followed by Kruskal–Wallis test. P value: siNC CON vs siRPA70-CON, 1.39e-018; siNC CON vs siRPA32-CON, 0.0039; siNC HU vs siRPA70 HU, 5.80e-038; siNC HU vs siRPA32 HU, 1.41e-010. Lower panel: representative images of PLA foci and immunoblotting of RPA expression. Source data are available online for this figure.

 © The Author(s)

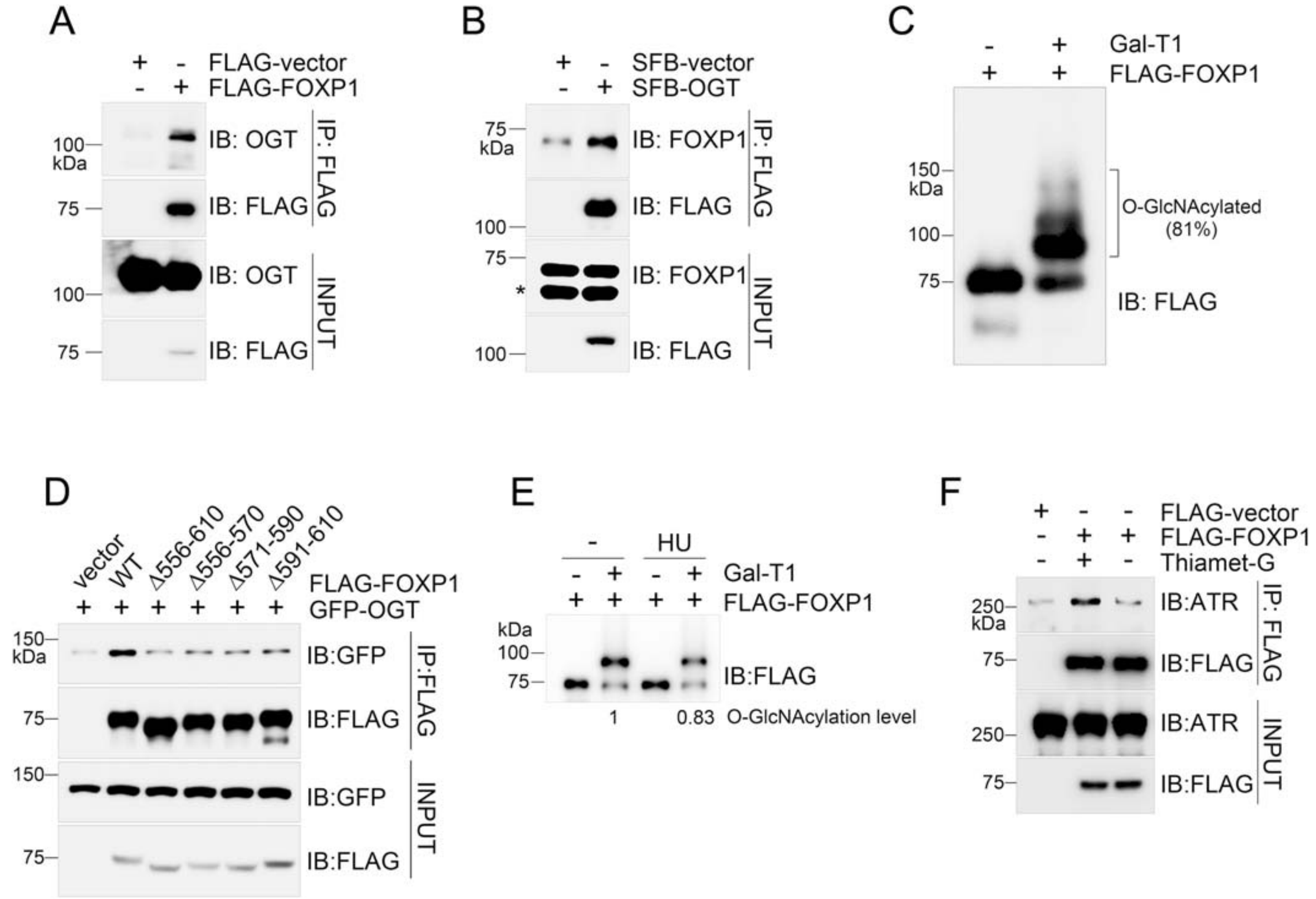

Figure EV3. O-GlcNAcylation of FOXP1 represses its interaction with ATR.

(**A**, **B**) HEK293T cells transfected with FLAG-tagged FOXP1 (**A**) or SFB-tagged OGT (**B**) were subjected to immunoprecipitation using a FLAG-specific antibody. Proteins in the immunoprecipitates were detected via immunoblotting. * a shorter isoform of FOXP1. (**C**) O-GlcNAcylation stoichiometry of FLAG-tagged FOXP1 was analyzed and examined via immunoblotting with FLAG-specific antibody. (**D**) HEK293T cells transfected with GFP-tagged OGT and full-length FLAG-FOXP1 or its deletion mutants were subjected to immunoprecipitation and immunoblotting using the indicated antibodies. (**E**) O-GlcNAcylation stoichiometry of FLAG-tagged FOXP1 was analyzed and examined via immunoblotting with FLAG-specific antibody, with or without incubation with 2 mM HU for 1 h before cells were harvested. (**F**) H1975 cells transfected with FLAG-tagged FOXP1 preincubated with Thiamet-G or not were subjected to immunoprecipitation and immunoblotting using the indicated antibodies. Source data are available online for this figure.

© The Author(s)

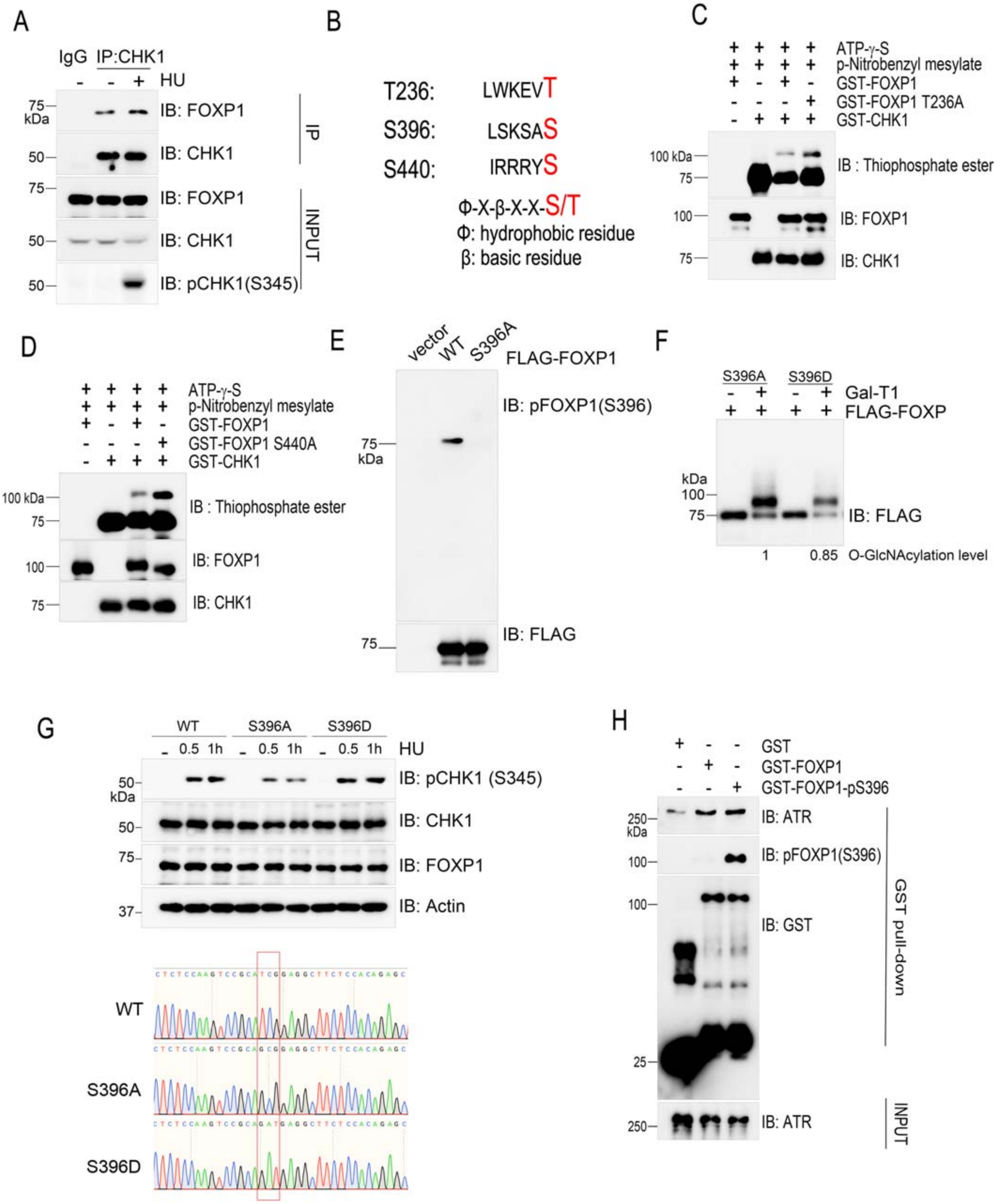
A
IgG
IP:CHK1
HU
IB: FOXP1
IB: CHK1
IB: FOXP1
IB: CHK1
IB: pCHK1(S345)
IP
INPUT
B
T236: LWKEVT
S396: LSKSAS
S440: IRRRYS
Φ-X-β-X-X-S/T
Φ: hydrophobic residue
β: basic residue
C
ATP-γ-S
p-Nitrobenzyl mesylate
GST-FOXP1
GST-FOXP1 T236A
GST-CHK1
IB : Thiophosphate ester
IB: FOXP1
IB: CHK1
D
ATP-γ-S
p-Nitrobenzyl mesylate
GST-FOXP1
GST-FOXP1 S440A
GST-CHK1
IB : Thiophosphate ester
IB: FOXP1
IB: CHK1
E
vector
WT
S396A
FLAG-FOXP1
IB: pFOXP1(S396)
IB: FLAG
F
S396A
S396D
Gal-T1
FLAG-FOXP
IB: FLAG
1
0.85
O-GlcNAcylation level
G
WT
S396A
S396D
HU
IB: pCHK1 (S345)
IB: CHK1
IB: FOXP1
IB: Actin
WT
S396A
S396D
H
GST
GST-FOXP1
GST-FOXP1-pS396
IB: ATR
IB: pFOXP1(S396)
IB: GST
GST pull-down
IB: ATR
INPUT

Figure EV4. CHK1-mediated FOXP1 phosphorylation at S396 promotes ATR activation.

(**A**) HEK293T cells treated with 2 mM HU for 1 h or untreated were subjected to immunoprecipitation and immunoblotting using the indicated antibodies. (**B**) The potential phosphorylation motif recognized by CHK1 on FOXP1. (**C**, **D**) In vitro phosphorylation of FOXP1 and its T236A (**C**) or S440A mutant (**D**) by CHK1 were conducted using ATR-γ-S as the phosphor group donor. (**E**) HEK293T cells transfected with FLAG-tagged FOXP1 or its S396A mutant were subjected to immunoprecipitation using a FLAG-specific antibody. The immunoprecipitates were examined via immunoblotting using a specific antibody to detect FOXP1 phosphorylation at S396. (**F**) O-GlcNAcylation stoichiometry of FLAG-tagged FOXP1 S396A or S396D were analyzed and examined via immunoblotting with FLAG-specific antibody. (**G**) Upper panel: Wild-type and S396A or S396D knock-in HEK293 cells were treated with HU for the indicated time before the whole cell lysates were harvested for immunoblotting with the indicated antibodies. Lower panel: genome sequence of wild-type and mutant HEK293 cell lines. (**H**) GST-tagged FOXP1 were subjected to in vitro kinase assay catalyzed by CHK1 or not, followed by incubation with HEK293T cell lysate before GST pulldown. The glutathione bead-bound signals were detected by immunoblotting. Source data are available online for this figure.

© The Author(s)

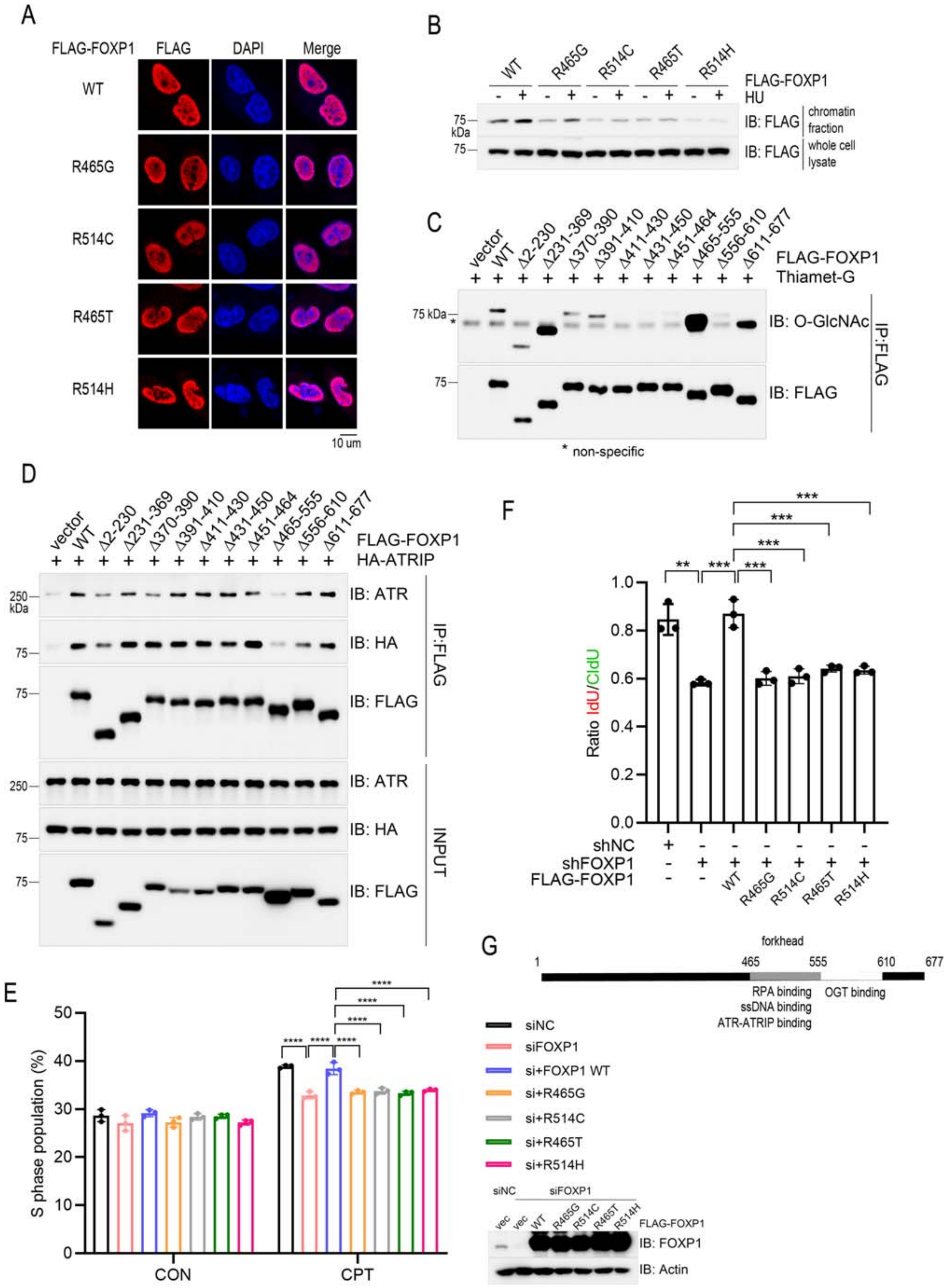
A
FLAG-FOXP1
FLAG
DAPI
Merge
WT
R465G
R514C
R465T
R514H
10 um
B
FLAG-FOXP1
HU
IB: FLAG chromatin fraction
IB: FLAG whole cell lysate
C
FLAG-FOXP1
Thiamet-G
IB: O-GlcNAc
IB: FLAG
IP:FLAG
* non-specific
D
FLAG-FOXP1
HA-ATRIP
IB: ATR
IB: HA
IB: FLAG
IP:FLAG
INPUT
F
Ratio IdU/CldU
shNC
shFOXP1
FLAG-FOXP1
G
forkhead
RPA binding
ssDNA binding
ATR-ATRIP binding
OGT binding
E
S phase population (%)
CON
CPT
siNC
siFOXP1
si+FOXP1 WT
si+R465G
si+R514C
si+R465T
si+R514H
IB: FOXP1
IB: Actin

 © The Author(s)

Figure EV5. The FOXP1 forkhead domain is essential for its function during conditions of replication stress.

(**A**) H1975 cells expressing FLAG-tagged FOXP1 or its mutants were subjected to immunostaining using FLAG-specific antibody, and the nuclei were detected with DAPI. (**B**) HEK293T cells transfected with FLAG-tagged FOXP1 or its mutants were treated with 2 mM HU for 1 h before chromatin fractionation. (**C**) HEK293T cells transfected with FLAG-tagged FOXP1 or its deletion mutants were preincubated with Thiamet-G before immunoprecipitation and then immunoblotting using the indicated antibodies. *, non-specific signal. (**D**) HEK293T cells transfected with FLAG-tagged FOXP1 or its deletion mutants and HA-tagged ATRIP were subjected to immunoprecipitation and then immunoblotting using the indicated antibodies. (**E**) FOXP1 knockdown HEK293 cells transfected with wild-type FLAG-FOXP1 or its pathogenic mutants were incubated with 100 nM CPT for 8 h or left untreated before harvested for PI staining and flow cytometric analysis. The percentage of S phase population was analyzed, mean ± SD (biological replicates, $n = 3$) is shown. *****P* < 0.0001, *P* values were calculated by two-way ANOVA, followed by Sidak's test. *P* value: siNC vs siFOXP1, 2.75e-008; siFOXP1 vs siFOXP1 + FOXP1 WT, 1.28e-007; siFOXP1 + FOXP1 WT vs siFOXP1 + R465G, 1.50e-006; siFOXP1 + FOXP1 WT vs siFOXP1 + R514C, 3.66e-006; siFOXP1 + FOXP1 WT vs siFOXP1 + R465T, 6.25e-007; siFOXP1 + FOXP1 WT vs siFOXP1 + R514H, 7.92e-006. (**F**) Statistical analysis of the IdU/CldU ratio mean in Fig. 5F, mean ± SD (biological replicates, $n = 3$) is shown. ***P* < 0.01, ****P* < 0.001, *P* values were calculated by one-way ANOVA, followed by Dunnett's test. *P* value: shNC vs shFOXP1, 0.0013, shFOXP1 vs shFOXP1+WT, 0.0008; shFOXP1+WT vs shFOXP1 + R465G, 0.0004; shFOXP1+WT vs shFOXP1 + R514C, 0.0005; shFOXP1+WT vs shFOXP1 + R465T, 0.0004; shFOXP1+WT vs shFOXP1 + R514H, 0.0004. (**G**) Functions of FOXP1 domains identified in this study. Source data are available online for this figure.

© The Author(s)
